# Wearable Electronics for Precision Diagnosis Through Advanced Manufacturing and Integration

**DOI:** 10.1007/s40820-026-02311-8

**Published:** 2026-08-07

**Authors:** Xia Gong, Ying Zheng, Xuyin Ding, Jie Gao, Bolang Cheng, Xinyi Shao, Jian Li, Lelun Jiang, Hossam Haick, Jian Yang

**Affiliations:** 1 Department of Biomedical Engineering, School of Electrical Engineering, University of South China, Hengyang, 421002 People’s Republic of China; 2 Department of Chemical Engineering, Russell Berrie Nanotechnology Institute Technion-Israel Institute of Technology, 3200003 Haifa, Israel; 3 School of Biomedical Engineering, Shenzhen Campus of Sun Yat-Sen University, Shenzhen, 518107 People’s Republic of China

**Keywords:** Wearable electronics, Biosensors, Advanced manufacturing, Heterogeneous integration, Precision diagnosis

## Abstract

Wearable electronics are rapidly transforming healthcare by enabling continuous, real-time monitoring of physiological and molecular signals directly at the point of need. This shift supports a transition from episodic, generalized care toward precision diagnosis, where individualized, longitudinal data guide early detection, risk stratification, and treatment decisions. Central to this transformation is the convergence of advanced manufacturing and heterogeneous integration strategies, which enable the development of compact, multimodal, and highly conformable diagnostic platforms. In this review, we examine how advances in materials engineering, micro/nanofabrication, and system integration have transformed wearable devices from single-parameter sensors into fully integrated, multimodal diagnostic systems. We outline key architectures and transduction mechanisms and highlight manufacturing approaches such as printing, 3D fabrication, photolithography, and laser writing. We emphasize heterogeneous integration strategies that combine sensing, electronics, power, and communication into skin-conformal platforms for long-term use. These advances enable precision diagnostics through continuous monitoring, multimodal data fusion, and individualized baselines. We also outline key challenges to clinical translation and discuss future directions toward robust, scalable, and clinically actionable systems, providing a strategic outlook for next-generation wearable electronics in precision diagnosis.

## Highlights

- Multimodal sensing strategies—electrical, electrochemical, transistor-based, and optical—integrated with AI-enabled interpretation establish a systems-level framework for continuous, individualized precision diagnosis across metabolites, electrophysiological signals, and molecular biomarkers in diverse biofluids.
- Advanced manufacturing and heterogeneous integration strategies, spanning printing, photolithography, 3D fabrication, and laser writing, enable skin-conformal platforms that unify sensing, signal transduction, power, and wireless communication into compact, long-term wearable diagnostic systems.
- Wearable diagnostic platforms demonstrate broad clinical applicability across neurological, cardiovascular, respiratory, ocular, musculoskeletal, oncological, diabetic, and inflammatory conditions; by enabling longitudinal multi-parameter data fusion and personalized physiological baselines, they provide a clinically actionable pathway for early disease detection, risk stratification, and real-time health management.

## Graphical abstract

**Figure:**
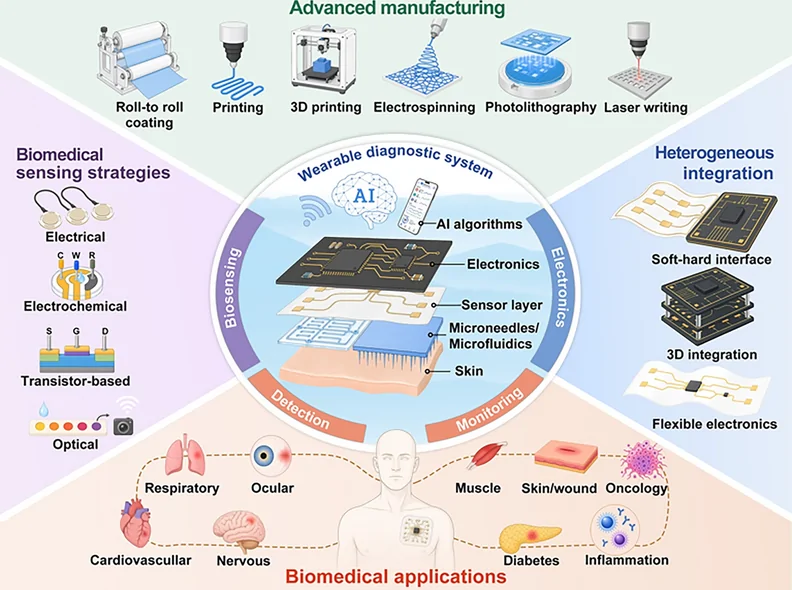

## Introduction

The increasing global burden of chronic diseases, such as diabetes, cardiovascular disease, and cancer, together with the rapid growth of the aging population, has become a major public health challenge worldwide [1–3]. At the same time, substantial disparities in access to diagnostic, therapeutic, and post-treatment management persist across regions [4, 5]. Despite these growing demands, current healthcare systems remain largely based on centralized, one-size-fits-all models that are increasingly inadequate for delivering comprehensive, affordable, and consistently high-quality care. To address these limitations, the concept of precision diagnosis has emerged as a transformative framework in modern healthcare [6–11]. Precision diagnosis aims to identify disease onset, progression, and heterogeneity at the individual level by leveraging continuous, high-resolution, and multi-parameter data. However, existing clinical practices are predominantly episodic and rely on isolated measurements obtained in controlled settings, limiting their ability to capture dynamic physiological changes, interindividual variability, and early-stage disease signal [8, 12–14]. As a result, these approaches fall short of fully realizing the potential of precision diagnosis. In response, healthcare is undergoing a profound shift toward wearable health devices and digital diagnostics [15–18]. These technologies enable continuous and longitudinal monitoring of individual health status, facilitate early detection of clinically significant physiological alterations, and expand access to advanced diagnostic capabilities, particularly in resource-limited settings [19, 20].

Wearable diagnostic systems are integrated analytical platforms that consolidate biosensor functionalities with miniaturized electronic components, mobile computing interfaces, and machine learning (ML) algorithms for automatic data interpretation and outcome prediction [21]. By analyzing a wide range of biofluids, these systems enable molecular-level health assessment in formats that can be worn directly on the body or incorporated into garments and body-worn accessories, allowing continuous tracking of subtle temporal fluctuations in physiological status and supporting precision diagnosis [22–24]. In this context, precision diagnosis is not limited to the detection of individual biomarkers but refers to a clinically actionable framework enabled by continuous, highly sensitive, and selective monitoring of molecular targets in complex biological matrices [13, 25, 26]. Such capabilities provide deeper insight into underlying biochemical processes associated with health and disease and support personalized assessment of disease onset, progression, and prognosis. In addition, the integration of molecular and electrophysiological signals enables multi-parameter data fusion, thereby overcoming the limitations of single-biomarker thresholds and improving differential diagnostic accuracy [22, 27, 28]. More importantly, these systems establish personalized reference frameworks that distinguish normal interindividual variability from true pathological changes within the same individual. Together, these capabilities define a precision wearable diagnostic system as a platform integrating high-fidelity sensing, longitudinal monitoring, multidimensional data interpretation, and individualized clinical assessment.

**Fig. 1:**
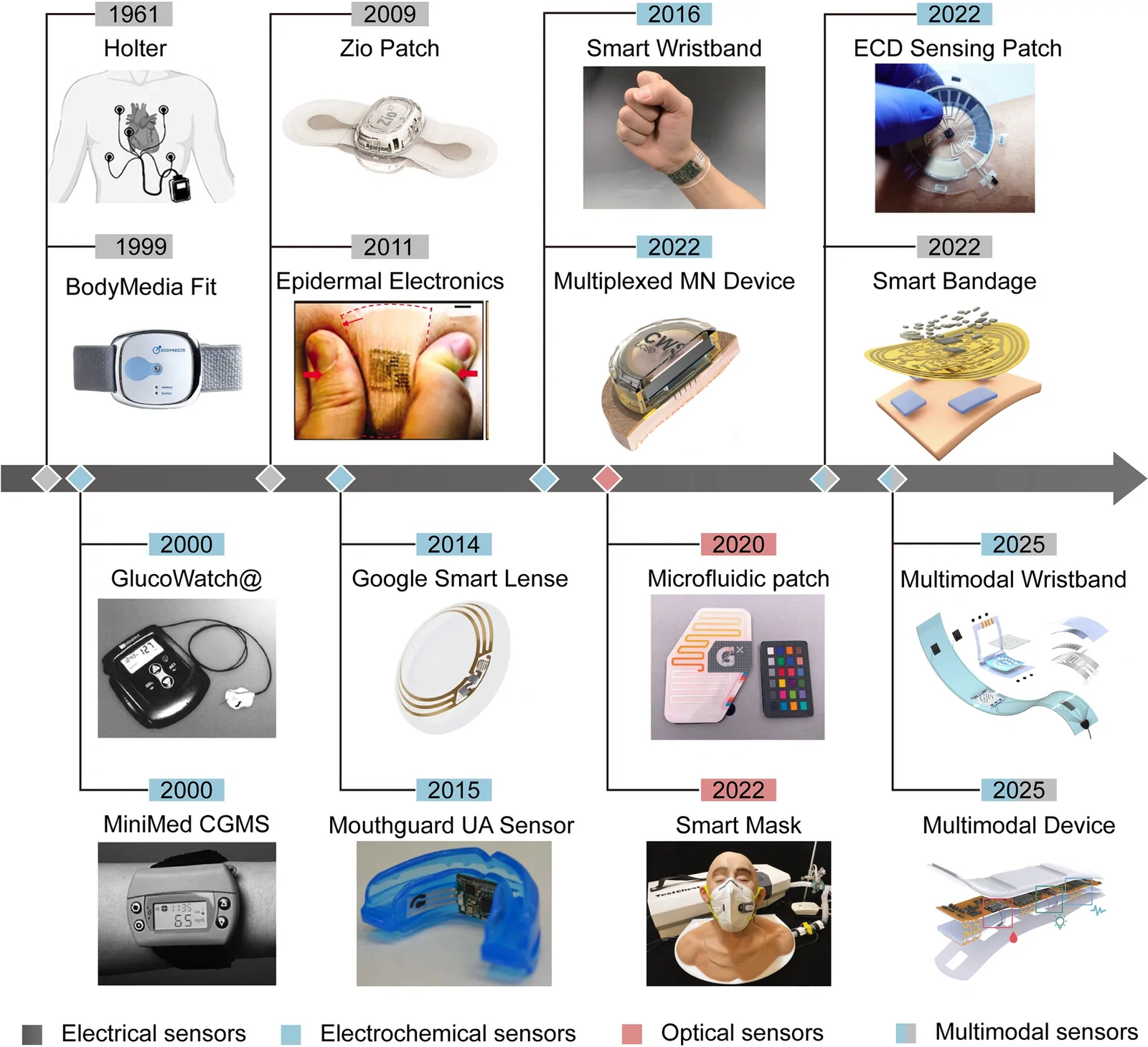
Evolution of wearable medical devices for health monitoring. Advances in materials, electronics, data analytics, and microfabrication have transformed wearable devices from single-signal monitors into integrated multimodal platforms for real-time chemical and physical sensing. Reproduced from Ref. [47] with permission, copyright 2026, American Chemical Society. Reproduced from Ref. [48] with permission, copyright 2020, Elsevier BV. Reproduced from Ref. [49] with permission, copyright 2005, BMJ Publishing Group Ltd. Reproduced from Ref. [50] with permission, copyright 2021, Springer Nature. Reproduced from Ref. [51] with permission, copyright 2024, Springer Nature. Reproduced from Ref. [52] with permission, copyright 2016, Springer Nature. Reproduced from Ref. [53] with permission, copyright 2015, Elsevier BV. Reproduced from Ref. [54] with permission, copyright 2016, Springer Nature. Reproduced from Ref. [55] with permission, copyright 2022, Springer Nature. Reproduced from Ref. [56] with permission, copyright 2020, American Association for the Advancement of Science. Reproduced from Ref. [57] with permission, copyright 2021, Springer Nature. Reproduced from Ref. [58] with permission, copyright 2022, Springer Nature. Reproduced from Ref. [59] with permission, copyright 2023, Springer Nature. Reproduced from Ref. [60] with permission, copyright 2025, Springer Nature. Reproduced from Ref. [61] with permission, copyright 2025, Springer Nature

Wearable medical systems have emerged for several decades, as early as the 1960s, the Holter monitor pioneered the ambulatory recording of cardiac electrical signals [29]. In recent era, the convergence of Internet infrastructure, semiconductor technology, and big data has driven a fundamental transformation in wearable system architectures, catalyzing the development of fully integrated biochemical sensing platforms capable of extracting rich molecular information (Fig. 1) [30]. Operating under diverse transduction mechanisms, these systems employ the following principal sensing modalities: direct electrical sensors, including potentiometric, resistive, capacitive, and inductive sensors; electrochemical sensors based on biochemical interactions or catalytic redox reactions; and optical sensor**s.** Each modality offers distinct advantages: potentiometric and resistive sensors are amenable to array-based signal acquisition [26, 31–36]; capacitive sensors offer rapid response kinetics [37, 38]; field-effect transistors (FETs) and their derivatives provide intrinsic signal amplification [9, 39, 40]; inductive sensors support non-contact measurement [41, 42]; electrochemical sensors combine analyte selectivity with straightforward miniaturization [43]; and optical sensors demonstrate superior electromagnetic interference immunity with trace-level analyte detection [44–46].

Despite the considerable promise of this technological vision, the transition from laboratory-scale prototypes to clinically viable diagnostic tools remains confronted by substantial engineering challenges. The central challenge lies in the heterogeneous integration of sampling fluidic, chemical, and electronic functional modules onto a single, skin-conformal substrate, which demands advanced fabrication and assembly strategies [5, 62]. Key challenges include balancing multimodal sensing capabilities with wireless data transmission and mechanical stretchability on polymeric and flexible substrates, achieving precise micro- and nanoscale structural fabrication, and mitigating cross-sensitivity, interference in complex biological matrices, and mechanical mismatches between compliant sensors and rigid circuit components [47, 63–65].

Despite substantial advances in the fabrication and integration of wearable electronics, previous reviews primarily focused on material systems or specific biomarkers and disease applications [22, 66–68]. Comprehensive reviews dedicated to recent innovations in manufacturing and integration methodologies for wearable electronics remain scarce [69]. In response to this gap, this review systematically examines recent progress in fully integrated wearable electronics for precision and personalized diagnostics. Specifically, we first present the overall system architecture and the underlying transduction mechanisms, as these foundational considerations are essential to the rational design of integrated sensing platforms. We subsequently summarize advanced fabrication and integration strategies with respect to their contributions to system miniaturization, mechanical conformability, and performance consistency. Finally, we highlight the clinical translational potential of these systems, demonstrating their utility across a broad spectrum of clinical domains, spanning neurological, cardiovascular, respiratory, and ocular disorders, as well as oncology, diabetes, and inflammatory and immune-mediated conditions. By delineating the pathway from foundational fabrication principles to clinical translation, this review aims to provide a strategic roadmap for the next generation of wearable precision diagnostic platforms.

## Construction of Wearable Diagnostic System

Wearable diagnostic systems are engineered for the sporadic or continuous non-, or minimally invasive acquisition of human physiological and biochemical data [70]. These platforms span a broad range of forms, including skin-mounted patches [71], functional tattoos [72], mouthguards [73], smart contact lenses [74, 75], smart rings [76], and smartwatches [77]. By coupling point-of-care testing (POCT) capabilities with mobile connectivity, wearable diagnostics support real-time tracking of individual health metrics, facilitating early identification of disease-associated deviations and providing actionable inputs for clinical decision-making. A wearable diagnostic system primarily comprises three parts: biomedical sensors, wearable electronics, and decision-making algorithms. As the foundational component of the platform, sensors translate physiological or biochemical cues into quantifiable signals through physical and/or biochemical recognition mechanisms. These sensing events are subsequently transduced into electrical or optical outputs and conditioned by microelectronic modules, such as amplification, filtering, and analog-to-digital conversion [78]. The processed data are then transmitted to a user terminal, where the decision-making algorithms perform data analytics, feature extraction, anomaly classification, and concentration regression. Ultimately, the data or diagnostic results are presented to the user or integrated into digital healthcare infrastructures [79].

### Wearable Biomedical Sensing Strategies

In the design of wearable diagnostic systems, the selection and engineering of sensors are paramount, as sensors are required to enable direct, on-body, real-time measurements in a reagent-free and continuous manner during wear [19, 80]. Consequently, protocols that require off-body handling, such as additional washing or chemical addition steps, are generally incompatible with the on-body operation [81]. Currently, electrical and electrochemical sensors remain the most widely adopted modalities in the medical field, largely due to their compatibility with established large-area printing techniques. Nevertheless, driven by the growing demand for multiplexed and more comprehensive diagnostic capabilities, alongside advances in heterogeneous integration and miniaturized optical packaging, platforms integrating optical and Raman sensors have also begun to emerge. As summarized in Table 1, each sensing modality offers a distinct balance between sensitivity, selectivity, manufacturability, power consumption, and long-term stability across different biomedical applications.

**Table 1:** Comparative analysis of sensing technologies for wearable biomedical sensors

Sensing technology	Target analytes	Biofluids	Wearable formats	Advantages	Limitations	References
Electrical sensing	Biopotentials, temperature, humidity motion, mechanical force, breathing, gas, VOCs	Exhaled breath, epidermal molecular flux	Epidermal patch, tattoo, wristband, necklace, ring, insoles, mask, wound dressing, textile-embedded device, headset	Exceptionally simple structures with low manufacturing costs and ultralow power consumption; easy to implement in large-area, high-density arrays	Motion artifacts, stringent requirements for skin–electrode contact impedance, affected by electromagnetic interference	[161–165]
Electrochemical sensing	Metabolites, electrolytes, proteins, hormones, nutrients, amino acids, cytokines, peptides, nucleic acids, neurotransmitters, antibiotics, pathogens	Sweat, ISF, saliva, tear, urine, wound fluid	Microfluidic sweat patch, transdermal MN patch, tattoo, wristband, mouth guard, contact lenses, wound dressing, smart mask	Excellent sensitivity and selectivity; rapid response; easily miniaturized; linear output	Susceptible to biofouling, degradation over time, unstable output, limited by temperature and pH	[67, 166–173]
Transistor-based sensing	Metabolites, electrolytes, proteins, hormones, nutrients, nucleic acids, antibiotics	Sweat, ISF, saliva, tear, urine, wound fluid	Microfluidic sweat patch, smartwatches, textile-embedded device	Ultra-high sensitivity, intrinsic signal amplification, amenable to high-density and flexible integration	Nonspecific adsorption, baseline drift, surface modification challenges	[143, 145, 146, 174, 175]
Optical sensing	Hemodynamics, metabolites, electrolytes, ROS, pO_2_, cortisol, target enzymes, antibodies, nucleic acids	Sweat, ISF, saliva, tear, urine, wound fluid	Smartwatch, tattoo, skin patch, wound dressing	Noninvasive, immune to electromagnetic interference, cost-effectiveness, high sensitivity and selectivity	Vulnerable to environment interference, fluorescence and SERS techniques require integrated emitters/detectors, increasing power consumption and device volume	[66, 158, 176–178]

#### Electrical Sensors

**Fig. 2:**
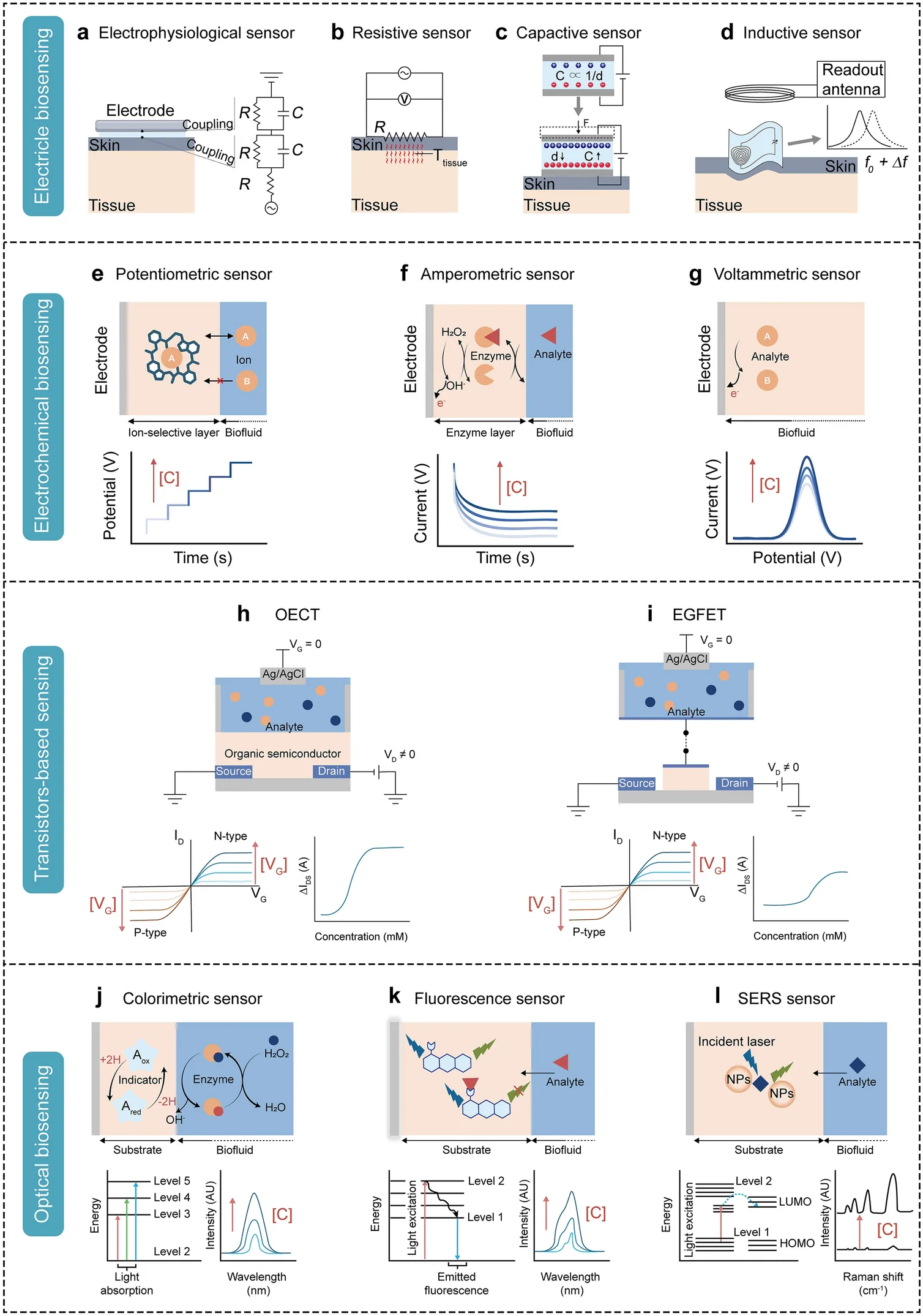
Principal signal transduction mechanisms in wearable diagnostic systems. **a-d** Recognition and signal transduction principles for wearable electrical biosensors, including electrophysiological, resistive, capacitive, and inductive sensors, which primarily rely on monitoring changes in circuit component parameters in response to external stimuli. **e–g** Recognition and signal transduction principles for wearable electrochemical biosensors, including potentiometric, amperometric, and voltammetric sensors, which convert the analyte concentration into potential or current signals through ion exchange or redox reactions at the electrode-biofluid interface. Reproduced from Ref. [47] with permission, copyright 2026, American Chemical Society. **h–i** Recognition and signal transduction principles for wearable transistor-based biosensors, including OECT and EGFET sensors, which convert the analyte concentration into variations of ∆*I*_DS_ signals induced by biochemical reactions at the semiconductor/extended-gate-biofluid interface. **j–l** Recognition and signal transduction principles for wearable optical biosensors, including colorimetric, fluorescence, and surface-enhanced Raman spectroscopy sensors, which quantify analytes by capturing changes in light absorption, fluorescence emission, or Raman spectral intensity induced by specific biochemical reactions

Electrical sensors used in wearable systems can be grouped into direct biopotential sensors and indirect sensors that convert mechanical, thermal, or other physical stimuli into changes in resistance, capacitance, or inductance. Biopotential sensors measure differential electrical signals generated by neuromuscular activity [82, 83]. Their performance depends strongly on stable electrode–skin coupling, which is shaped by electrode conformability, skin topography, and the interfacial impedance. This interface is commonly represented by a simplified resistor–capacitor (RC) circuit, in which electrode design alters the effective conductance and capacitance values [84, 85]. As illustrated in Fig. 2a, for a given electrode type, coupling is often dominated by a single RC element (Fig. 2b), allowing the admittance to be approximated as:

$${\mathrm{Y}}_{{{\text{c }}\left( {{\mathrm{j}}\omega } \right)}} {\text{ = g}}_{{\mathrm{c}}} {\text{ + j}}\omega {\mathrm{C}}_{{\mathrm{c}}}$$

where the complex admittance (*Y*_c (j*ω*)_) combines the interfacial conductance (*g*_c_) and the frequency-dependent capacitive susceptance (j*ωC*_c_). Time-varying physiological signals are transferred through this interface and form differential voltages. Robust acquisition is then achieved by differential amplification and a filtering module with a high common-mode rejection ratio (CMRR) [86]. During long-term wear, however, electrophysiological recordings are highly susceptible to motion artifacts, sweat accumulation, adhesive degradation, and electrode–skin impedance drift, all of which can alter coupling admittance and reduce signal fidelity.

Indirect resistive sensors are widely used because they are simple, inexpensive, and compatible with printable conductive composites. Their structural simplicity often allows for straightforward monolithic integration into flexible substrates via roll-to-roll (R2R) processing or screen printing. In temperature sensing, resistance changes reflect temperature-dependent carrier transport (Fig. 2b) [87]. In strain sensors, deformation changes both sensor geometry and conductive pathways, including crack opening, contact separation, or percolation-network rearrangement (Fig. 2c) [68, 88].

Capacitive sensors generally provide fast response and low power consumption. They measure stimulus-induced changes in plate spacing, overlap area, or dielectric permittivity (Fig. 2d) [89]. Microstructured dielectrics produced by soft lithography or laser ablation can markedly increase sensitivity under small loads [90], supporting applications from cardiovascular implants [91] to tendon-repair monitoring [92].

Inductive sensors, often designed as planar spiral coils, are less explored in wearable diagnostics [93, 94]. Bending or stretching changes coil geometry and magnetic-flux coupling, producing a measurable inductance shift [95, 96]. Related inductor–capacitor (LC) resonant sensors are attractive for passive wireless implants and have been successfully used to monitor bone-prosthesis strain, deep-wound integrity, gastric leakage, and tissue micromotion [97–99]. For resistive, capacitive, and inductive sensors, long-term reliability is often limited by mechanical fatigue of conductive networks, dielectric aging, encapsulation failure, humidity-dependent parasitic responses, and deformation-induced baseline drift, highlighting the need for standardized cyclic loading and environmental stability tests.

In practical biomedical applications, electrical sensors are particularly suitable for targets that do not require direct liquid-phase biochemical interactions. Biopotential sensors record surface potential generated by ion-current-driven neural, muscular, or cardiac activity through direct skin contact [100]. By contrast, resistive, capacitive, and inductive sensors mainly respond to physical or quasi-physical stimuli, such as mechanical deformation, pressure, temperature, humidity, gas exposure, or epidermal molecular flux, rather than to specific reactions of dissolved biomarkers in biofluids [101]. This relative independence from biofluid sampling and complex interfacial chemistry fundamentally defines their application scope in wearable diagnostics. It also explains their simple device architectures, low manufacturing cost, ultralow power consumption, and ease of integration into large-area, high-density arrays, making them well suited for skin-mounted patches, textile-embedded devices, mask-integrated platforms, and breath-sampling wearable systems.

#### Electrochemical Sensors

Electrochemical sensors differ from electrical sensors primarily through their functionalized electrode interfaces and analyte-specific interfacial reactions. Recognition elements or catalytic layers, such as enzymes, proteins, or antibodies, convert target binding or reaction events into changes in potential, current, accumulated charge, or medium conductivity [102]. Benefiting from low cost, ease of operation, structural compactness, and minimal dependence on reaction volumes, and their excellent compatibility with additive manufacturing techniques like inkjet and stencil printing, electrochemical sensing technologies have been widely applied in the field of wearable disease diagnostics. For long-term wearable operation, however, electrochemical interfaces are particularly vulnerable to biofouling, recognition-layer degradation, mediator leaching, reference-electrode drift, and matrix-dependent interference, which can collectively compromise sensitivity, selectivity, and baseline stability.

Potentiometric sensors quantify ionic species by measuring the equilibrium potential between an ion-selective working electrode (WE) and a reference electrode (RE) under near-zero-current conditions (Fig. 2e). The response arises from the phase-boundary potential at the ion-selective membrane (ISM)-solution interface and follows the Nernst equation:

$${\text{E = E}}^{{0}} { + }\frac{{{\mathrm{RT}}}}{{{\mathrm{zF}}}}{\text{ln a}}$$

where E represents the potential of the WE relative to the RE, *E*^0^ is the standard potential, *R* is the universal gas constant, T is the absolute temperature, *z* is the ionic charge number, F is the Faraday constant, and *a* is the ionic activity quotient in the aqueous and solid membrane phases [103]. The measured potential therefore varies logarithmically with target-ion activity. In wearable ion-selective electrodes, analytical performance depends mainly on membrane selectivity, the stability of the ion-to-electron transduction layer, and the reference electrode [104–106]. Membrane thickness, electrode miniaturization, and ionophore-ion binding strength can be adjusted through fabrication to tune response dynamics and the useful concentration range [107–109]. During prolonged use, the long-term stability of both the ISM and RE becomes decisive, because ion leaching, water-layer formation, sweat composition changes, and Cl^−^ depletion can induce potential drift and reduce the reliability of longitudinal ion monitoring.

Amperometric sensors detect analytes by applying an oxidation or reduction potential to the working electrode and measuring the resulting Faradaic current [110] (Fig. 2f). In a three-electrode configuration, the WE is functionalized with a recognition/catalytic layer, the counter electrode (CE) completes the current circuit, and the RE stabilizes the applied potential. This co-planar three-electrode layout is easily mass-produced on flexible thermoplastic elastomers or polyimide substrates. The Faradaic current scales with analyte concentration, enabling real-time quantification when mass transport and electron transfer conditions are controlled. Under diffusion-limited conditions, the current i can be expressed as:

$${\text{i = }}\frac{{{\text{n FA}}\left( {{\mathrm{C}}_{{{\mathrm{bulk}}}} {\text{ - C}}_{{\text{x = 0}}} } \right)}}{{\updelta }}$$

where *n* is the number of transferred electrons, *F* is the Faraday constant, *A* is the electroactive area, *D* is the diffusion coefficient, *δ* is the diffusion-layer thickness, *C*_bulk_ is the bulk concentration, and *C*_x=0_ is the surface concentration [111]. For wearable diagnostics, amperometric sensor design should emphasize efficient electron transfer, robust operation in complex biofluids, and reproducible measurement. Performance is mainly governed by enzymatic or non-enzymatic reaction kinetics, substrate diffusion, and electron transfer between the recognition or catalytic element and the electrode. Accordingly, nanostructured or porous electrodes are widely used to enlarge the effective surface area and reduce transport limitations [103], while electron mediators or conducting polymers can shorten electron transfer pathways and support quantification across the μM to tens of mM concentration ranges typical of biofluids [112]. Long-term on-body stability further requires robust, manufacturable immobilization strategies, such as covalent bonding or polymer/hydrogel entrapment, to reduce mediator leaching and enzyme deactivation [113]. Selectivity and stability can be improved by minimizing the operating potential where feasible and incorporating permselective membranes, antifouling coatings, calibration channels, or auxiliary sensors for real-time compensation [114–117]. In long-term monitoring, enzymatic deactivation, biofouling-induced diffusion barriers, mediator loss, and temperature- or pH-dependent reaction kinetics remain major sources of signal drift, making drift correction and in situ calibration essential for clinically reliable amperometric wearables.

Voltammetric methods, including linear sweep voltammetry (LSV), cyclic voltammetry (CV), square wave voltammetry (SWV), differential pulse voltammetry (DPV), and stripping voltammetry (SV) [118], apply a programmed potential sweep between the WE and the RE (Fig. 2g). Pulsed techniques such as SWV and DPV further improve analytical sensitivity by increasing the Faradaic-to-capacitive current ratio through time-resolved potential excitation. By sampling current immediately before and at the end of each voltage pulse, these methods suppress background charging currents and enable precise quantification of analytes at nanomolar concentrations [119]. Compared to potentiometric and amperometric approaches, voltammetric methods are particularly suitable for multiplexed detection. Different electroactive species can produce characteristic peaks at distinct redox potentials, allowing peak position to support analyte discrimination and peak current or peak area to support quantification [120, 121]. Thus, voltammetric measurements are more sensitive to excitation-waveform parameters, sampling synchronization, and long-term reference-electrode stability. To address these challenges, recent work has increasingly focused on electrode interfacial engineering. Passive antifouling strategies commonly use hydrophilic or charge-neutral polymer coatings to reduce protein adsorption through steric and hydration effects, whereas active strategies remove adherent interferents by applying external stimuli that generate shear forces exceeding adhesion forces [122]. Despite these advances, prolonged voltammetric operation still requires systematic evaluation of electrode fouling, waveform-dependent artifacts, RE drift, and peak-position shifts under repeated scanning in real biofluids.

The broad biofluid compatibility of electrochemical sensors, spanning sweat, interstitial fluid (ISF), saliva, tears, urine, and wound fluid (Table 1), reflects the versatility of interfacial redox and ion-exchange chemistry for monitoring electrolytes, metabolites, hormones, drugs, and inflammatory markers across different physiological compartments [67]. Sweat is the most accessible target for noninvasive electrochemical wearables and is well suited to printed epidermal patches and wristband devices, because it can be collected during exercise, thermal stimulation, or iontophoresis and readily integrated with microfluidic channels [123]. However, its dependence on secretion rate, dilution, and interindividual gland variability can weaken its correlation with blood biochemistry [124]. Saliva and tears provide complementary access to oral and ocular microenvironments, as well as selected systemic biomarkers, but their small sample volumes, rapid turnover, mucins, microbial contamination, and strict comfort or transparency requirements make stable electrode integration more challenging. By contrast, ISF offers a closer biochemical relationship to blood, but its sub-stratum-corneum location requires minimally invasive access, making microneedle (MN) electrodes a suitable interface for stable dermal fluid monitoring [117, 125]. Wound fluid and urine further expand electrochemical monitoring toward local inflammation, infection, and renal function, although wound exudate is protein-rich, whereas urine collection is typically intermittent rather than continuous [125, 126]. Therefore, the selection of a monitoring strategy for a specific analyte should consider not only its electrochemical properties, but also biofluid accessibility, available sample volume, and matrix complexity.

#### Transistor-Based Sensors

Transistor-based sensors are three-terminal devices in which a gate modulates charge transport through a semiconducting channel between the source and drain [127]. Organic field-effect transistors (OFETs) have emerged as transformative platforms in POCT diagnostics, underpinned by their exceptional material versatility, intrinsic signal amplification and scalable low-cost fabrication, particularly through solution-based processing techniques that allow for direct printing onto flexible films, and readily tunable device architectures [128, 129]. Biochemical interactions at the sensing interface can shift the local electrostatic environment or alter charge transport, allowing small recognition events to be converted into amplified current changes. Their scalable, miniaturized architecture further facilitates seamless device integration and high-density array patterning, reinforcing their role as a powerful biosensing platform for multiplexed, parallel bioanalysis. The source–drain current *I*_DS_ is related to the gate voltage (*V*_G_) by:

$${\mathrm{I}}_{{{\mathrm{DS}}}} { = }\left\{ {\begin{array}{*{20}c} {\frac{{\mathrm{W}}}{{\mathrm{L}}}{\mu C}_{{\mathrm{i}}} \left( {{\mathrm{V}}_{{\mathrm{G}}} {\text{ - V}}_{{\mathrm{t}}} } \right){\mathrm{V}}_{{{\mathrm{DS}}}} {\text{ V}}_{{{\mathrm{DS}}}} \ll {\mathrm{V}}_{{\mathrm{G}}} {\text{ - V}}_{{\mathrm{t}}} } \\ {\frac{{\mathrm{W}}}{{{\mathrm{2L}}}}{\mu C}_{{\mathrm{i}}} \left( {{\mathrm{V}}_{{{\mathrm{G}} }} {\text{ - V}}_{{\mathrm{t}}} } \right)^{{2}} {\text{ V}}_{{{\mathrm{DS}}}} {\text{ > V}}_{{\mathrm{G}}} {\text{ - V}}_{{\mathrm{t}}} } \\ \end{array} } \right.$$

where *I*_DS_, *V*_G_, *W*, *L*, *μ*, *C*_i_, and *V*_t_ denote the source-drain current, gate voltage, channel width, channel length, carrier mobility, gate-dielectric capacitance, and threshold voltage, respectively [130]. In biosensing, the *I*_DS_ can be modulated by interfacial charge, impedance changes at the sensing layer-channel interface, or local chemical reactions such as proton or redox generation [131, 132]. Wearable OFET biosensor design requires coordinated optimization across the sensing interface, probe selection and immobilization, analytical performance, and practical deployment. The sensing interface is central to this design framework. Semiconducting layers, including conjugated polymers such as poly(3-hexylthiophene) (P3HT) and poly(dithiobenzodithiophene-co-thienothiophene) (PDBT-co-TT), as well as small-molecule semiconductors such as 2,7-dioctyl[1]benzothieno[3,2-b][1]benzothiophene (C8-BTBT-C8), can support direct probe immobilization and ultrasensitive analyte detection, including attomolar-to-femtomolar detection of protein [133–137]. Extended-gate field-effect transistors (EGFETs) provide an alternative architecture in which the sensing region is physically separated from the driving transistor. This separation simplifies surface functionalization and improves operational stability in complex media, as shown by enzyme-modified extended gates for long-term lactate monitoring [138]. For charge-transport optimization, functionalization strategies should preserve π–π stacking in the organic semiconductor. Bilayer architectures or direct blending of probe molecules with the semiconductor can balance efficient probe immobilization with high carrier mobility [139–141]. Long-term wearable operation also requires antifouling interfaces, encapsulation of non-sensing regions, reference or differential transistor designs, and baseline-drift correction. These strategies are particularly important because nonspecific adsorption, ion screening, threshold-voltage drift, semiconductor swelling or degradation, and instability at the semiconductor–electrolyte interface can obscure weak biochemical signals.

Transistor-based sensors share the broad biofluid compatibility of electrochemical sensors but differ in their signal transduction mechanism. Their main advantage is intrinsic amplification: Small changes in gate potential, interfacial charge, or channel impedance induced by analyte recognition are transduced into amplified *I*_DS_ responses [142, 143]. This built-in gain can improve sensitivity toward low-abundance proteins and nucleic acids, often in the femtomolar-to-picomolar range, while reducing the need for complex external amplification circuits in miniaturized wearable systems [144–146]. This gain comes, however, with more stringent demands on surface engineering and device stability. Protein and cell adsorption on the channel or gate can directly alter current output and generate false analyte signals, while Debye screening at physiological ionic strength attenuates charge-based detection, posing challenges in sweat, saliva, wound exudate, and other complex biofluids.

#### Optical Sensors

Optical biosensors provide contactless readout, high sensitivity, and strong signal-to-noise performance [147]. Optical sensors can detect physical and biochemical analytes by absorbance (colorimetric), emission (fluorescence or luminescence), or scattering (plasmonic) responses.

Colorimetric sensors are attractive for point-of-care and resource-limited settings because their signals can often be read visually or with a smartphone. They rely on analyte- or environment-induced changes in visible-light absorption from chromogenic materials (Fig. 2h). Representative mechanisms include plasmonic nanostructure aggregation or etching, enzyme or nanozyme catalysis that generates colored products, and molecular probes whose electronic structure changes after target recognition [148, 149]. These optical variations can be interpreted semi-quantitatively or quantitatively based on the Beer–Lambert law, using either direct visual inspection or smartphone-assisted analysis in RGB or HSV space [150]. These assays can be integrated into microfluidic sweat patches and manufactured by printing or laser cutting. Nevertheless, practical deployment of colorimetric sensors remains challenged by matrix-dependent interference and cross-reactivity, as well as by the inherent difficulty of achieving continuous, real-time monitoring. Additional sources of variability include nonuniform illumination, camera white-balance settings, color aging, sweat dilution, and skin-background effects, which can compromise quantitative interpretation during prolonged or repeated use.

Fluorescence-based sensors operate on photoluminescence, employing fluorescent probes–such as organic dyes, quantum dots, or fluorescent proteins–as signal transduction elements, whose intensity, lifetime, quantum yield, or emission wavelength changes after excitation and target interaction (Fig. 2i) [151]. This transduction mechanism, together with intrinsically fast optical kinetics and probe-dependent molecular specificity, confers high analytical sensitivity and rapid response, making fluorescence sensing particularly suitable for detecting low-abundance and trace-level analytes [152]. In POCT systems, smartphone-integrated or handheld devices become prominent platforms for fluorescence signal acquisition [153]. Representative implementations include fluorescent contact lenses for tear-glucose monitoring [154] and hydrogel wound dressings for wound pH monitoring [155]. Despite these advances, the broader adoption of fluorescence-based sensors in wearable systems remains constrained by hardware requirements. Fluorescence detection is inherently dependent on sophisticated optical hardware, including excitation light sources and photodetectors, typically accompanied by spectral filters. This instrument-dependent nature inevitably increases device bulk and power consumption, thus imposing critical bottlenecks for achieving high integration density and sustained on-body monitoring. Addressing this bottleneck requires advanced heterogeneous integration strategies to embed miniaturized vertical-cavity surface-emitting lasers, thin-film organic light-emitting diodes, and ultra-thin photodetectors directly onto pliable substrates. For long-term sensing, fluorescence platforms must also address photobleaching, excitation-intensity fluctuation, detector drift, and autofluorescence from tissues or biofluids, which can lead to baseline shifts and reduced quantitative accuracy.

Surface-enhanced Raman spectroscopy (SERS) sensors convert molecular information by capturing the intrinsic vibrational and rotational fingerprint spectra (Fig. 2j). Because these characteristic Raman signatures are fundamentally dictated by molecular structure, SERS can support label-free, rapid, and multiplexed biomarker analysis [156]. Furthermore, the incorporation of molecularly specific Raman labels or functionalized nanotags extends SERS utility to selective identification of individual target molecules [157]. However, SERS still depends on optical excitation, photodetection, and spectral analysis hardware, limiting portability and ease of integration. Compact wearable Raman systems therefore remain an important but still immature direction for wearable diagnostics. To date, compact wearable Raman spectrometers remain far from widespread application. In addition, SERS signals are sensitive to substrate reproducibility, hot spot degradation, laser alignment, local heating, and background scattering, making standardized substrate fabrication and stable optical coupling essential for reliable long-term wearable deployment.

The applicability of optical sensing modalities across biofluids is governed by both physical accessibility and optical compatibility. In microfluidic patches, colorimetric assays are particularly suitable for sweat and wound exudate, where relatively abundant analytes can generate visible color changes without complex instrumentation [66]. By contrast, optical monitoring of ISF is more challenging. ISF is located within the dermis, typically 200–2000 μm below the skin surface and therefore requires transdermal near-infrared interrogation [158]. Such approaches are constrained by strong water absorption, tissue scattering, and uncertainty in the optical path length. Tear provides a more favorable environment for optical sensing. Unlike electrochemical and transistor-based platforms, which usually require electrodes, conductive traces, or direct electrical contact with the sample, optical readouts can operate with very small fluid volumes and without wired interfaces at the ocular surface [159, 160]. Colorimetric and fluorescent probes can be incorporated into the hydrogel matrix while preserving lens transparency and visual function. The probes can then be excited and detected by compact optical readers, smartphone cameras, or co-planar miniaturized optical systems. This configuration enables noninvasive molecular monitoring in very small tear volumes without requiring optical penetration into deeper ocular tissues.

### Electronics Components

**Fig. 3:**
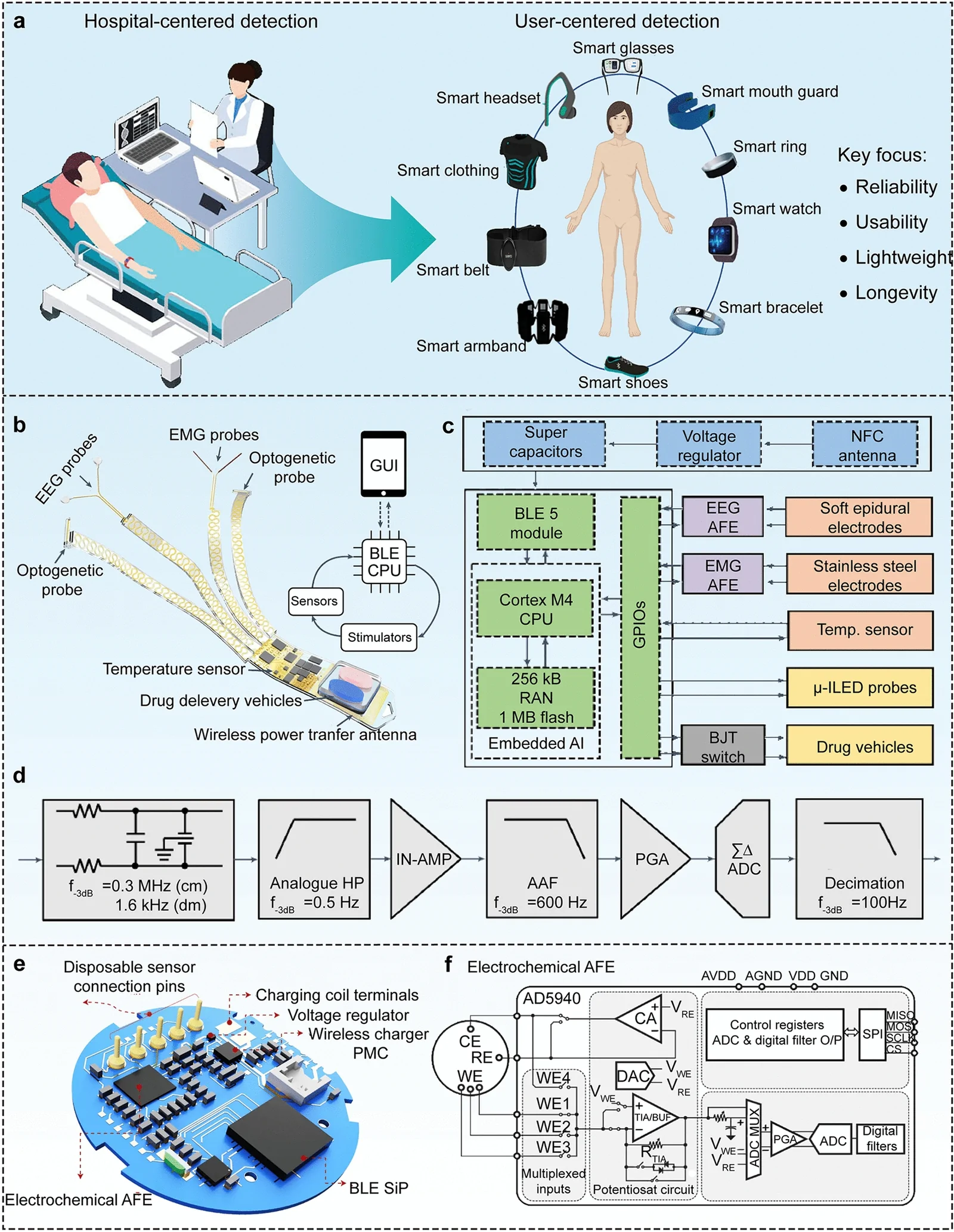
Electronics for wearable diagnostic systems. **a** Movement of healthcare monitoring from conventional clinic-based systems toward distributed, body-integrated wearable devices for daily use, with key design priorities including reliability, usability, lightweight construction, and long-term operation. Reproduced from Ref. [180, 181] with permission, copyright 2024, Wiley. **b** Schematic illustration of a wireless, battery-free device for the autonomous recording of electroencephalograms, electromyograms, and body temperature, as well as for closed-loop neuromodulation. **c** Electrical architecture of the device. **d** Signal conditioning workflow for high-fidelity acquisition of electrophysiological signals. Reproduced from Ref. [182] with permission, copyright 2023, Springer Nature. **e** MN-based wearable sensor system sensing system for continuous monitoring of multiple biomarkers, with major electronic components labeled. **f** Functional block diagram of the multichannel potentiostat AD5940. Reproduced from Ref. [55] with permission, copyright 2022, Springer Nature

Wearable diagnostic systems continuously collect biomarker profiles from different biofluids by integrating sensor arrays with power sources and electronic modules. Among these, the electronic subsystem plays a central role in multichannel signal acquisition, data fusion, and wireless transmission. The design philosophy of such devices should shift from hospital-centered paradigm to user-oriented framework, with a focus on reliability, usability, measurement accuracy, lightweight form factors, and extended battery life (Fig. 3a). Circuit design should therefore be tailored to the target use case and signal-processing objectives. At the outset, key constraints, including power budget, signal characteristics, conversion precision, device footprint, and wireless data-link requirements, should be defined to systematically inform component selection and circuit layout.

In monitoring-oriented circuit systems, the high-fidelity acquisition of analog signals from the sensor front-end is paramount to overall system performance. For low-amplitude, low-bandwidth electrophysiological signals such as electroencephalography (EEG), electrocardiography (ECG), and electromyography (EMG), instrumentation amplifiers featuring high input impedance, high CMRR, low input-referred noise, and tolerance to direct current (DC) offset are required (Fig. 3b-d) to guarantee signal fidelity and analytical precision within complex bioelectrical environments.

For electrochemical measurements, amplifiers with high input impedance, ultralow input bias current, and strong interference rejection capability are preferred. In such electrochemical systems, the potentiostat applies a programmed excitation voltage between the WE and the RE while suppressing voltage drift arising from polarization at the WE surface; accordingly, feedback control loops are commonly engineered to maintain a stable WE-RE potential. In wearable settings, a digital-to-analog converter (DAC) in a microcontroller unit (MCU) provides the target bias voltage, which is regulated via a differential amplifier to precisely control the potential between the RE and WE. The resulting faradaic current flowing between the CE and the WE is subsequently converted into a voltage signal by a transimpedance amplifier (TIA) and quantized by an analog-to-digital converter (ADC) (Fig. 3e–f). Critically, the ADC sampling rate and dynamic range dictate the temporal resolution of the system, ensuring the continuity of longitudinal tracking. For dynamic voltammetric techniques—including DPV, CV, and SW—operational amplifiers need higher bandwidth, and ADCs with faster sampling rates are required [179].

Following front-end acquisition and preliminary preprocessing by MCU, the data are transmitted to a host system via Internet of Things (IoT) networks. Bluetooth Low Energy (BLE) and Near-Field Communication (NFC) are among the most widely adopted protocols owing to their low power consumption and high integration maturity. NFC leverages near-field magnetic coupling with an external coil at 13.56 MHz to transfer both energy and data without an onboard power source, albeit with a practical communication range typically on the order of ~ 10 cm. By contrast, BLE operates in the 2.4 GHz band and can support data transmission up to ~ 100 m using miniature antennas, but it requires an internal power supply for sustained operation. Consequently, meticulous management of the overall power budget and the BLE duty cycle is essential to maximize the device’s operational lifespan, which is a fundamental prerequisite for the long-term accumulation of personalized physiological baselines.

### Algorithm-Enabled Wearable Diagnosis

**Fig. 4:**
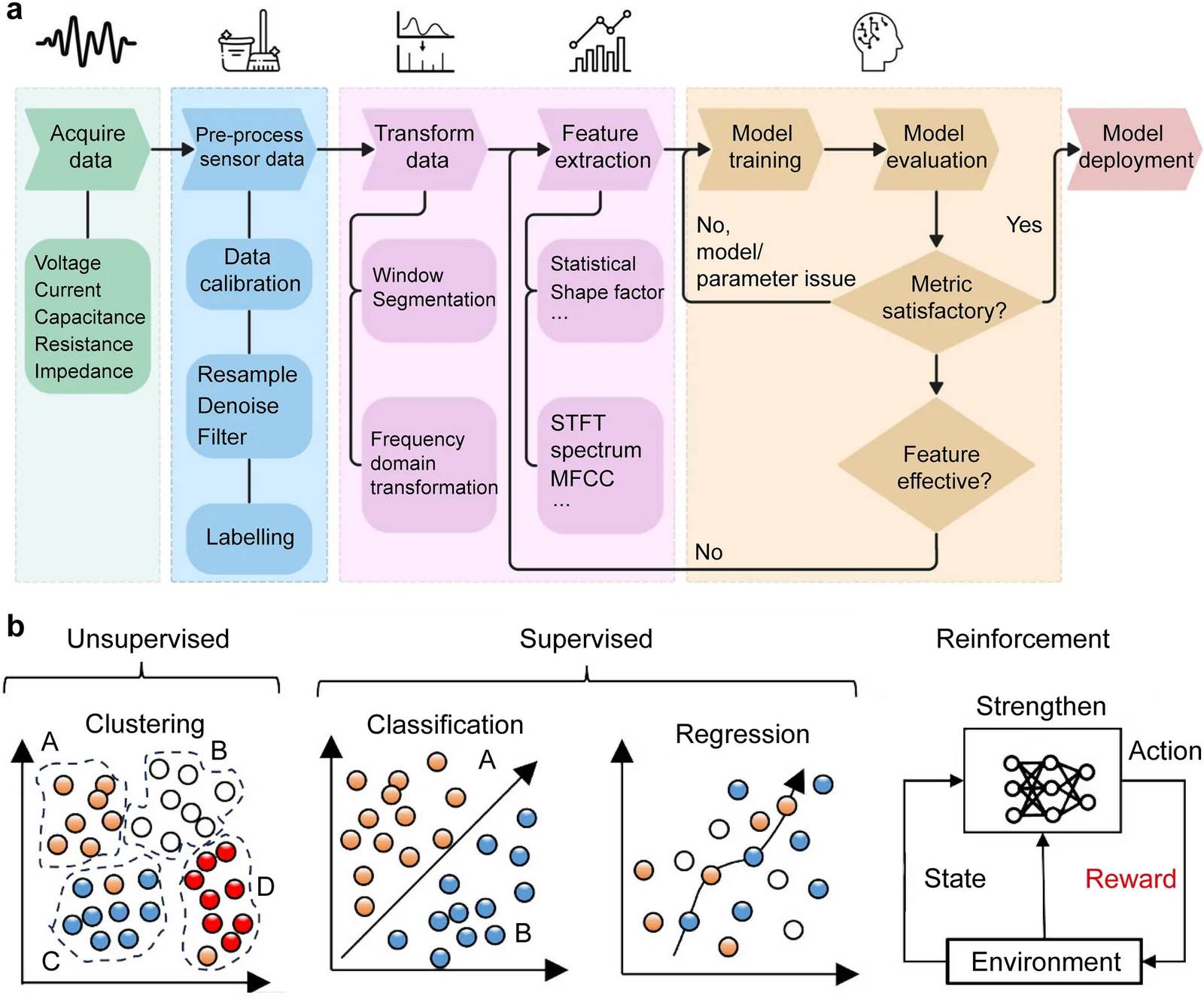
Algorithm-enabled wearable diagnosis. **a** Workflow illustrating the end-to-end data processing and algorithm implementation pipeline for wearable diagnostic systems. Reproduced from Ref. [183] with permission, copyright 2024, American Chemical Society. **b** General classification of ML algorithms and models applicable to personalized health using wearable biosensors, including unsupervised learning, supervised learning, and reinforcement learning. Reproduced from Ref. [186] with permission, copyright 2025, Wiley

Recent advances in ML and deep learning (DL) have enabled wearable diagnostic systems to move beyond passive data logging toward a tightly coupled “sense–interpret–respond” paradigm, in which physiological and biochemical readouts are converted into disease-relevant, actionable insights for end users. By extracting complex response patterns, resolving weak temporal trends, and flagging anomalous signatures from large-scale datasets, these algorithms are reshaping biochemical sensing from signal acquisition to data-driven interpretation and decision support (Fig. 4a) [183]. Leveraging these algorithmic advances, wearable platforms are progressively acquiring capabilities spanning from data processing and quantitative analysis to real-time decision-making and adaptive sensing [22, 28, 184, 185]. Consequently, artificial intelligence (AI)-integrated wearable sensors are emerging as a cost-effective and high-efficiency technological pathway toward personalized health management, addressing both interindividual variability and dynamic health monitoring demands [7].

From a computational perspective, multimodal signals acquired by the sensor front-end are converted into processable digital data by the electronic devices, subsequently entering a well-defined computational pipeline comprising data preprocessing, feature engineering or representation learning, model inference, and result visualization with interactive presentation [186].

ML approaches are commonly categorized into unsupervised, supervised, and reinforcement learning (Fig. 4b). Unsupervised learning operates without manual annotation and can uncover latent patterns, structures, and correlations within input data, thereby serving as an auxiliary information source for downstream decision layers. It is therefore widely applied to pattern recognition, dimensionality reduction, clustering, association rule mining, and feature extraction. In wearable sensing, where physiological and biochemical signals are often high-volume, non-stationary, and affected by motion artifacts, environmental fluctuations, sensor drift, and inter-user variability, unsupervised learning can help extract information-rich representations, reduce redundant dimensions, identify corrupted signal segments, and improve data quality and interpretability [187].

Supervised learning, trained on labeled datasets to learn explicit mappings from input variables to target output [188], plays a pivotal role in disease classification, anomaly detection, and regression estimation of physiological parameters such as respiratory rate, blood pressure, and blood glucose [189–192]. Specifically, classification focuses on state discrimination—such as screening, risk stratification, and abnormality detection—whereas regression supports continuous, real-time quantitative prediction and trend tracking of physiological variables. In wearable applications, however, supervised models must remain robust to missing values, imbalanced labels, limited training cohorts, sensor-to-sensor variability, baseline drift and distribution shifts across users, physiological states, and deployment environments. These requirements highlight the importance of representative datasets, cross-subject validation, adaptive calibration, and robustness testing under realistic operating conditions.

Reinforcement learning learns through repeated interaction with an environment, optimizing a policy based on designed feedback or reward signals rather than conventional labels [193]. This paradigm is particularly attractive for wearable closed-loop systems that couple sensing with therapeutic intervention, as it can dynamically adjust control strategies in response to real-time sensor readouts and user feedback. For instance, in closed-loop diabetes management, the system can autonomously compute and optimize insulin delivery dosing based on continuous glucose monitoring signals, seeking an optimal balance between glycemic control objectives and safety constraints [194]. Despite its potential advantages in high-dimensional, time-varying decision problems and in providing probabilistic error bounds for decision tasks, reinforcement learning remains constrained in clinical applications by data-intensive training requirements, delayed reward signals, and stringent safety and ethical considerations inherent to closed-loop clinical intervention.

Although ML-enhanced biosensing technologies have laid a strong foundation for real-time health monitoring, clinical diagnosis, and decision support, several challenges remain. These challenges are particularly evident in data-related issues, including missing values, noisy signals, and imbalanced datasets, all of which can compromise model accuracy [185]. In practical deployment scenarios, differences in sensor configuration, environmental conditions, hardware constraints, and time-dependent baseline drift can further weaken model generalizability. These problems are amplified in wearable platforms, where device miniaturization, limited sampling frequency, mechanical mismatch, sensor-to-sensor variability, and interference from complex matrices such as sweat, saliva, and blood all affect the reliability of data acquisition [195–197]. Consequently, models trained on narrowly defined or laboratory-controlled datasets may not transfer effectively across users, physiological states or deployment environments. In parallel, the limited interpretability of DL models, together with concerns related to data privacy, algorithmic transparency, ethical governance, and long-term adaptability, continues to hinder the translation of AI-enabled biosensors into trustworthy diagnostic systems [7, 198, 199]. Fully realizing the potential of intelligent algorithms therefore requires stable and reproducible data acquisition, as well as high-quality sensor front-end design. In this context, system-level engineering optimization, particularly in sensor fabrication and system integration, becomes a decisive factor in determining the reliability and trustworthiness of wearable diagnostic platforms.

## Advanced Manufacturing and Integration Technologies

During the transition from traditional, centralized laboratory-based monitoring technology to user-centric wearable technologies, researchers are encouraged to develop new fabrication techniques that can keep pace with this shift and meet the intricate and multifaceted requirements of wearable diagnostic systems, including mechanical compliance, and high-level functional integration. Advanced manufacturing, encompassing both additive and subtractive manufacturing, allows precise construction of fundamental electronic components on diverse substrates, such as sensors, electrodes, interconnects, and energy modules, thereby achieving fine-grained device-level processing and performance regulation. Building upon these processes, integration technologies further consolidate dissimilar material systems, heterogeneous device types, and diverse functional modules at the system level, facilitating the evolution from discrete devices to fully integrated electronic platforms. Taken together, advanced manufacturing and integration act synergistically, providing essential technological foundations for achieving high device density, multifunctionality, and long-term reliable operation in wearable diagnostic systems.

### Advanced Manufacturing for Material Deposition and Patterning

**Fig. 5:**
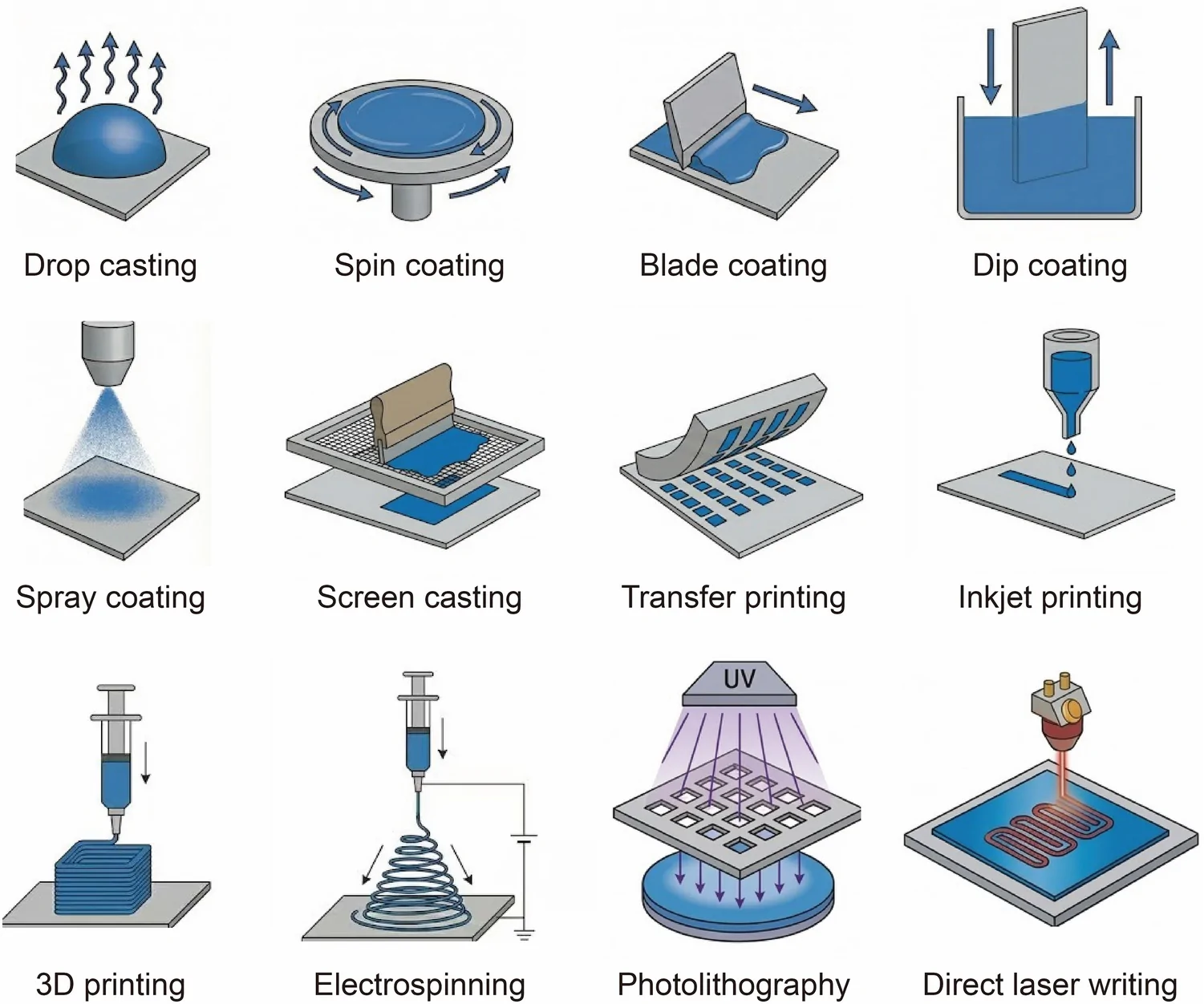
Strategies for fabrication of wearable diagnostic systems using additive and subtractive manufacturing

In wearable diagnostic systems, additive and/or subtractive manufacturing allows control over the distribution of sensitive materials, conductors, and integrated flexible electronic components. It includes additive manufacturing techniques, such as drop casting, spin coating, blade coating, dip coating, spray coating, transfer printing, inkjet printing, three-dimensional (3D) printing, and electrospinning, as well as subtractive manufacturing techniques, such as photolithography and direct laser writing (Fig. 5).

#### Coating Techniques

Coating techniques, such as drop casting, spin coating, blade coating, and dip coating, are one of the most widely used classes of gentle processing methods in bioelectronics and wearable medical devices, as shown in Fig. 5. They are suitable for constructing sensitive material layers, biointerfacial layers, and flexible functional films.

**Fig. 6:**
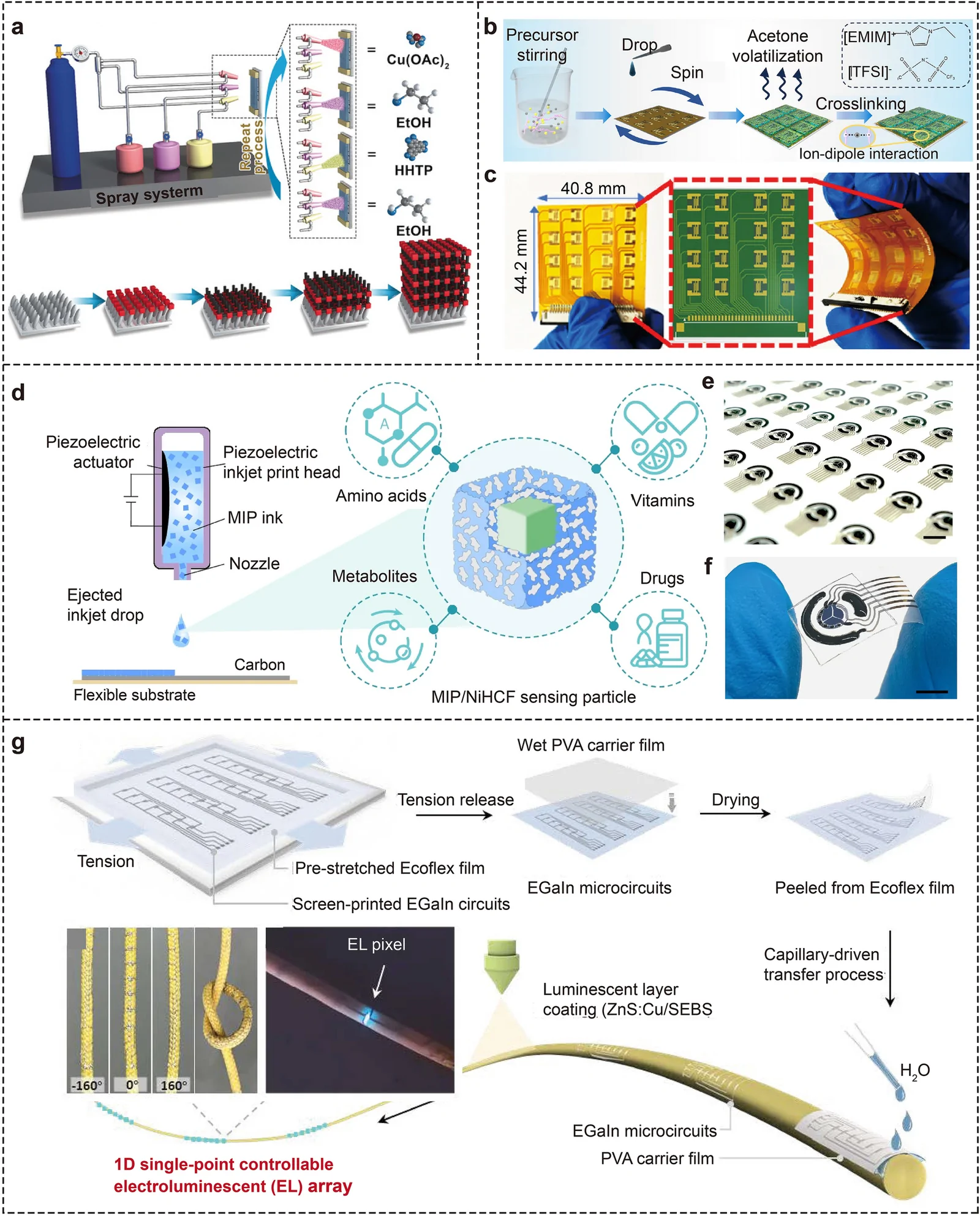
**a** Illustration of the preparation of MOF thin-film gas sensors using the spray method. Reproduced from Ref. [206] with permission, copyright 2017, Wiley. **b** Scheme illustrating the one-time scalable fabrication process of ionogel-based humidity sensor array. **c** Comparison of the standard deviation between the sensor array and sensors prepared separately. Reproduced from Ref. [207] with permission, copyright 2025, Wiley. **d** Schematic of inkjet-printable core–shell cubic NPs for the mass production of highly stable sensors. **e** Optical image of flexible sensor arrays mass-produced via inkjet printing of MIP/NiHCF NPs. Scale bar, 5 mm. **f** Photograph of a wearable patch. Scale bar, 5 mm. Reproduced from Ref. [215] with permission, copyright 2025, Springer Nature. **g** Illustration of the fabrication process for electroluminescent (EL) fiber devices. Reproduced from Ref. [217] with permission, copyright 2026, Springer Nature

Various biochemical sensors are frequently fabricated via drop casting, particularly for rapid prototyping and proof-of-concept demonstrations in laboratory settings [200, 201]. Spin coating, spray coating, and dip coating have been used to modify sensing material for biomedical sensors to address these challenges [202–205]. For instance, Yao et al. reported a spray-based layer-by-layer liquid-phase epitaxial method to fabricate conductive metal–organic framework (MOF) nanofilms with tunable thickness for room-temperature chemiresistive sensing (Fig. 6a) [206]. And Zhang et al. employed a scalable and modified spin-coating approach to fabricate an ionogel film-based flexible humidity sensing array with fast response characteristics, allowing precise detection and spatial mapping of humidity distribution (Fig. 6 b, c) [207].

At the same time, in wearable diagnostic systems, functional thin films are typically required as sensor substrates, interfacial modification layers, or encapsulation films, to ensure mechanical compliance and stable device performance. Spin coating and blade coating of polymer materials provides an effective approach to obtain uniform films with precisely controlled thickness [208, 209]. Recently, skin-like drift-free biosensors based on stretchable diode-connected OFETs have been reported. In this device architecture, spray coating was employed to fabricate the source, drain, and gate electrodes, while spin coating was used to form the styrene–ethylene–butylene–styrene (SEBS) and polydimethylsiloxane (PDMS) thin-film layers. The combination of these deposition strategies enabled both mechanical compliance and stable electrical performance [210].

Overall, coating techniques show different translational potentials for wearable diagnostic systems. Drop casting requires almost no specialized equipment and has very low cost, making it suitable for material screening and proof-of-concept demonstrations. However, its manual operation, poor film uniformity, weak thickness control, and batch-to-batch variation limit production yield and device robustness. Spin coating enables uniform thin films with controllable thickness and is useful for dielectric layers, ISMs, polymer substrates, and encapsulation films in planar wearable devices, but its material waste and poor compatibility with non-planar or textile substrates increases the effective unit cost. Blade coating is more suitable for scalable fabrication because it can be adapted to continuous coating and R2R processes for large-area sensing films, hydrogel layers, adhesive interfaces, and encapsulation coatings, although film quality strongly depends on solution viscosity, blade gap, coating speed, drying behavior, and substrate wettability. Spray coating allows large-area and conformal deposition on curved, porous, or stretchable substrates, making it useful for stretchable electrodes, conductive networks, gas-sensing layers, and electronic-skin interfaces. However, conventional spraying may cause solution waste due to overspray; therefore, ultrasonic atomization or assisted spraying strategies are often used to improve material utilization and coating-thickness control. Dip coating is suitable for batch and conformal modification of fibers, textiles, MNs, porous membranes, and 3D structures, but reproducible thickness, spatial patterning, and high production yield remain challenging.

#### Printing Techniques

Printing techniques, including inkjet printing, screen printing, gravure and flexographic printing, and aerosol jet printing, allow the direct formation of conductive interconnects or functional sensing modules by precisely depositing functional inks onto various substrates, including paper, metal, glass, rubber, and polymeric films, making them particularly attractive for wearable diagnostic systems [211–215].

Different printing methods show distinct translational advantages. Inkjet printing requires moderate equipment investment and offers high material utilization, making it suitable for customized and small-batch wearable sensors, such as electrochemical biosensors, sweat sensors, gas sensors, and flexible electrodes. For example, noninvasive wearable and implantable biosensors were fabricated using a streamlined inkjet printing technique for biomarker monitoring (Fig. 6d–f) [215]. By depositing a core–shell nanoparticle (NP) ink with built-in dual functionality, customizable target recognition, and stable electrochemical transduction, this method successfully eliminates the need for labor-intensive manual modifications. It serves not only as material deposition tools, but also as important manufacturing platforms for integrating sensing, signal transmission, and functional actuation modules in wearable healthcare systems. For example, Choi et al. recently developed a strategy based on inkjet printing to integrate various functional organic and inorganic nanomaterials onto flexible polymer substrates. Using this approach, key components, including artificial synapses, memristors, and resistive elements, were successfully fabricated, enabling the integration of sensing and analog signal processing within a fully printed platform [216]. However, its scalability is limited by nozzle clogging, ink rheology, coffee-ring effects, printing resolution, and batch-to-batch reproducibility.

Screen printing requires relatively simple equipment and provides high throughput with low unit cost after scale-up, making it more suitable for disposable diagnostic patches, printed electrodes, glucose/lactate sensors, and point-of-care biosensors. However, it is predominantly limited to planar substrates. To overcome the geometric incompatibility between planar fabrication processes and curved surfaces, a shrinkage-transfer-assisted printing strategy was proposed (Fig. 6g). By shrinking eutectic gallium–indium (EGaIn) fluidic circuits and transferring them onto curved fiber surfaces, this approach effectively bridges two-dimensional planar circuit fabrication with one-dimensional fiber device construction [217].

Gravure and flexographic printing require higher initial investment in R2R systems and customized rollers or plates, but their high production speed can substantially reduce the cost per unit during large-volume production. Therefore, they are particularly suitable for scalable fabrication of large-area flexible circuits, packaging layers, disposable biosensor patches, and printed interconnects for wearable diagnostic and systems. These features make them promise commercial production of low-cost sweat-monitoring patches, wound-care sensors, electrophysiological monitoring electrodes, and point-of-care wearable diagnostic devices. Aerosol jet printing provides high resolution, non-contact deposition, good material utilization, and compatibility with curved, flexible, or non-planar substrates, making it suitable for microelectrodes, stretchable interconnects, conformal sensing interfaces, and heterogeneous wearable diagnostic systems. For example, Herbeter et al. fabricated fully flexible sensor by aerosol jet printing the PI supporting substrate, silver NP conductive layer, and PDMS, enabling fully implantable, wireless, battery-free vascular electronics for multiplexed hemodynamic sensing [218]. However, its relatively expensive aerosol generation and focusing systems, low throughput, and strict ink/process requirements increase manufacturing cost and limit its scalability for low-cost mass production.

#### 3D Printing Techniques

3D printing, as a representative digital additive manufacturing technology, constructs structures through layer-by-layer deposition. It enables the fabrication of customized mechanical supporting for wearable diagnostic systems, as well as the production of biomedical sensors through the integration of biocompatible or functional materials.

**Fig. 7:**
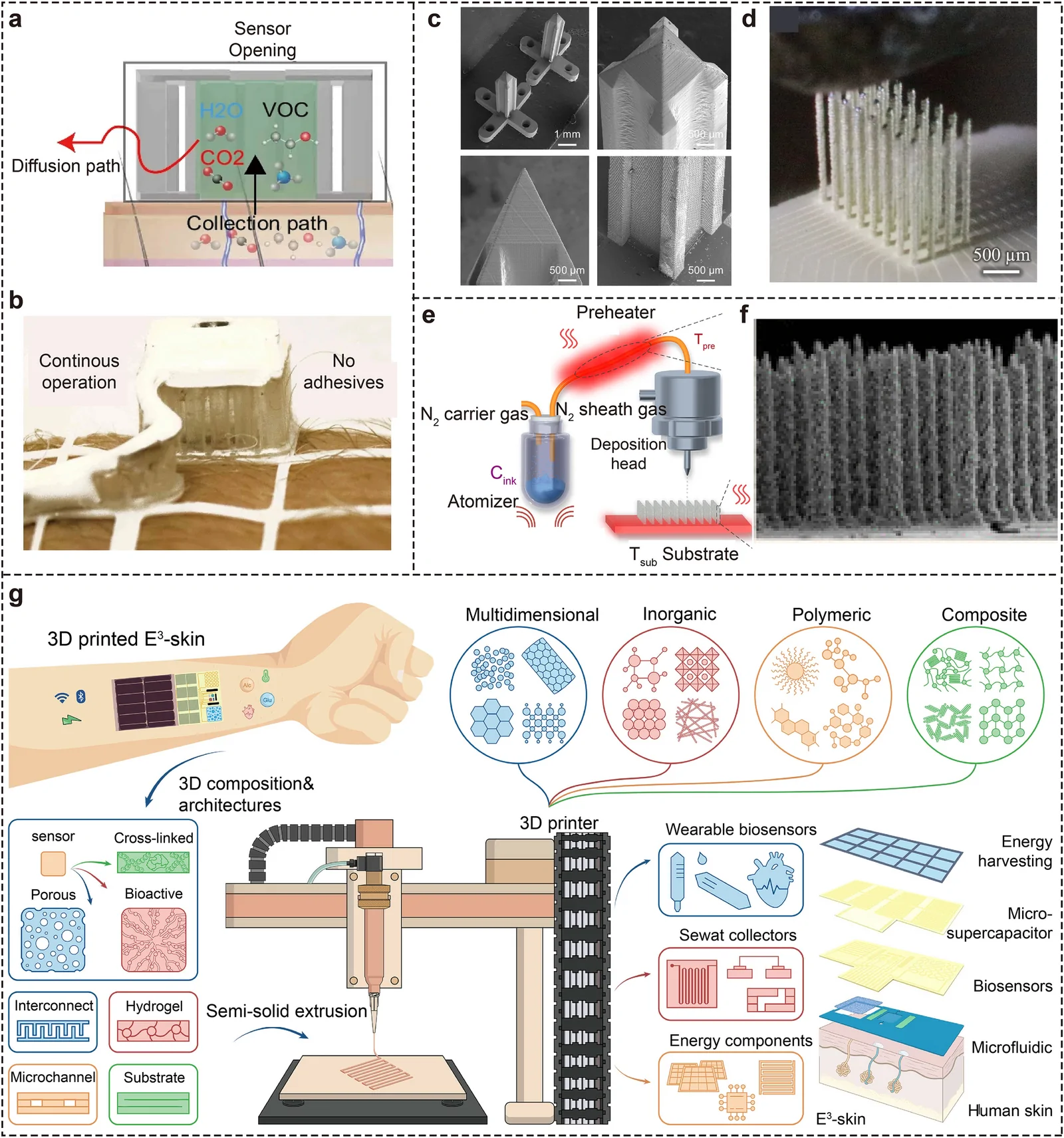
**a-b** Illustration and photographic image of the realized 3D-printed cavity with electronics installed. Reproduced from Ref. [220] with permission, copyright 2025, Springer Nature. **c** Scanning electron microscopy (SEM) images of 3D-printed MN. Reproduced from Ref. [219] with permission, copyright 2025, Springer Nature. **d** Image of a 32-channel probe. Reproduced from Ref. [229] with permission, copyright 2022, American Association for the Advancement of Science. **e** Illustration of temperature-induced self-assembly aerosol jet 3D printing for multiscale structures. **f** SEM image of micropillar structure. Reproduced from Ref. [230] with permission, copyright 2025, American Chemical Society. **g** Schematic illustration of the SSE-based 3D printing that features highly customizable inks based on versatile materials. Reproduced from Ref. [231] with permission, copyright 2023, American Association for the Advancement of Science

In recent years, mechanical support structures for wearable devices, such as housing, frames, and protective architectures, have been developed through 3D printing techniques [219]. Clausen et al. printed a cavity structure with integrated micropillars enabling diffusion-based gas exchange with the ambient environment. By coupling this architecture with sensors, the 3D-printed platform allows real-time monitoring of sweat rate, volatile organic compounds (VOCs), and CO_2_ (Fig. 7a, b) [220]. With the rapid improvement in printing resolution, 3D printing technologies have been widely used to fabricate high-precision molds, which can subsequently enable the fabrication of microstructured biomedical devices via replica molding. Representative examples include microfluidic chips [221], MN [27], and various biomedical sensors, such as ultrasonic metagel-based sensors [222].

Moreover, owing to their compatibility with a wide range of biocompatible polymers, elastomers, hydrogels, and composite materials, 3D printing technologies have been widely employed to directly fabricate structurally tunable micro- and nanostructures for wearable devices [223–225]. Diverse MN architectures—including pyramidal, faceted, porous, and barbed structures—can be fabricated via 3D printing for applications such as ISF extraction, biosensing, and drug delivery [226–228]. For example, Yang et al. employed 3D printing technique to fabricate a dual-sensor MN with a “four faces” structure for continuous measurement of glucose and metformin concentrations in skin ISF, in Fig. 7c [219]. In addition, 3D printing enables the fabrication of electrode arrays with high spatial resolution. Saleh et al. developed customizable and mechanically robust 3D multi-electrode devices using a 3D NP printing approach (Fig. 7d) [229]. Similarly, Zhou et al. reported a temperature-induced self-assembly aerosol jet printing strategy to precisely construct multiscale micropillar gate electrodes with tunable nanostructures for ultrasensitive dopamine detection (Fig. 7e, f) [230].

Importantly, the multi-material compatibility of 3D printing further enables the integration of multiple functional modules into integrated multimodal systems. As shown in Fig. 7g, Gao’s group employed a universal semi-solid extrusion (SSE)-based 3D printing strategy to pattern a range of customized functional materials, including carbon nanomaterials, MXene, hydrogels, and polymers, into multimodal sensors, microfluidic channels, and micro-supercapacitors. Without the need for additional processing steps such as lithography or electroplating, these components were directly integrated into a fully 3D-printed epifluidic elastic electronic skin(e^3^-skin) [231].

Although advanced 3D printing techniques can achieve nanoscale structural fabrication, they are often limited to small printing areas, high processing costs, and low throughput, making large-area and scalable manufacturing difficult. Moreover, post-processing steps such as curing, washing, coating, sterilization, and surface modification are often required, which increases manufacturing complexity and cost. Therefore, 3D printing is more suitable for personalized devices, while commercial-scale translation still requires faster printing systems, standardized biocompatible materials, improved resolution, and better long-term mechanical stability.

#### Electrospinning Techniques

**Fig. 8:**
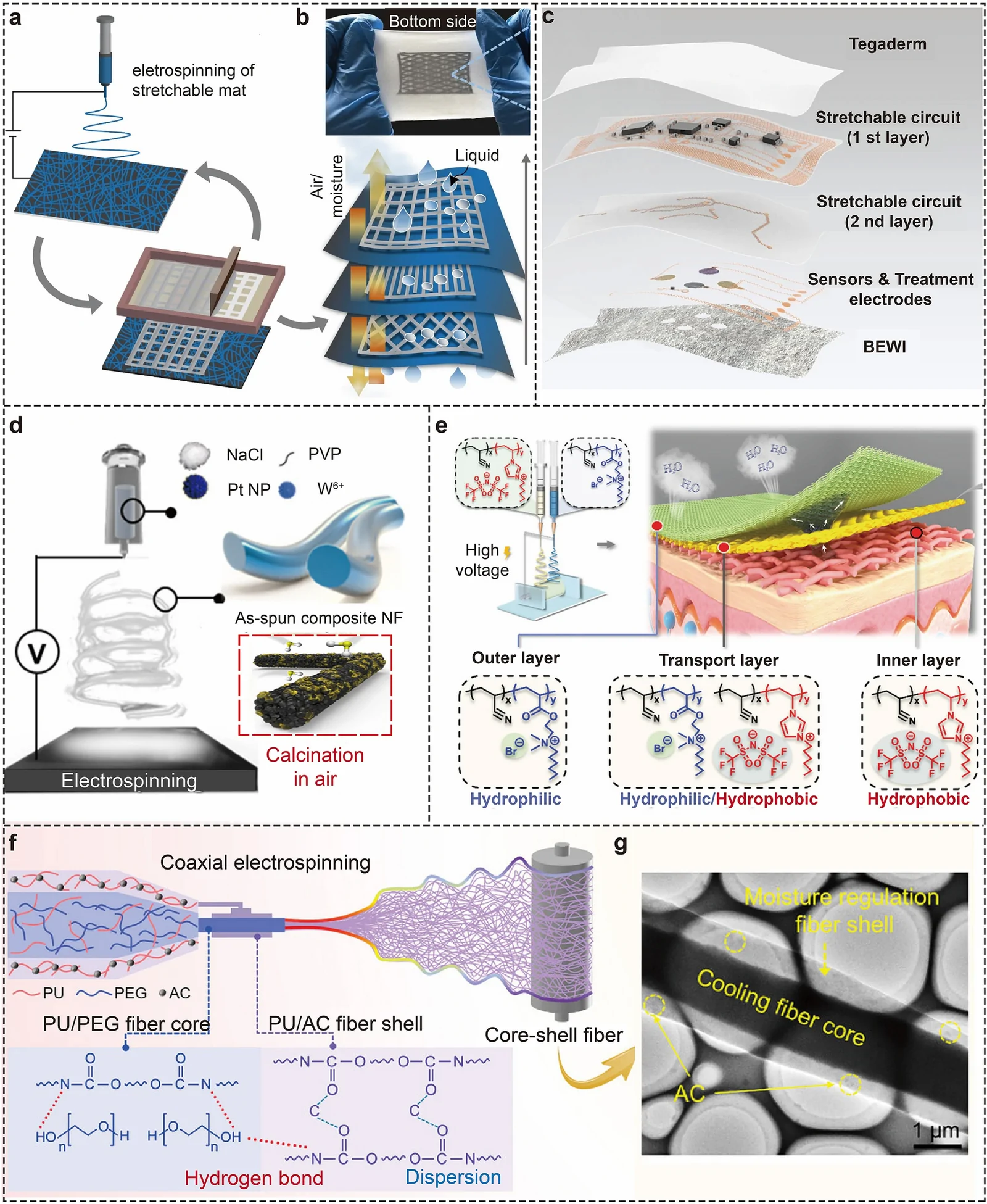
**a** Schematic illustration showing the fabrication of the vertically stacked monolithic stretchable mat by alternating electrospinning and stencil printing. **b** Digital images of a three-layer monolithic stretchable device and the electrode structures in each layer. Reproduced from Ref. [236] with permission, copyright 2021, Springer Nature. **c** Exploded-view illustration of multifunctional electronics. Reproduced from Ref. [232] with permission, copyright 2025, Springer Nature. **d** Schematic illustrations of fabrication gas sensor based on incorporation precursor solution and electrospinning process. Reproduced from Ref. [233] with permission, copyright 2021, American Chemical Society. **e** Schematic depicting the fabrication and application of PIL membrane. Reproduced from Ref. [162] with permission, copyright 2022, Wiley. **f** Schematic illustration of coaxial electrospinning for the fabrication of core–shell fibers. **g** Transmission electron microscopy image for core–shell fiber. Reproduced from Ref. [235] with permission, copyright 2026, Wiley

Electrospinning is a typical field-driven additive manufacturing technique that enables the continuous fabrication of ultrafine fibers with diameters ranging from the nanometer to micrometer scale. Owing to their high specific surface area, excellent mechanical compliance, and good breathability, electrospun nanofiber membranes are widely used as breathable supporting substrates, as well as conductive or insulating substrates. Conductive materials, sensing materials, bioactive materials, and electronic components can be further integrated onto these membranes to construct electrode structures or various sensing devices (Fig. 8a–c) [232].

However, in the most basic electrospinning systems, the fibrous membranes are typically composed of a single polymer, and their function is mainly limited to providing a breathable structural scaffold. Such membranes often cannot simultaneously satisfy the requirements of conductivity, sensitivity, and structural stability in wearable diagnostic systems. Generally, functional nanomaterials can be incorporated into the spinning solution and composited with polymers to directly construct multifunctional fibers, like conductive or sensing fiber networks. For example, Shin et al. introduced facile co-functionalization of WO₃ nanofibers with an alkali metal (Na) and a noble metal (Pt) catalyst was achieved by simply adding NaCl and Pt NPs into the precursor solution, followed by an electrospinning process, resulting in a surface activity-tuned gas sensor (Fig. 8d) [233]. By tuning the composition of the precursor solution followed by electrospinning, functional fibers can be readily fabricated through a relatively simple process. However, the distribution of different components within these fibers is typically random, making precise control over the spatial distribution of materials and interfacial structures challenging [234]. To overcome this limitation, advanced multi-stream electrospinning techniques have been developed, including multi-axial electrospinning and coaxial electrospinning. Multi-axial electrospinning facilitates the construction of multilayer fibrous structures, whereas coaxial electrospinning enables the formation of core–shell architectures within individual fibers. For example, Zheng et al. by modifying the cation and anion of a poly(ionic liquid) (PIL) and employing a dual-nozzle electrospinning strategy, created a PIL-based multilayer nanofiber membrane (PIL membrane) electronic skin (e-skin) with a dual gradient (Fig. 8e) [162]. Meng et al. developed a thermal- and moisture-regulating textile via coaxial electrospinning, featuring a rationally designed core–shell micro/nanofiber structure with a moisture-regulating polymer sheath and a thermoresponsive polymer core (Fig. 8f, g) [235].

However, the large-scale fabrication of electrospun membranes remains challenging because conventional electrospinning has a low deposition rate and often requires long processing times to obtain membranes with sufficient thickness and mechanical robustness. The process is also highly sensitive to solution properties and environmental conditions, including viscosity, solvent evaporation, voltage, flow rate, humidity, and temperature, which can cause nozzle clogging, unstable jet formation, nonuniform fiber morphology, uneven membrane thickness, and poor batch-to-batch reproducibility. These issues become more pronounced during long-duration or large-area production, especially when functional NPs, conductive fillers, biomolecules, or drugs are incorporated. Therefore, scalable electrospinning for wearable diagnostic systems requires multi-needle, needleless, or R2R systems, together with automated process control, solvent management, and inline quality inspection to improve throughput, reduce cost per unit, and ensure reproducible membrane quality.

#### Photolithography Techniques

Photolithography refers to a fine microfabrication technique in which a photoresist undergoes chemical changes upon exposure to light of specific wavelengths or particle beams. Through sequential processes such as exposure, development, etching, and patterns designed on a photomask are transferred onto a substrate with high fidelity. It is characterized by high resolution, precise dimensional controllability, and excellent reproducibility and uniformity and used for fabricating flexible and wearable devices which can be broadly categorized into two main approaches.

**Fig. 9:**
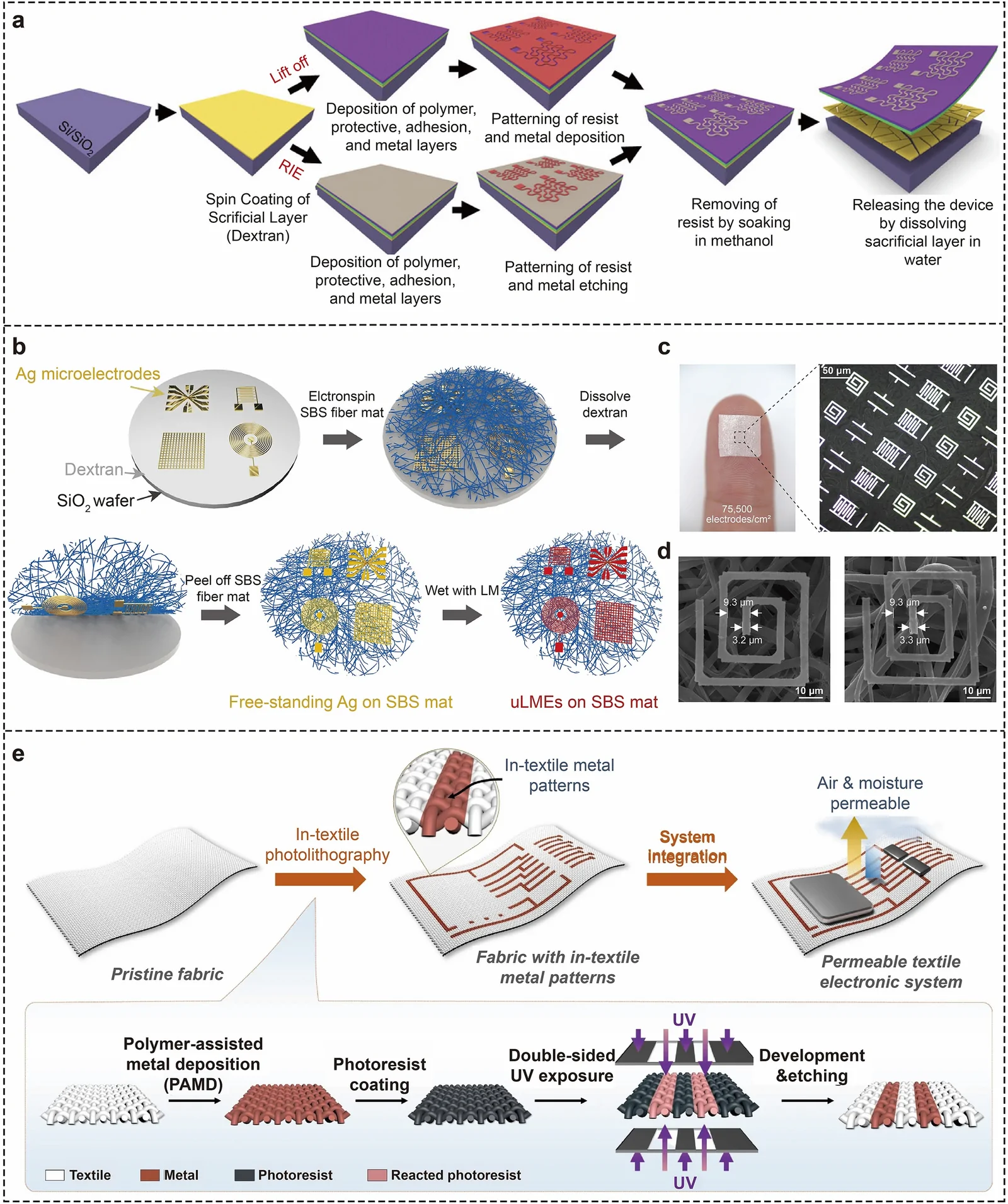
**a** Schematic illustration of the photolithography-based procedure through lift-off and reactive ion etching (RIE) methods. Reproduced from Ref. [239] with permission, copyright 2023, Wiley. **b** Schematic illustration of the fabrication process of μLMEs through photolithography techniques. **c** Digital images of high-density μLMEs. **d** Scanning electron microscopy images showing an electrode on the SBS fiber mat before and after the selective wetting with liquid metal (LM). Reproduced from Ref. [238] with permission, copyright 2023, American Association for the Advancement of Science. **e** Schematic illustration showing the development of textile electronics by in-textile photolithography technology. Reproduced from Ref. [240] with permission, copyright 2024, Springer Nature

The first approach is based on conventional silicon-based photolithography, in which an etchable or dissolvable sacrificial layer is first introduced onto a silicon wafer. After device fabrication, the sacrificial layer is removed to release the entire device or transfer it onto flexible or stretchable substrates. This strategy is technologically mature and is commonly employed for the fabrication of complex flexible electronic systems [208, 209, 237, 238]. For example, Bathaei et al. employed photolithography-based microfabrication to fabricate flexible and stretchable sensors on substrates such as polylactic acid (PLA) and polyglycerol sebacate (PGS) (Fig. 9a) [239]. Besides integration on dense thin films, sensors sometimes need to be constructed on porous or breathable substrates, particularly for wearable applications that demand air permeability, flexibility, and long-term comfort. To address this, Zhuang et al. reported wafer-scale patterning of permeable and stretchable liquid metal microelectrodes (μLMEs) on fibrous substrates based on photolithography, achieving resolutions down to 2 μm and an ultrahigh patterning density exceeding 75,000 electrodes cm^−2^ (Fig. 9b–d) [238]. High-resolution electrodes are vital for imaging, such as biomarker mapping in wound diagnostics, and represent a critical step toward precision spatial monitoring.

The second approach involves direct photolithographic patterning on elastic electronic materials themselves, enabling high-precision pattern definition without the need for device release or transfer processes [240–242]. Wang et al. demonstrated the use of in-textile photolithography to fabricate an integrated, multiplexed biosensing headband for wireless sweat sensing (Fig. 9e) [240]. Due to inherent surface roughness of elastomers that limits high-density lithography, Peng’s group combined plasma surface smoothing with parylene deposition to construct a hard–soft heterogeneous interface to reduce surface roughness and improve chemical stability. By further adopting a multilayer helical architecture, the integration density reaches 100,000 transistors per centimeter on the fiber [64].

In general, photolithography offers high resolution, excellent dimensional controllability, device uniformity, and mature semiconductor compatibility, making it highly suitable for high-density electrode arrays, multiplexed biosensors, transistor arrays, microelectronic circuits, and spatially resolved wearable diagnostic systems. These advantages are particularly important for applications requiring precise signal acquisition, wound biomarker mapping, electrophysiological recording, and intelligent wearable sensing platforms. However, photolithography requires expensive cleanroom facilities, photomasks, exposure and etching equipment, and multiple chemical processing steps, leading to high initial investment and fabrication cost. Moreover, its conventional planar wafer-based process is not naturally compatible with soft, stretchable, porous, or textile substrates, often requiring sacrificial-layer release, transfer printing, or additional packaging steps. These processes increase fabrication complexity and may reduce production yield. Therefore, photolithography is better suited for high-value, miniaturized, and high-performance wearable diagnostic modules rather than low-cost disposable patches. For clinical and commercial translation, photolithography is most effective when combined with transfer printing, soft encapsulation, printing, or coating technologies to integrate high-performance microfabricated components with mechanically compliant wearable diagnostic systems.

#### Direct Laser Writing Techniques

**Fig. 10:**
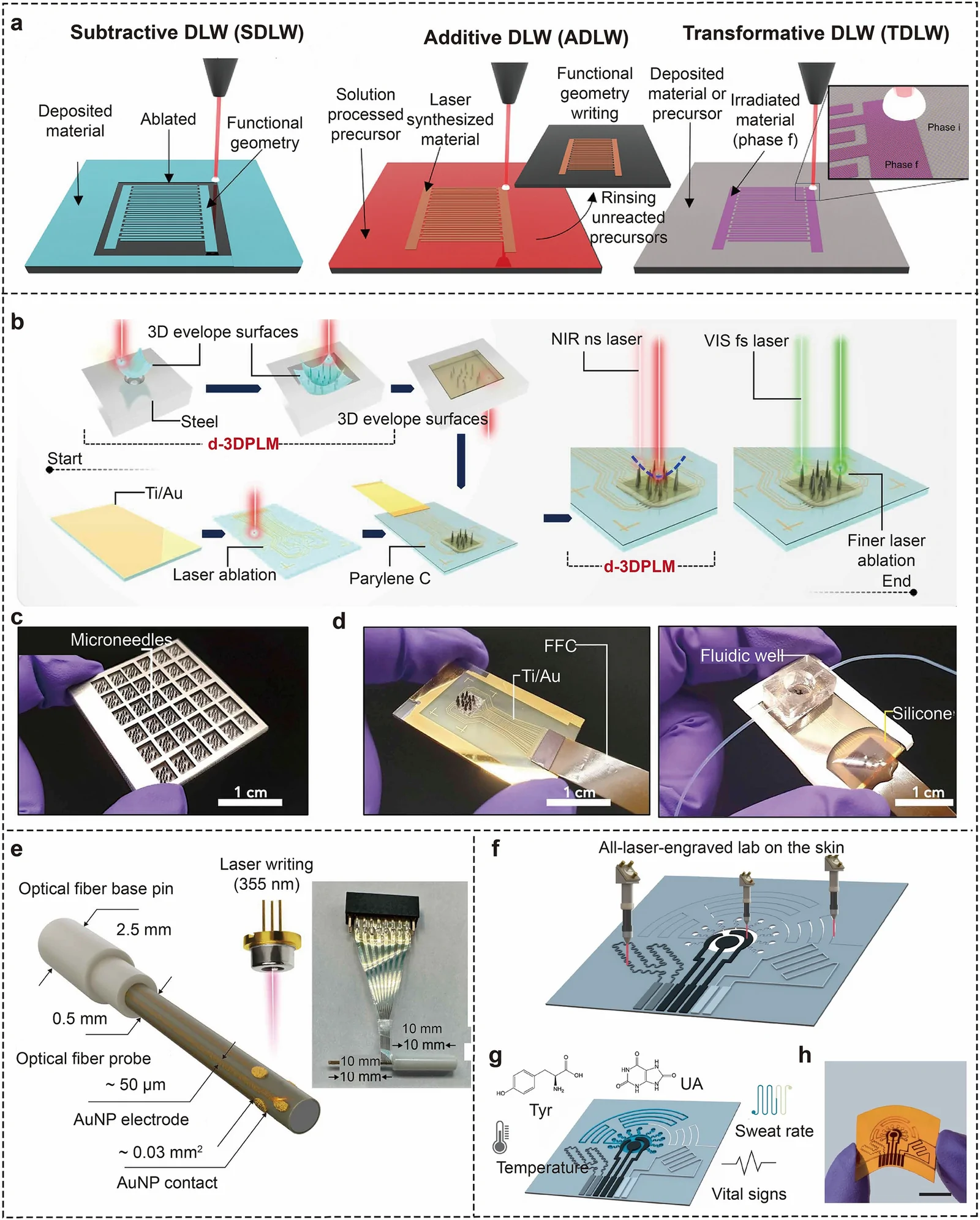
**a** Schematic of SDLW, ADLW, and TDLW. Reproduced from Ref. [243] with permission, copyright 2024, Wiley. **b** Schematic of the main steps of the fabrication process for the 3D MEAs. **c-d** Real photograph of the 3D MEAs with assembly of fluidic well. Reproduced from Ref. [248] with permission, copyright 2025, American Association for the Advancement of Science. **e** Illustration of the laser-based in situ reduction process used to fabricate the embedded AuNP electrodes. Insect: optical image of the AuNP-fiber probe with an FPC connector. Reproduced from Ref. [253] with permission, copyright 2025, American Association for the Advancement of Science. **f** Sensors with entirely laser-engraved components: the microfluidic module and the LEG-based chemical and physical sensors. **g** Multiple functions of the sensors: ultrasensitive sweat uric acid (UA) and tyrosine (Tyr) detection, sweat rate estimation, temperature sensing, and vital-sign monitoring. **h** Photographic image of a flexible laboratory-on-skin patch. Scale bar, 1 cm. Reproduced from Ref. [255] with permission, copyright 2020, Springer Nature

Direct laser writing (DLW) focuses laser beam to perform point-by-point or line-by-line scanning on or within materials. Compared with photolithography, DLW avoids photomasks, photoresists, development, and complex etching steps, thereby reducing process complexity and enabling rapid prototyping and customized fabrication. According to the underlying material–laser interaction mechanisms, DLW can be broadly classified into subtractive direct laser writing (SDLW), additive direct laser writing (ADLW), and transformative direct laser writing (TDLW) (Fig. 10a) [243].

In wearable electronics, SDLW is widely employed not only for material patterning—such as the fabrication of flexible circuits and high-density electrode arrays [244–246]—but also for structural engineering, including the formation of through-holes, microchannels, and various mask structures [238, 247]. For example, Mariello et al. introduced dynamically autofocused 3D pulsed laser micromachining (d-3DPLM) using a nanosecond pulsed near-infrared laser, enabling the customizable fabrication of 3D bioelectronic architectures such as 3D multi-electrode arrays (MEAs) devices (Fig. 10b–d) [248].

ADLW is characterized by the simultaneous synthesis and patterning of materials, typically based on inks or solution systems containing reactive precursors. And TDLW is based on direct laser irradiation of preexisting materials, where localized energy input induces chemical or structural transformations, such as phase transitions, carbonization, reduction, or cross-linking, without substantial material removal or the introduction of external precursors. This process neither adds nor removes material; instead, functional patterning is achieved [243]. Through laser sintering, laser-induced reduction, oxidation, metallization, carbonization, or graphitization, ADLW and TDLW can form the patterns directly on substrates, which can be readily integrated into electrodes and sensors [249–252]. For instance, Zhang et al. used ADLW method to fabricate electrode arrays via in situ reduction of gold NPs (AuNPs) onto the curved surface of optical fibers (Fig. 10e) [253]. Ding et al. employed a TDLW strategy to fabricate laser-induced graphene (LIG) electrode arrays on flexible polyimide substrates for an electronic nose system, enabling the recognition of multiple diseases through the detection of urinary volatile compounds [254]. And Gao’s group designed the entirely laser-engraved sensors for multimodal monitoring. CO_2_ laser was used to fabricate all key components of the sensors: the laser-engraved graphene (LEG)-based chemical sensors, the LEG-based physical sensors, and the multi-inlet microfluidic module (Fig. 10f–h) [255]. Although DLW provides excellent design flexibility, its patterning resolution and conductivity can vary depending on the laser process and the converted material, especially for LIG or reduced metal patterns.

Therefore, future translation should focus on improving laser-processing speed, automated patterning control, substrate compatibility, inline quality inspection, and hybrid integration with printing, coating, or transfer technologies.

## Summary

High-performance wearable diagnostic systems are unlikely to be commercialized through a single manufacturing technique. A more realistic route is the integration of multiple advanced fabrication methods in a cost-performance-balanced manufacturing framework. Low-cost and scalable processes, including coating, screen printing, R2R printing, and flexible printed circuit manufacturing, can reduce production cost by enabling large-area fabrication of electrodes, interconnects, and functional sensing layers. In parallel, high-resolution or function-specific processes, such as photolithography, DLW, aerosol jet printing, electrospinning, and 3D printing, can be deployed only where they are needed, for example, to define high-density microelectrodes, localized sensing interfaces, porous skin-contact layers, soft microfluidic channels, or heterogeneous electronic modules. Such selective use of advanced manufacturing methods avoids the excessive cost of applying high-precision processes to the entire device, while still preserving the performance advantages required for sensitive, stable, and multifunctional wearable diagnostics. This hybrid strategy therefore provides a practical route to reconcile low unit cost, high device performance, mechanical compliance, production scalability, and long-term reliability.

### Advanced Integration Technologies

In wearable diagnostic systems, achieving excellent performance of individual functional modules alone is insufficient for practical clinical applications. Beyond high sensing accuracy and multifunctionality, wearable diagnostic systems are also required to maintain long-term operational reliability under complex real-world conditions, including repeated mechanical deformation, sweat or biofluid exposure, temperature and humidity fluctuations, and daily motion artifacts. These challenges can significantly influence the electrical stability, mechanical durability, biointerfacial compatibility, and overall service lifetime of wearable diagnostic devices. Therefore, increasing attention has been devoted not only to enhancing device-level performance, but also to improving system-level reliability and operational stability. Representative long-term reliability challenges and corresponding mitigation strategies are summarized in Table 2, highlighting that successful clinical translation depends on both high-performance sensing capability and robust operation in complex conditions.

**Table 2:** The summary of long-term reliability challenges and corresponding strategies for wearable diagnostic systems

Challenge	Main failure modes	Strategies	References
Biofouling	Protein adsorption, lipid deposition, salt crystallization, bacterial biofilm, wound exudate accumulation	Antifouling coatings, hydrogel interfaces, permselective membranes, replaceable sensing layers, microfluidic refreshment	[19, 22, 120, 129, 256]
Signal drift	Enzyme degradation, mediator leaching, reference-electrode drift, recognition-layer detachment, baseline instability	Stable immobilization, reference channels, ratiometric design, temperature/pH compensation, algorithmic recalibration	[257–260]
Mechanical durability	Bending, stretching, compression, skin friction, adhesive fatigue, interconnect cracking, delamination	Serpentine interconnects, strain-isolation structures, soft-hard interface engineering, stretchable adhesives, encapsulation	[261–263]
Environmental interference	Temperature/humidity fluctuation, ambient light, electromagnetic noise, sweat evaporation, motion artifacts, placement variation	Shielding, optical isolation, auxiliary sensors, motion correction, humidity management, multimodal compensation	[197, 264–266]
Power and wireless stability	Battery depletion, energy-harvesting fluctuation, antenna detuning, body-shadowing, variable readout distance	Low-power circuits, energy buffering, wireless charging, robust antenna design, edge processing	[58, 267–269]

Driven by these requirements, recent advances in advanced manufacturing technologies have enabled the scalable fabrication of high-density and high-performance functional units based on diverse material systems and device architectures, including sensors, processors, memory, and energy modules. Nevertheless, the realization of practical wearable diagnostic systems ultimately relies on the effective integration of these functional units into unified, compact, and mechanically compliant platforms. In this context, manufacturing technologies primarily determine the structural precision, material compatibility, and functional diversity of individual devices, whereas integration strategies govern system-level interconnection, signal coordination, mechanical reliability, and multifunctional operation. Consequently, beyond the fabrication of isolated components, current research has increasingly focused on developing sophisticated integration architectures capable of bridging material innovation with application-oriented system implementation. The following section discusses recent advanced integration technologies for wearable diagnostic systems.

#### Soft-Hard Electrical Interfacing

Most current wearable diagnostic systems consist of flexible modules (e.g., flexible sensors) integrated with rigid modules, forming what is known as flexible hybrid electronics. One of the key technological challenges in flexible electronics is achieving stable and reliable electrical interfacing between soft modules (e.g., sensors) and external circuits or operating microchips. During the integration of flexible modules and rigid modules, interconnection can be achieved using soldering paste, LM-based soldering paste, conductive adhesives, and adhesive films. Because soft-hard junctions concentrate mechanical strain and are frequently exposed to sweat, skin motion, and repeated bending, they are also among the most common failure sites during long-term wearable operation. Therefore, interfacial technologies should be evaluated not only by initial contact resistance, but also by resistance stability, adhesion retention, and fatigue lifetime under cyclic deformation and humid or biofluid-rich conditions.

**Fig. 11:**
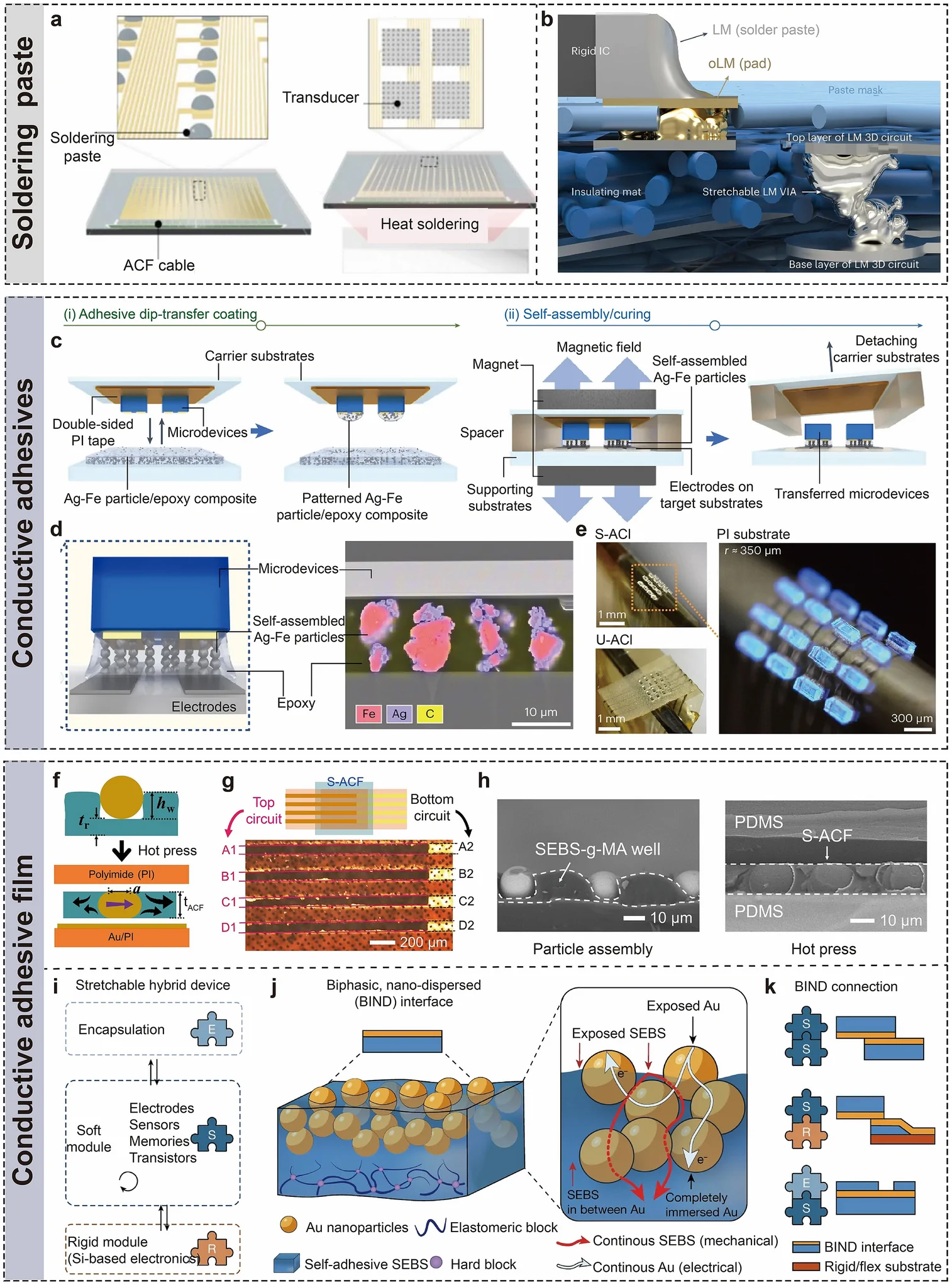
**a** Schematic design of connection by soldering paste. Reproduced from Ref. [270] with permission, copyright 2022, American Association for the Advancement of Science. **b** Schematic design of connection by LM soldering paste. Reproduced from Ref. [24] with permission, copyright 2024, Springer Nature. **c** Schematic of the two main processes of S-ACI. **d** schematic showing microdevices interconnecting to electrodes with Ag–Fe particles. **e** Photographs for integrated microdevices. Reproduced from Ref. [273] with permission, copyright 2024, Springer Nature. **f** Scheme of the polymer flow during the hot pressing. **g** Top-view scheme and the corresponding OM image of the S-ACF (blue). The S-ACF was sandwiched between top and bottom Au circuit lines. **h** Cross-sectional SEM image taken after the particle assembly in the template and the S-ACF after the hot pressing. Reproduced from Ref. [276] with permission, copyright 2021, American Association for the Advancement of Science. **i** Schematic shows stretchable hybrid devices are typically assembled from three elementary modules (encapsulation, soft, and rigid modules), through modular interfaces. **j** Illustration of a biphasic, nano-dispersed (BIND) interface. **k** BIND connection between different modules. Reproduced from Ref. [280] with permission, copyright 2023, Springer Nature

When using traditional soldering paste, the soldering process typically requires heating [270] (Fig. 11a). For thermally intolerant flexible substrates, the soldering process can damage the substrate and even affect device performance [271]. In addition, conventional solder joints are mechanically rigid, making them susceptible to crack formation, delamination, and contact-resistance increases under repeated skin-induced deformation.

Compared with traditional soldering paste, LM soldering paste, owing to their low melting points, high electrical conductivity, and tunable wettability, is emerging as a new type of solder. LM solders assisted by HCl vapor can solder chips onto flexible substrates for microelectronics integration [272]. To reduce the reliance on environmentally hazardous reagents, Zhuang et al. developed an ultra-stretchable hybrid LM solder that can bond rigid electronic components onto fibrous poly(styrene-block-butadiene-block-styrene) (SBS) substrates [24] (Fig. 11b). Although LM-based interconnects improve stretchability, their long-term use requires stable oxide management, leakage prevention, encapsulation integrity, and compatibility with skin-contacting materials.

Bonding electronic components with conductive adhesives is a commonly used strategy in flexible electronics. However, some commercially available Ag pastes or conductive epoxies exhibit mechanical mismatch (excessive rigidity) or poor interfacial adhesion at the bonding interfaces. These issues can be alleviated by minimizing the contact area of conductive adhesives [273] or by adjusting the polymer components in the adhesive formulations [274, 275]. For example, Yoon et al. reported a method for the site-selective anisotropically conductive integration [273]. In the method, an epoxy composite containing Ag–Fe NPs was selectively deposited onto microdevice arrays through magnetic self-assembly (Fig. 11c–e). For prolonged wear, adhesive interconnects must also resist swelling, drying, creep, and filler-network disruption caused by sweat exposure and cyclic strain.

Conductive adhesive films are a class of polymer-based bonding materials that are prefabricated in thin-film form, including anisotropic conductive films (ACFs) and isotropic conductive adhesive films (ICAFs). In ACFs, conductive particles dispersed in the adhesive matrix enable Z-axis conduction with lateral insulation during thermal/pressure bonding. However, ACFs mainly rely on thermal compression bonding to form electrical interconnections, in which temperature is a critical parameter. To minimize the required bonding temperature, Hwang et al. designed a stretchable ACF (S-ACF), in which conductive microparticles were periodically arranged in a thermoplastic block copolymer film to form strong covalent bonds at the contact interfaces at a mild temperature (80 °C) (Fig. 11f–h) [276].

Another type, ICAF, relies on continuous percolation networks formed by conductive fillers within the adhesive film to achieve XYZ isotropic conduction and is usually combined with patterning techniques. Composites consisting of polymer or hydrogel matrices with conductive fillers have been used [277, 278]. However, their large thickness (tens to hundreds of micrometers) leads to mechanical mismatch and reduced mechanical or electrical robustness. Introducing conductive particles or NPs into specific region, typically at the top or bottom of the adhesive films to construct a biphasic, nano-dispersed heterogeneous film can significantly reduce the interconnect thickness while maintaining stable electrical performance (Fig. 11i–k) [279, 280]. Such thin and mechanically graded interfaces are particularly important for reducing strain concentration, suppressing delamination, and maintaining low contact resistance during long-term body motion.

In practical wearable diagnostic systems, different interfacial technologies are selected according to the mechanical and electrical requirements of the integration site. Traditional soldering paste is mainly suitable for locally rigid or thermally tolerant regions, such as flexible printed circuit boards. LM-based soldering paste is more appropriate for stretchable soft–rigid interfaces, including chip integration on elastomeric substrates, fibrous textiles, deformable electrodes, and e-skins, but its practical use requires careful consideration of liquid metal leakage, oxidation, and long-term encapsulation stability. Conductive adhesives are commonly used for low-temperature bonding of temperature-sensitive substrates, such as hydrogel sensors and polymeric flexible sensors. ACFs are particularly useful for high-density directional interconnections, such as bonding flexible sensor arrays, thin-film electrodes, or microchips to flexible readout circuits, because they provide Z-axis conduction while preventing lateral short circuits. In contrast, ICAFs are suitable for large-area or patterned conductive bonding where multidirectional conduction is acceptable, such as attaching flexible electrodes or conductive layers to wearable patches and textile-based systems. ICAFs are promising for long-term skin-mounted electronics and high-resolution sensor arrays, where ultra-thin interconnects, mechanical grading, reduced strain concentration, and stable contact resistance under repeated deformation are required.

#### 3D Integration Strategies

Flexible electronics do not merely consist of single-function devices but rather represent integrated systems that combine multiple functionalities for healthcare and medical applications. A complete wearable diagnostic system typically comprises multiple functional modules, including both flexible and rigid components. If these modules are simply arranged within a single planar layout, the system inevitably encounters challenges related to integration density—such as excessive footprint, which is unfavorable for wearable applications—as well as compromised performance. 3D integration of multiple functional modules enables high-density integration within a limited footprint, helping to reduce parasitic resistance, capacitance, and signal delay, while suppressing noise coupling and cross talk and enhancing overall system design flexibility. To realize multilayer 3D integration of heterogeneous functional devices, two principal strategies have been developed: 3D monolithic integration and 3D hybrid stacking integration.

**Fig. 12:**
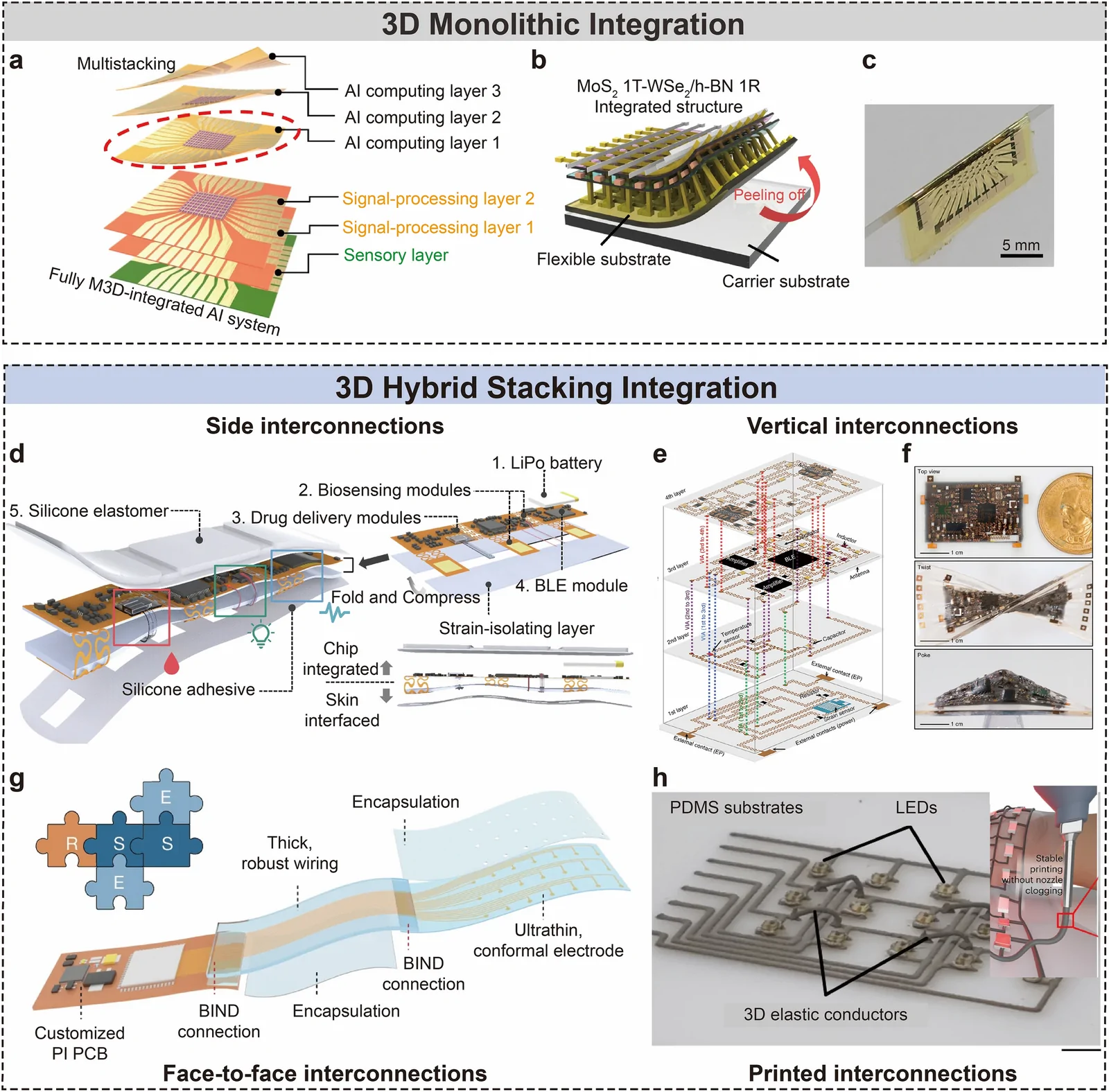
**a** Schematic illustration of ultimate edge computing system based on 3D monolithic integration. **b** Schematic diagram of peelable 3D monolithic-integrated devices. **c** Optical image of 3D monolithic-integrated device array. Reproduced from Ref. [282] with permission, copyright 2023, Springer Nature. **d** Schematic design of side interconnections. Reproduced from Ref. [61] with permission, copyright 2025, Springer Nature. **e–f** Schematic design of vertical interconnections and optical micrographs of the system through vertical interconnections. Reproduced from Ref. [247] with permission, copyright 2018, Springer Nature. **g** Schematic design of face-to-face interconnections. Reproduced from Ref. [280] with permission, copyright 2023, Springer Nature. **h** Schematic design and optical micrograph of printed interconnections. Scale bars, 2 mm. Reproduced from Ref. [285] with permission, copyright 2023, Springer Nature

3D monolithic integration represents a “growth-like” 3D integration strategy, characterized by the bottom-up, sequential construction of functional layers directly on the same flexible substrate or on previously fabricated layers. Rather than relying on physical stacking or adhesive bonding, multilayer devices are formed through repeated cycles of thin-film deposition, patterning, and interlayer insulation, resulting in highly cohesive multilayer architectures. For example, Kwon et al. demonstrated scalable 3D integration of dual-gate organic transistors on plastic foils via printing techniques [281]. Kang et al. reported 3D monolithic integration of fully two-dimensional (2D) material-based electronics, highlighting wafer-free device stacking (Fig. 12a–c) [282]. In their work, six layers of transistor and memristor arrays were vertically integrated into a 3D nanosystem to perform AI tasks by peeling and stacking AI processing layers fabricated from bottom-up synthesized 2D materials.

3D hybrid stacking integration follows a more “modular assembly”-oriented strategy. In this approach, individual functional layers or modules are first fabricated independently under processing conditions optimized for their specific material and performance requirements. These modules are subsequently assembled into a 3D system using diverse interconnection methods, including side interconnections [61], vertical interconnects [24, 283], face-to-face interconnects [61, 280], and printed interconnections [284, 285] (Fig. 12d–h).

From the perspective of practical wearable healthcare applications, monolithic integration and hybrid stacking integration exhibit distinct applicability scenarios due to their different structural characteristics and fabrication strategies. 3D monolithic integration is generally more suitable for wearable systems requiring high integration density, minimized interconnect latency, lightweight form factors, and excellent mechanical continuity, since functional layers are sequentially fabricated on the same substrate to form highly compact architectures with strong interlayer coupling. Such characteristics make monolithic integration particularly advantageous for miniaturized epidermal electronics, and emerging sensing–memory–computing integrated chips for intelligent wearable diagnostic systems. However, achieving high-density vertical interconnections and complex heterogeneous integration remains hindered by material and fabrication constraints. Furthermore, the inherent design complexity of monolithic architectures presents formidable barriers to overall system scalability. For long-term use, monolithic systems also require careful control of residual stress, interlayer adhesion, encapsulation defects, and thermal or chemical compatibility between sequentially processed layers.

In contrast, 3D hybrid stacking integration has emerged as one of the most widely adopted integration approaches in current wearable diagnostic systems due to its superior flexibility in heterogeneous system assembly and modular integration. The diversity of 3D interconnection strategies provides favorable trade-offs among electrical performance, mechanical reliability, integration density, and manufacturability, thereby promoting their widespread adoption in wearable healthcare, biomedical engineering, and soft robotics applications. Benefiting from these advantages, multilayer integration architectures enable the heterogeneous assembly of sensing, energy, processing, and communication modules into compact and multifunctional wearable platforms, thereby bridging the gap between individual device fabrication and system-level implementation. Such integration strategies are particularly suitable for multifunctional wearable diagnostic systems requiring the simultaneous integration of biochemical sensors, electrophysiological monitoring modules, wireless communication units, energy harvesting/storage components, and AI-assisted data processing circuits, especially in applications involving e-skin, continuous physiological monitoring, intelligent therapeutic patches, and closed-loop healthcare systems [23, 286–288]. However, as the degree of vertical integration and structural complexity continues to increase, maintaining stable vertical electrical conduction, precise interlayer alignment, efficient heat dissipation, and robust mechanical integrity under repeated bending, compression, and stretching becomes increasingly challenging. Therefore, beyond achieving high integration density and multifunctionality, considerable attention must also be devoted to the long-term operational stability of stacked architectures.

#### Fully Flexible Electronic Integration

With the continuous advancement of fabrication technologies, fully flexible electronic integration has emerged as an alternative strategy for wearable diagnostic systems. Fully flexible electronic integration refers to systems in which all functional units are constructed entirely from intrinsically flexible or deformable materials, without incorporating any rigid silicon components. This strategy has been developed to eliminate mechanical mismatch at material and device interfaces, thereby improving conformability, mechanical reliability, and long-term stability under bending, stretching, and continuous operation. At present, a complete fully flexible electronic system is generally composed of several essential functional modules, such as sensing units, signal-conditioning units, memory and control units, and energy and communication unit.

**Fig. 13:**
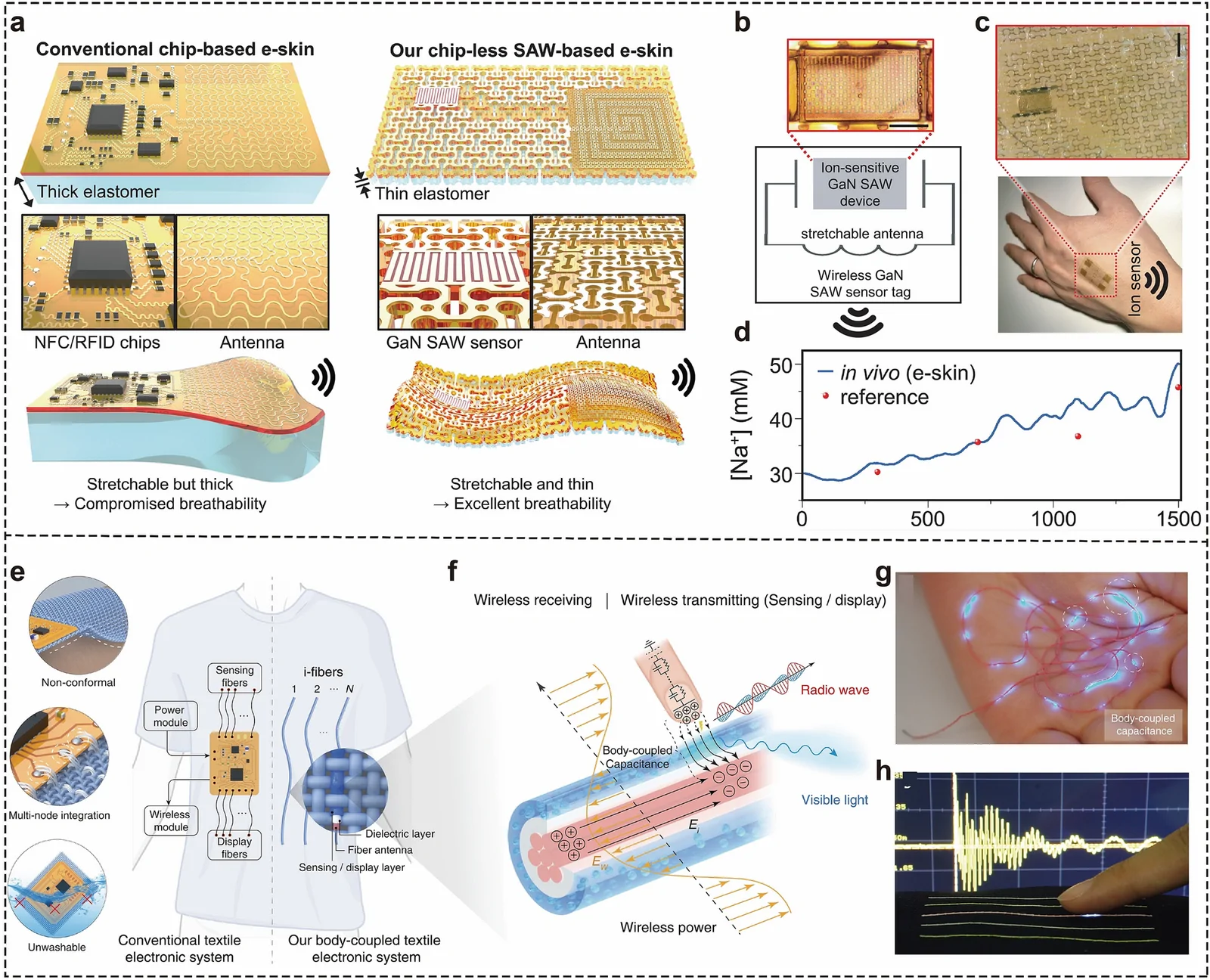
**a** Comparison between (left) conventional wireless e-skin based on integrated circuit chips and (right) the chipless wireless e-skin based on SAW devices made of GaN freestanding membranes. **b** Schematic illustrations and optical microscope image of wireless ion sensors based on GaN SAW device. Scale bar, 200 µm. **c-d** Photograph and microscopy image of an e-skin attached on the back of a hand, in vivo recordings of the variation in Na^+^ ion concentration in sweat using wireless GaN SAW-based e-skin (blue) and a reference conductometer (red). Reproduced from Ref. [289] with permission, copyright 2022, American Association for the Advancement of Science. Scale bar, 500 µm. **e** Comparison between (left) current wireless interactive textile system based on integrated circuit chips and (right) our chipless, wireless interactive textile system. **f** Principle of body-coupled chipless i-fiber. **g** Photograph of i-fiber being wirelessly powered by placing it on the hand and coupling the surrounding EM energy. **h** Embroidered i-fiber can simultaneously generate both optical and electrical signals through touching. Reproduced from Ref. [268] with permission, copyright 2024, American Association for the Advancement of Science

Among these, wireless data communication represents a key technical requirement. To address this requirement, Kim et al. reported a chipless wireless e-skin based on surface acoustic wave (SAW) sensors fabricated from freestanding ultra-thin single-crystalline piezoelectric gallium nitride (GaN) membranes. This SAW-based e-skin enables highly sensitive, low-power, and long-term sensing of strain, ultraviolet light, and ion concentrations in sweat (Fig. 13a–d) [289].

In parallel, another fully flexible integration strategy has focused on intelligent electronic textiles, in which individual fibers themselves perform the functions of wireless transmission, sensory processing, and feedback, thereby serving as fundamental building blocks for electronic textile systems [290]. For instance, Yang et al. proposed a chipless body-coupled energy interaction mechanism that enables ambient electromagnetic energy harvesting and wireless signal transmission through a single fiber. In this approach, the fiber itself supports wireless visual–digital interactions without the need for additional chips or batteries integrated into the textile (Fig. 13e–h) [268]. By leveraging wireless chipless communication, fiber-level multifunctional integration, recent advances are overcoming the limitations imposed by conventional silicon-based electronics.

Despite these advances, the clinical translation of battery-free or chipless fully flexible systems still requires reliable wireless performance under practical operating conditions. Key parameters, including resonance stability, readout distance, signal strength, and wireless coupling, should remain stable during body motion, sweat exposure, and variable device placement. Moreover, textile-based and fiber-integrated platforms should be systematically evaluated under washing, abrasion, repeated stretching, skin friction, and long-term wear to ensure that their mechanical compliance is matched by durable electrical, sensing, and communication performance.

## Biomedical Applications

The goal of advancing wearable diagnostic electronics is to support a clinically meaningful transition from episodic, hospital-based checkups to continuous, personalized health management. While traditional diagnostic modalities often provide only “snapshot” data that may overlook transient pathological signals or interindividual variability, wearable platforms can complement clinical workflows by capturing longitudinal physiological and biochemical information in daily-life settings. Thus, their clinical rationale lies not simply in miniaturizing laboratory or hospital instruments, but in addressing unmet needs such as continuous monitoring, early detection of transient events, individualized baseline construction, decentralized follow-up, and low-burden assessment of treatment response. By leveraging the advanced fabrication strategies and robust system integrations discussed in previous sections, these devices can establish individualized physiological baselines or “digital twins,” enabling the detection of subtle anomalies that may support early warning, risk stratification, treatment-response assessment, and long-term follow-up. However, the clinical value of wearable diagnostics depends not only on sensing performance, but also on their validation against established clinical gold standards, reliability under real-world use, reproducibility across users and devices, and compatibility with regulatory and clinical decision-making frameworks. In this section, we summarize major biomedical applications of wearable diagnostics, highlighting their sensing targets, device architectures, clinical motivations, benchmarking against conventional methods, and remaining translation gaps. To provide a chapter-level overview, Table 3 summarizes representative clinical validation requirements for wearable precision diagnostic systems, including wearable measurements, current clinical reference methods, key validation endpoints, and major translational gaps across different disease scenarios.

**Table 3:** Clinical validation requirements for wearable precision diagnostic systems

Biomedical application	Wearable measurement	Gold standard	Key validation endpoints	Major translational gaps	References
Nervous system	Wearable EEG, EOG, sleep or drowsiness signals	Clinical EEG, ambulatory EEG, polysomnography, neurological examination	Waveform agreement, sleep-stage accuracy, event-detection sensitivity, artifact robustness area, high-density arrays	Hair interference, motion artifacts, electrode–skin instability, AI external validation interference	[286, 291–299]
Cardiovascular system	ECG, PCG, PPG, multimodal cardiac signals	12-lead ECG, Holter monitoring, digital stethoscopy, echocardiography	Lead-specific waveform fidelity, arrhythmia sensitivity, false-alarm rate, long-term signal stability	Motion artifacts, lead-placement variability, soft-hard interface fatigue	[294, 300–305]
Blood pressure	Pulse wave, PPG, PAT, wearable ultrasound	Office cuff BP, ambulatory BP monitoring, invasive arterial pressure, Doppler ultrasound	Absolute BP error, trend accuracy, Bland–Altman agreement, hypertension classification	Calibration drift, vascular stiffness, posture and activity dependence	[306–312]
Respiratory System	Respiratory rate, lung sounds, cough, EBC biomarkers, VOCs, viral antigens	Spirometry, capnography, clinical auscultation, RT-qPCR, antigen testing, sputum culture	FEV_1_ prediction, respiratory-rate error, diagnostic sensitivity and specificity, false-positive and false-negative rates	Mask leakage, humidity variation, environmental contamination, matrix interference	[313–321]
Ocular system	Contact-lens IOP, tear microenvironment, eyelid-motion signals	Goldmann or rebound tonometry, slit-lamp examination, OCT, visual-field testing, ophthalmologist assessment	IOP agreement, ocular-motility classification, corneal safety, comfort during prolonged wear	Tear-film variability, blinking artifacts, oxygen permeability, inter-user anatomy	[244, 322–326]
Skeletal muscle system	Strain, pressure, EMG, wearable ultrasound, biochemical muscle markers	Dynamometry, clinical EMG, ultrasound, MRI, CT, DXA, functional mobility scores	Imaging agreement, muscle-force correlation, disease-severity classification, rehabilitation outcome tracking	Probe-skin coupling, deep-tissue resolution, activity confounders, limited patient data	[327–333]
Skin	Pressure, temperature, hydration, TEWL, pH, glucose, lactate, wound gases	Dermatological examination, SCORAD, EASI, PASI, corneometry, TEWL instruments, wound scores, microbial culture	Barrier-function agreement, infection detection, wound-healing trajectory, treatment-response prediction	Exudate interference, biofilm formation, dressing variability, irritation and sensitization	[334–344]
Oncology	Tumor volume, bioimpedance, ctDNA, VOCs, therapeutic drug levels	Histopathology, CT, MRI, PET, RECIST criteria, plasma ctDNA assays, GC–MS, therapeutic drug monitoring	Early-detection accuracy, tumor-response agreement, biomarker correlation, longitudinal tracking	Low biomarker abundance, tumor heterogeneity, limited specificity, confounding inflammation	[345–351]
Diabetes	ISF, sweat or tear glucose, drug levels, multimodal cardiometabolic signals	Finger-prick or venous glucose, HbA1c, oral glucose tolerance testing, commercial CGM	MARD, error-grid analysis, lag time, hypoglycemia and hyperglycemia detection, skin tolerability	Biofluid-blood mismatch, enzyme degradation, calibration drift, closed-loop safety	[352–358]
Inflammation and immune response	CRP, IL-6, IgG, cytokines in sweat or ISF	ELISA, chemiluminescent immunoassays, blood CRP or cytokine panels, clinician-adjudicated outcomes	Sensitivity, specificity, paired blood-sample agreement, flare or infection detection, cohort validation	Low abundance, slow diffusion, biofouling, cross-reactivity	[359–364]
Other biomedical applications	Cortisol, estradiol, fetal motion, oral pH, ISF Na^+^	Psychometric scales, cortisol assays, hormone assays, ultrasound, cardiotocography, dental examination, serum electrolytes	Physiological-clinical correlation, event-detection accuracy, longitudinal trend agreement, outcome relevance	Low disease specificity, behavioral confounders, biofluid lag, unclear clinical thresholds	[365–368]

### Nervous System

**Fig. 14:**
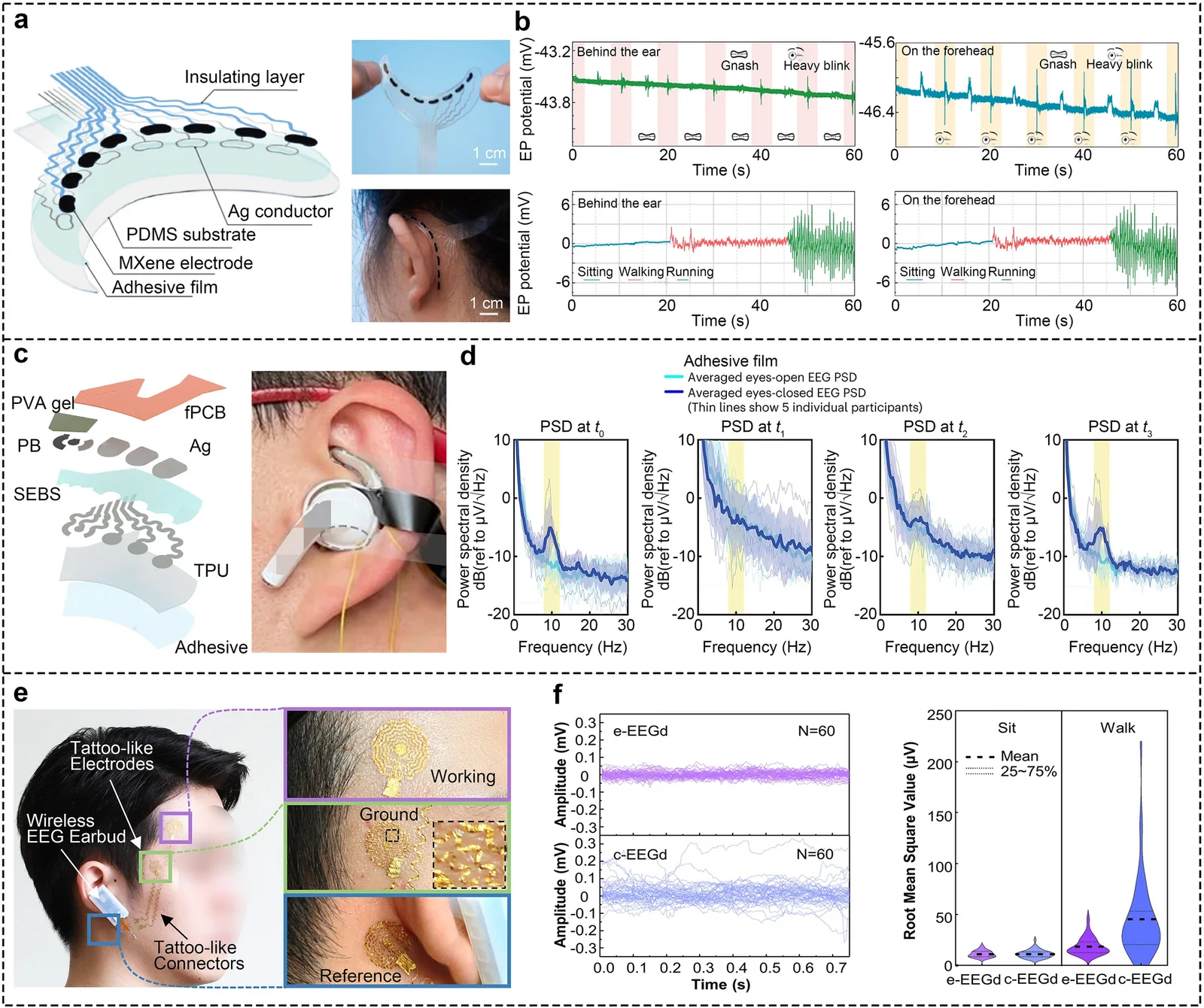
Wearable diagnostic electronics for neurological disorders diagnosis and monitoring. **a** Breathable multichannel ear-computer interface patch for long-term electrophysiological recording. **b** EEG waveforms recorded during jaw-gnashing, blinking, sitting, walking, and running. Reproduced from Ref. [296] with permission, copyright 2026, Elsevier BV. **c** In-ear sensor array for simultaneous EEG and sweat lactate monitoring. **d** PSD analysis of alpha modulation before and after exercise. Reproduced from Ref. [297] with permission, copyright 2023, Springer Nature. **e** Wearable EEG system integrated into a Brain-AI closed-loop framework. **f** EEG recordings and RMS analysis showing reduced motion artifacts during walking. Reproduced from Ref. [298] with permission, copyright 2022, Springer Nature

The brain, as the most complex organ in the human body, governs virtually every physiological and cognitive function. Neurological disorders, such as epilepsy and neurodegenerative diseases, coupled with the rapid rise of brain-machine interfaces, necessitate high-fidelity, long-term monitoring of EEG signals [369]. In current clinical practice, neurological assessment still relies primarily on hospital-based EEG, polysomnography, neuroimaging, and physician-guided neurological examination [293]. Although these methods provide high diagnostic confidence, they are often constrained by short recording windows, bulky wired systems, limited access outside specialized centers, and reduced ability to capture infrequent or context-dependent events in daily life. Therefore, wearable neural devices are best positioned as complementary tools for continuous screening, event detection, risk stratification, and home-based follow-up rather than immediate replacements for established diagnostic systems. A primary challenge in clinical and consumer settings is maintaining stable electrode–skin contact amidst hair interference and motion artifacts. To address this, a “stick-and-play” hairlike device was developed using flexible, stretchable materials and robust bioadhesives to ensure stable scalp contact without conductive gels [295]. Similarly, an 8-channel ear–computer interface patch was fabricated via inkjet printing MXene electrodes onto breathable medical film for discreet, behind-the-ear monitoring (Fig. 14a) [296]. Time-domain waveforms demonstrate that EEG potentials captured behind the ear are significantly less affected by facial muscle deformations compared to forehead sites (Fig. 14b). These interface innovations bridge the gap between bulky clinical caps and imperceptible consumer electronics, enabling reliable mental status monitoring during strenuous daily activities.

The evolution of neural wearables has further moved toward unobtrusive, multimodal platforms. An in-ear integrated array was developed for the simultaneous monitoring of EEG, electrooculography (EOG), and sweat lactate concentration via a flexible substrate (Fig. 14c) [297]. The averaged power spectral density (PSD) transitions across alpha and beta bands before and after exercise are captured (Fig. 14d), revealing the synergetic interactions between metabolic and electrophysiological biomarkers. Furthermore, wireless, dry-electrode earpieces were showcased for continuous drowsiness monitoring in pilots and drivers [370]. By utilizing gold-plated electrodes and offline support vector machine (SVM) classifiers, this system achieved an automated drowsiness detection accuracy of 93.3% for previously unseen users. These earpiece-based systems facilitate longitudinal brain monitoring in real-world environments while preserving user comfort and ecological validity in daily-life settings. Importantly, the clinical translation of such multimodal platforms is gaining momentum across multiple neurological domains. In epilepsy, wearables combining EEG, EMG, and accelerometry have improved the sensitivity of tonic–clonic seizure detection compared with single-modality approaches, while prospective real-world studies with hundreds of days of ambulatory recording have revealed patient-specific seizure periodicities that are difficult to capture by short-term clinical monitoring [291, 292]. Beyond epilepsy, multimodal systems integrating electrophysiological, kinematic, and autonomic signals have been used in Parkinson’s disease to quantify motor fluctuations, dyskinesia, and sleep disturbances as longitudinal digital biomarkers of treatment response in home settings [286]. Together, these advances suggest that unobtrusive multimodal neural platforms are moving from proof-of-concept studies toward evidence-based clinical tools.

Recent advances have further converged self-powering technologies and embedded AI to realize closed-loop neurological systems capable of autonomous intervention and on-device diagnostics. A Brain-AI Closed-Loop System (BACLoS) was introduced using an earbud-like wireless EEG device with tattoo-like electrodes to enhance machine decision-making through error-related potential analysis (Fig. 14e) [298]. The e-EEGd’s artifact-resistance is highlighted in Fig. 14f, where root-mean-square (RMS) values during walking show the device generates 8.5 times less noise than commercial alternatives. Additionally, a self-powered EEG monitoring system utilizing a double-network antifreeze hydrogel sensor was developed for sleep health management [299]. This system employs variational mode decomposition and long short-term memory networks to achieve efficient real-time sleep staging without external power. Collectively, these AI-driven, self-sustaining platforms represent the pinnacle of neurotechnology, paving the way for “human-in-the-loop” ML and precision home-based neurological care. At present, wearable AI frameworks have begun to provide clinical decision support for nervous system diseases in hospital and outpatient settings. However, the clinical translation of autonomous, deeply integrated EEG diagnostic platforms will require standardized prospective validation against expert-adjudicated reference datasets, algorithmic benchmarking under regulator-recognized good ML practice frameworks, and coordinated efforts to build interoperable, privacy-preserving data infrastructures capable of supporting the longitudinal evidence base required for precision neurological care.

### Cardiovascular System

The cardiovascular system is fundamental to sustaining life, as the precise coordination between cardiac electrical signaling and hemodynamic regulation ensures continuous blood circulation, and any disruption to this system can precipitate life-threatening conditions such as arrhythmias and heart failure. In the comprehensive functional assessment of the cardiovascular system, the synchronized monitoring of electrocardiogram (ECG) and blood pressure (BP) represents one cornerstone of precision cardiovascular diagnosis. While ECG captures the electrophysiological “commands” initiated by the heart, BP reflects the net mechanical output resulting from the coordinated interplay between cardiac pumping function and vascular network dynamics—together providing a holistic picture of cardiovascular status that neither modality can achieve in isolation [371–374]. Conventional clinical evaluations rely on episodic ECG recordings, Holter monitors, or intermittent cuff-based blood pressure measurements, all of which share a fundamental “snapshot” limitation: They fail to capture transient pathological events, dynamic fluctuations such as nocturnal surges, or individual physiological baselines essential for precision diagnosis—limitations further compounded by artifacts such as the “white-coat effect.” This creates an urgent clinical demand for continuous, long-term monitoring in natural living environments. Wearable diagnostic electronics have emerged to address this need, enabling personalized 24-h physiological profiling for early detection of rhythm anomalies and BP disorders, while complementing established clinical standards such as 12-lead ECG, Holter monitoring, ambulatory BP monitoring, cuff-based sphygmomanometry, and invasive arterial BP measurement in high-acuity settings. These capabilities are supporting a gradual shift in cardiovascular care from reactive intervention to proactive, precision health management.

**Fig. 15:**
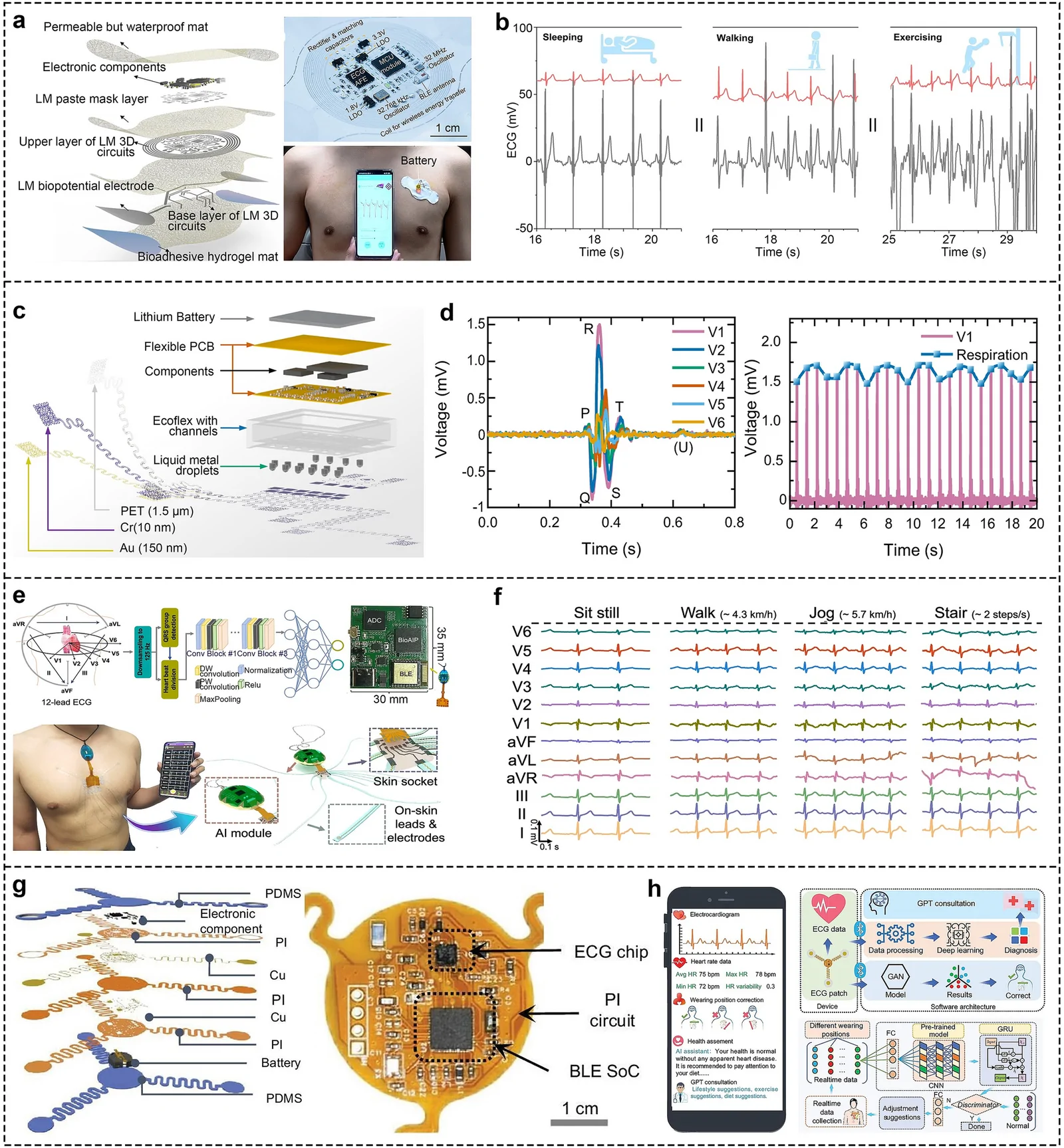
Wearable diagnostic electronics for ECG monitoring. **a** ICU-grade breathable cardiac e-skin. **b** Continuous ECG acquisition and activity-dependent performance of BreaCARES. Reproduced from Ref. [300] with permission, copyright 2025, American Association for the Advancement of Science. **c** Chest-scale self-compensated epidermal electronics for 6-precordial-lead ECG recording. **d** V1-V6 ECG signals and synchronized respiratory waveforms. Reproduced from Ref. [301] with permission, copyright 2022, Springer Nature. **e** MU-DCG system based on imperceptible electronics. **f** Off-skin module and 12-lead ECG recordings during daily activities. Reproduced from Ref. [302] with permission, copyright 2025, Springer Nature. **g** AI-enhanced flexible ECG patch. **h** Software interface for ECG visualization, arrhythmia diagnosis, and medical consultation. Reproduced from Ref. [304] with permission, copyright 2025, Oxford University Press

In the development of wearable continuous ECG monitoring systems, maintaining excellent electrode–skin interface impedance is a critical prerequisite for ensuring signal acquisition accuracy. For instance, Li et al. [294] developed a flexible MN array dry electrode that penetrates the stratum corneum without reaching the dermis, achieving a record-low normalized contact impedance of 0.98 kΩ cm^2^ at 1 kHz. Beyond reducing impedance, ensuring long-term biocompatibility and comfort remains crucial. A notable advancement in this area is the BreaCARES system, an intensive care unit (ICU)-grade breathable cardiac e-skin utilizing multilayer-patterned LM microcircuits (Fig. 15a) [300]. This ultra-lightweight platform (0.489 g) maintains a stable biointerface even during vigorous activity, preventing skin erythema over week-long attachments while providing superior signal-to-noise ratios (SNRs) and stability across various daily scenarios (Fig. 15b). However, single-patch ECG monitoring systems remain insufficient to meet the fidelity requirements of clinical-grade diagnostics, driving the development of large-scale sensing arrays and multimodal integration strategies that bridge the gap between portable wearable patches and standard clinical configurations. Yin et al. [301] introduced a chest-scale epidermal system for 6-precordial-lead ECG, employing a self-compensation strategy with epidermal branches to eliminate interference from long interconnects (Fig. 15c, d). This concept of an “invisible doctor” is further embodied in the motion-unrestricted dynamic ECG (MU-DCG) system (Fig. 15e), which utilizes ultra-thin electrodes (< 50 μm) and a pressure-activated flexible socket to achieve motion-artifact-free 12-lead acquisition during daily activities [302]. As shown in Fig. 15f, the MU-DCG maintains clear waveforms and high fidelity across diverse exercise intensities, ranging from sitting to ascending stairs.

Beyond electrical sensing, the synergy of ECG with phonocardiogram (PCG) signals provides a more holistic mechanical–electrical profile of the heart. For example, a wearable system paired a contact-type lead zirconate titanate (PZT) heart sound sensor with ECG electrodes, achieving high SNRs of 44.13 and 30.04 dB, respectively [303]. To complete the “diagnosis-to-therapy” loop, Lee et al. [61] demonstrated a wireless heterogeneous skin patch that integrates ECG/photoplethysmography (PPG) monitoring with a drug-delivery micropump. The folded structure and strain-isolating elastomer ensure intimate skin contact, enabling stable acquisition of multimodal signals and tracking of 3D motion even during complex sitting-to-standing transitions. Clinically, the value of these multimodal platforms will depend on whether combined electrical, acoustic, optical, and hemodynamic readouts improve risk stratification or treatment-response assessment beyond single-parameter monitoring.

AI serves as the final, critical layer for transforming these hardware signals into actionable diagnostic insights. An AI-enhanced ECG patch capable of withstanding 100% strain was developed by Huang et al., which leverages an interpretable attention transformer model to identify heart diseases with up to 98% accuracy (Fig. 15g) [304]. This intelligent system even addresses practical deployment issues, using generative adversarial network-based algorithms to ensure 85% accuracy in device positioning and integrating GPT-based consultation interfaces for real-time medical advice within one second (Fig. 15h). Nevertheless, AI-enabled cardiovascular wearables require prospective validation in independent patient cohorts, transparent reporting of training and testing datasets, and regulatory evaluation of algorithm robustness, cybersecurity, and clinical decision support boundaries before they can be used as reliable diagnostic tools.

Hypertension, often termed the “silent killer,” is a leading global risk factor for stroke, heart failure, and chronic kidney disease. Despite its prevalence, traditional diagnostic protocols rely heavily on episodic, cuff-based sphygmomanometry, which can miss nocturnal hypertension, non-dipping patterns and transient BP surges that strongly predict cardiovascular events. Even ambulatory cuff-based BP monitoring, although clinically valuable, can disturb sleep through repeated cuff inflation and still provides only intermittent readings rather than beat-to-beat hemodynamic trends. Wearable diagnostic electronics bridge this gap by offering continuous, cuffless BP monitoring. By providing a longitudinal “digital baseline,” these platforms can detect nighttime surges and morning spikes in real time, track BP responses to posture, exercise, stress, medication, and sleep, and support individualized risk stratification beyond isolated clinic measurements. Their key technological contribution is therefore not simply removing the cuff, but enabling unobtrusive, repeated, or continuous hemodynamic assessment in daily life through pulse-wave, PPG/pulse arrival time (PAT), pressure, and ultrasound-based sensing strategies. However, their clinical utility depends on rigorous comparison with cuff-based office BP, ambulatory BP monitoring, and, where appropriate, invasive arterial pressure measurements, particularly in terms of absolute error, trend accuracy, calibration stability, and hypertension classification performance.

**Fig. 16:**
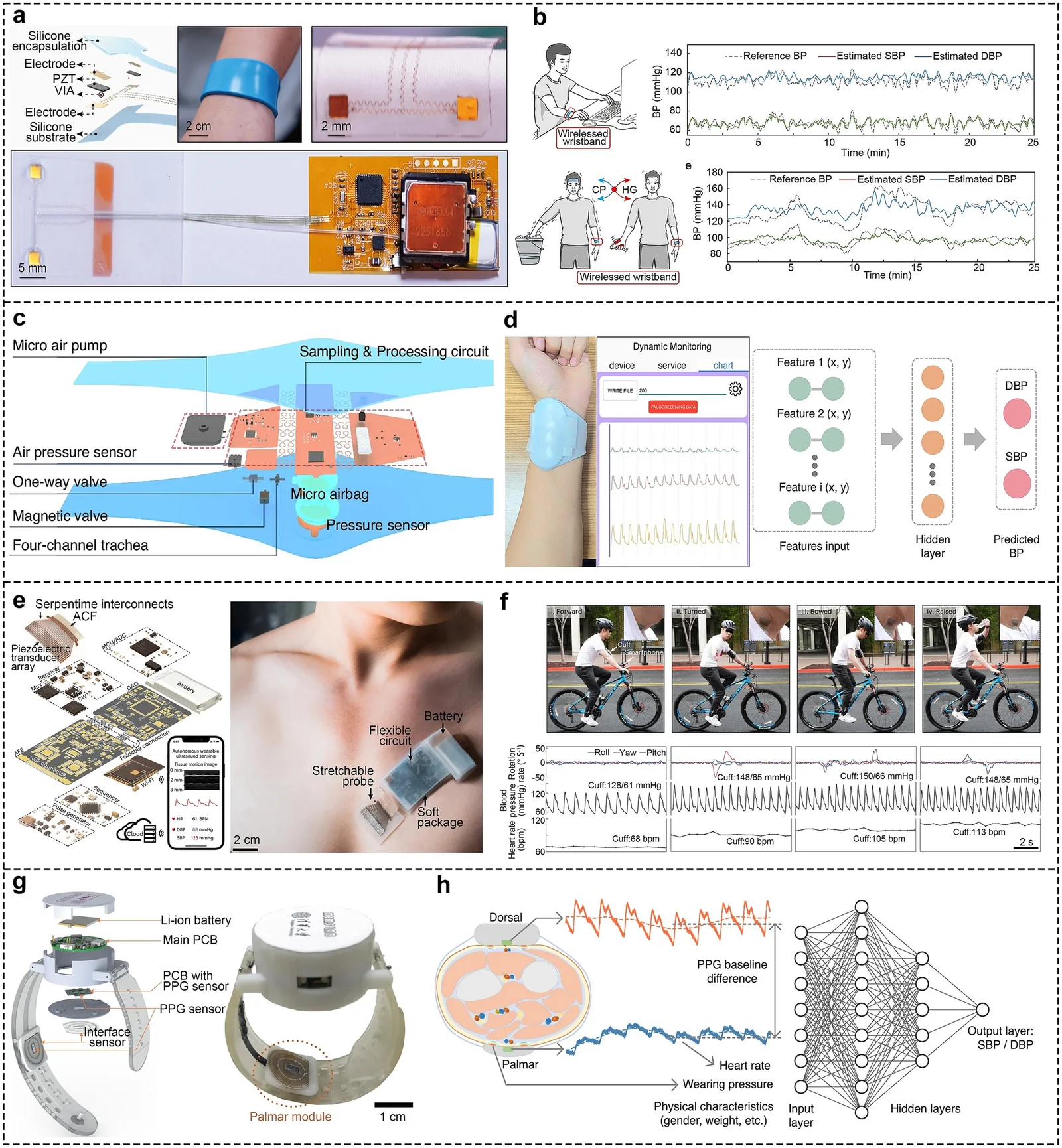
Wearable diagnostic electronics for BP monitoring. **a** Thin, soft, wireless system for continuous arterial BP monitoring. **b** BP tracking during rest, hand-grip, and cold-pressor tests validated against reference devices. Reproduced from Ref. [306] with permission, copyright 2023, Springer Nature. **c** Multichannel active pressurized sensing platform for pulse detection. **d** Mobile interface and model workflow for real-time BP prediction. Reproduced from Ref. [307] with permission, copyright 2024, Springer Nature. **e** Wearable ultrasound-system-on-patch (USoP) for deep-tissue hemodynamic monitoring. **f** USoP-based BP monitoring during cycling and head movement. Reproduced from Ref. [308] with permission, copyright 2024, Springer Nature. **g** Wrist-based multichannel sensing strategy for continuous BP monitoring. **h** Feature extraction and multilayer-perceptron-based data fusion framework. Reproduced from Ref. [310] with permission, copyright 2023, Springer Nature

Li et al. proposed a thin, soft, miniaturized system (TSMS) [306], which combines a self-powered piezoelectric sensor array with an active pressure-adaptive micro-airbag, enabling truly wearable, continuous, and wireless real-time monitoring of BP and pulse-wave velocity (PWV) (Fig. 16a). Figure 16b demonstrates that the TSMS maintains high consistency with clinical gold-standard devices during continuous 25-min monitoring and hand-grip exercise tests. Adopting a conceptual framework analogous to conventional cuff-based sphygmomanometers, Zhao et al. incorporated a flexible airbag array and an active pressure control unit into the wearable BP platform (Fig. 16c) [307]. This innovative three-channel pulse sensing platform simulates finger pressure (Cun, Guan, and Chi) analogous to traditional Chinese medicine pulse diagnosis. BP status is subsequently inferred by analyzing pulse amplitude variations across different external pressures (Fig. 16d). Furthermore, the advent of fully integrated flexible ultrasound patches has enabled a leap in sensing depth. The wearable ultrasonic-system-on-patch (USoP) can track physiological signals from tissues as deep as 164 mm (Fig. 16e) [308]. The USoP continuously monitors carotid pulsation waveforms even during high-intensity exercise or head movements, accurately capturing central BP fluctuations caused by increased cardiac output (Fig. 16f). These studies demonstrate important progress toward wearable BP assessment, but broader patient-cohort validation remains necessary to confirm robustness across age, vascular stiffness, skin type, body position, medication status, and daily activity conditions.

However, sensing systems dedicated solely to ECG or BP monitoring are inherently limited in their ability to fully characterize the electromechanical coupling dynamics of the cardiovascular system. A miniaturized multifunctional epidermal patch (MEP) that integrates ECG electrodes and a piezoresistive nanofiber pulse sensor on a single flexible substrate was proposed [309]. By extracting PAT and utilizing a random forest regression model, the system achieved precise estimation of both systolic and diastolic BP. In addition, the introduction of AI and big data analytics further enhances both diagnostic precision and user experience. Wang et al. [310] reported a wrist-worn method based on dual PPG signals that utilizes differential operations between the palmar and dorsal sides of the wrist to eliminate individual variability (Fig. 16g). As illustrated in Fig. 16h, the system employs a Keras-based neural network framework to fuse PPG signals, contact pressure, and skin temperature, achieving highly generalizable real-time monitoring without the need for tedious subject-specific calibration. Concurrently, an intelligent system based on a highly sensitive strain sensor array and DL was developed to evaluate cardiovascular function without requiring precise device positioning [311]. These advancements signify a leap toward self-adaptive, zero-calibration, and cloud-interactive “digital twins” in BP monitoring. For clinical translation, however, calibration-free or low-calibration algorithms must be validated prospectively against standardized BP references, with transparent reporting of failure cases, population bias, and long-term drift.

### Respiratory System

Serving as the primary interface between the human body and the external environment, the respiratory system is continuously exposed and highly vulnerable to diverse pathological threats. Chronic respiratory diseases, including chronic obstructive pulmonary disease (COPD) and asthma, alongside acute respiratory tract infections (RTIs) such as COVID-19 and influenza, collectively represent a major global health burden characterized by high morbidity and the constant risk of life-threatening exacerbations [375–379]. Conventional diagnostic tools, including stationary spirometers, sputum cultures, and reverse transcription quantitative polymerase chain reaction (RT-qPCR), share critical limitations, including clinic- or laboratory-dependent workflows, limited capacity for continuous symptom tracking, and delayed capture of acute deterioration. Wearable diagnostic electronics have emerged to enable longitudinal surveillance of both physical symptoms (e.g., cough, wheeze) and molecular biomarkers (e.g., viral antigens, inflammatory cytokines), thereby establishing personalized respiratory management frameworks that complement spirometry, clinical auscultation, sputum culture, and nucleic-acid testing, and may shift the clinical paradigm from reactive crisis management to proactive, precision pulmonary care.

**Fig. 17:**
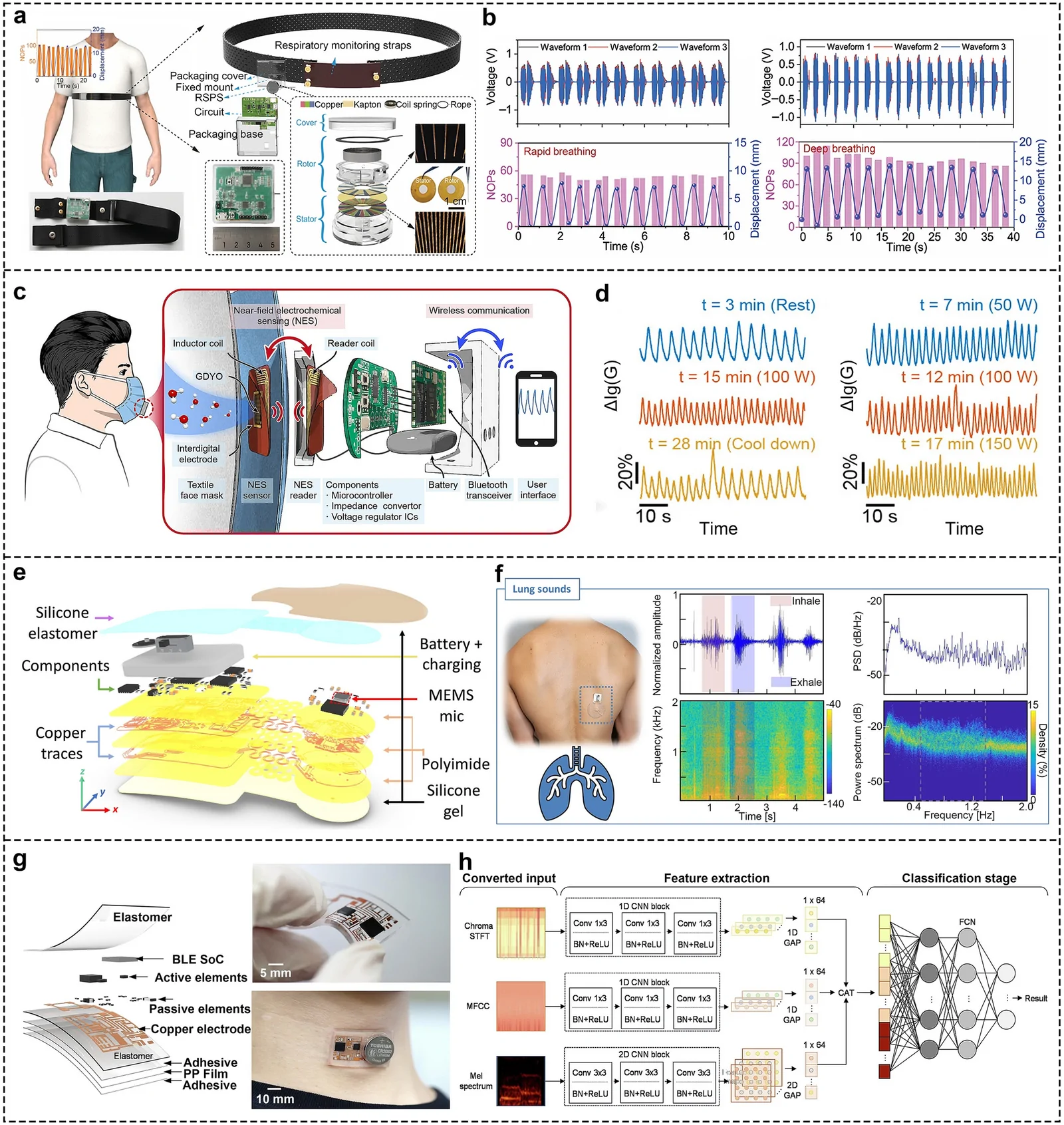
Wearable diagnostic electronics for respiratory. **a** Miniaturized retractable thin-film sensor for continuous respiratory monitoring. **b** Sensor output during rapid and deep breathing patterns. Reproduced from Ref. [313] with permission, copyright 2023, Springer Nature. **c** Smart facemask with near-field electrochemical sensors for respiratory assessment. **d** Real-time respiratory waveforms during stationary cycling. Reproduced from Ref. [314] with permission, copyright 2025, American Chemical Society. **e** Portable SWS for continuous auscultation. **f** Lung-sound signals, spectrograms, and PSD analysis recorded by the SWS. Reproduced from Ref. [315] with permission, copyright 2022, American Association for the Advancement of Science. **g** DL-assisted mechano-acoustic system for respiratory diagnosis. **h** DL workflow for spectral feature extraction and classification. Reproduced from Ref. [317] with permission, copyright 2024, Springer Nature

For sensing to track lung mechanics with high fidelity, an easy-to-fabricate retractable self-powered sensor based on triboelectric nanogenerators was developed to monitor respiratory rate and ventilation with a sensing resolution of 0.13 mm and durability exceeding 1 million cycles (Fig. 17 a, b) [313]. Validation against a commercial spirometer and chest strap confirmed consistent waveform correspondence across breathing patterns including normal respiration, deep breathing, and apnea events. Similarly, near-field electrochemical sensing has been integrated into smart masks, allowing for the wireless monitoring of respiratory humidity and waveforms during varied physical exercises (Fig. 17c, d) [314]. Beyond breath monitoring, capturing internal lung sounds provides deeper diagnostic insights. Lee et al. [315] reported a soft wearable stethoscope (SWS) that detects continuous cardiopulmonary sounds with a high SNR, enabling the clear recording of pathological signals across a frequency range of 100 to 2500 Hz (Fig. 17e, f). Compared with commercial digital stethoscopes, the SWS demonstrated superior SNR after wavelet denoising, and a clinical study involving patients and healthy controls achieved 95% accuracy in classifying four lung disease types. Nevertheless, both platforms rely on healthy volunteer cohorts or limited patient samples as reference standards, and neither has been evaluated against pulmonary function testing or radiological imaging, underscoring the gap between device-level demonstration and clinical deployment.

The integration of respiratory pattern analysis with AI further enables comprehensive pulmonary assessment and predictive diagnostics. A biodegradable paper-based e-nose integrated into masks achieved 89% accuracy for tuberculosis detection by reducing humidity interference with hydrophobic polymer-functionalized single-walled carbon nanotubes [316]. The fusion of high-fidelity hardware with advanced algorithms empowers autonomous diagnosis. Hee et al. [317] demonstrated a DL-assisted mechano-acoustic system that utilizes a convolutional neural network (CNN)-based feature extraction procedure to predict forced expiratory volume in 1 s (FEV_1_) levels with high accuracy (Fig. 17g, h). In a similar vein, an on-mask magnetoelastic sensor network was coupled with a SVM algorithm to achieve a respiration pattern recognition accuracy of 94.03% [318]. These AI-driven platforms represent a promising route toward personalized respiratory management, bridging the gap between raw physiological data and actionable clinical insights for managing chronic pulmonary conditions.

**Fig. 18:**
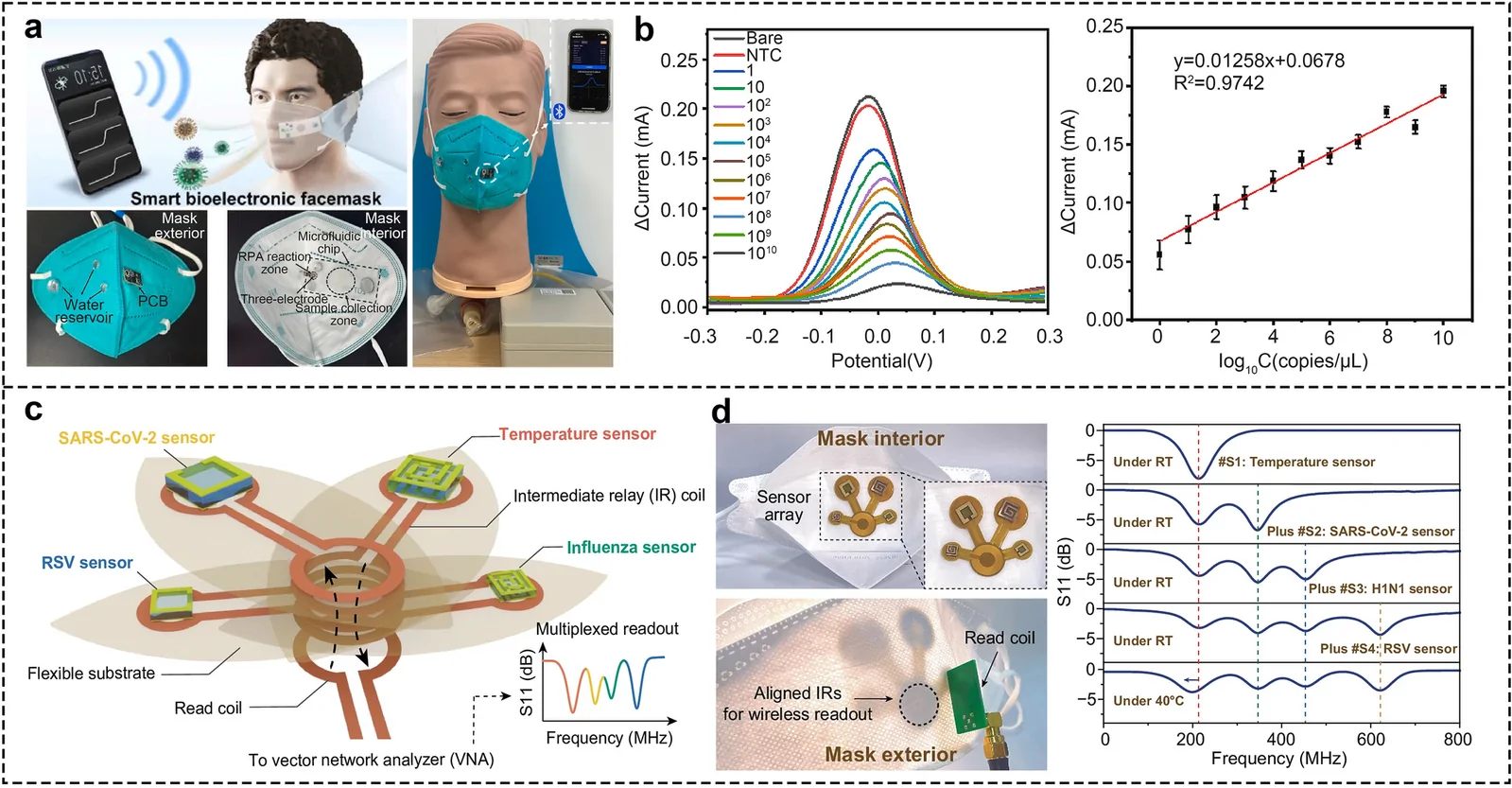
Wearable diagnostic electronics for respiratory-system biomarker detection. **a** Smart bioelectronic facemask for noninvasive detection of respiratory infections. **b** DPV responses and calibration curve for target gene detection. Reproduced from Ref. [320] with permission, copyright 2026, Elsevier BV. **c** Hydrogel-modulated resonator system for wireless immunoassay of respiratory virus aerosols. **d** ImmHR-integrated mask and infrared-mediated multiplexed readout with radio frequency spectrum analysis. Reproduced from Ref. [321] with permission, copyright 2026, Springer Nature

Beyond respiratory pattern monitoring, disease biomarkers present in exhaled breath offer a real-time molecular reflection of the body’s metabolic state and pathological progression, opening a critical “molecular window” for early screening and precision monitoring. The integration of molecular diagnostics and AI represents the current frontier of respiratory surveillance. Heng et al. introduced EBCare, a microfluidic smart mask that performs in situ analysis of exhaled breath condensate (EBC) biomarkers, such as NH_4_^+^ and pH, to assess post-COVID-19 inflammation [319]. For target-specific pathogen detection, a bioelectronic facemask was developed incorporating a recombinase polymerase amplification-coupled biosensor, achieving SARS-CoV-2 detection limits as low as 0.19 copy µL^−1^ within 30 min (Fig. 18a, b) [320]. Additionally, a wireless immunoassay utilizing hydrogel-modulated resonators (ImmHR) enables rapid detection (< 10 min) of H1N1 and respiratory syncytial virus (RSV) antigens at fg L^−1^ levels (Fig. 18c, d) [321], with clinical sample testing in 33 quantitative reverse transcription polymerase chain reaction (qRT-PCR)-confirmed H1N1-positive patients demonstrating clear discrimination from healthy controls. Ultimately, these signals are refined by ML models, such as CNNs and SVMs, to achieve 95% classification accuracy. Despite these encouraging results, genuine clinical translation of such breath-based molecular platforms still requires several critical steps: EBC biomarker reference ranges remain unstandardized across collection protocols and patient populations, the pilot-scale cohorts reported to date are insufficient for establishing diagnostic sensitivity and specificity thresholds comparable to spirometry or bronchoscopy, and neither platform has yet undergone prospective multicenter trials or regulatory evaluation as a clinical diagnostic device.

### Ocular System

**Fig. 19:**
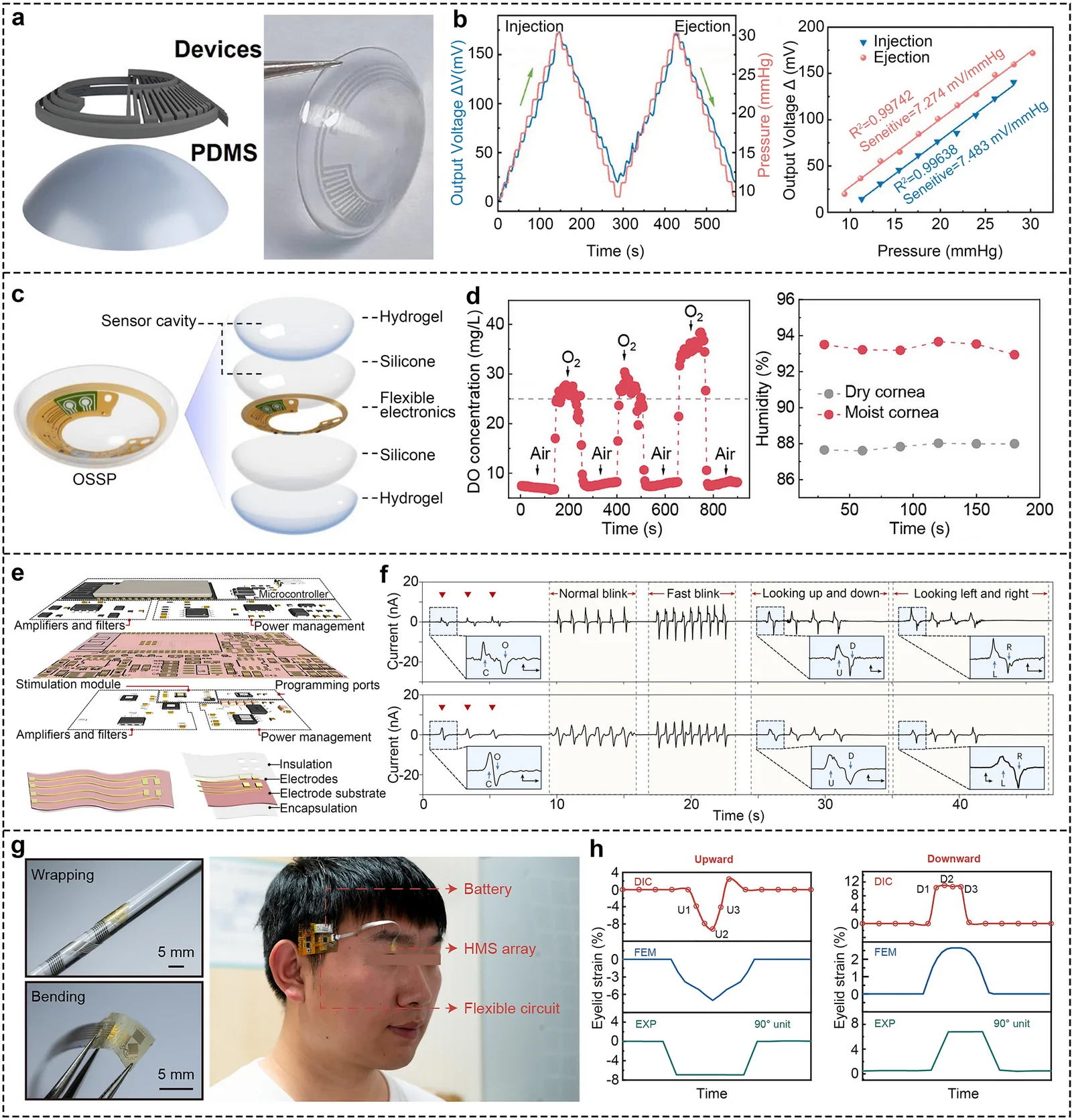
Wearable diagnostic electronics for ocular system. **a** Ti_3_C_2_T_x_ MXene-based soft contact lens for continuous IOP monitoring. **b** Pressure-response characterization on a bionic eyeball platform. Reproduced from Ref. [322] with permission, copyright 2024, Wiley. **c** Kirigami-inspired BSCL for ocular DO and microenvironment monitoring. **d** In vivo DO and humidity tracking in beagle eyes. Reproduced from Ref. [323] with permission, copyright 2025, Wiley. **e** Soft magnetoelastic eyelid sensor for fatigue decoding. **f** Eyelid-generated signals during different ocular activities. Reproduced from Ref. [244] with permission, copyright 2025, Springer Nature. **g** AI-integrated skin-like Eyelectronics platform for strabismus assessment. **h** Eyelid strain analysis using DIC, finite element analysis, and sensor array signals. Reproduced from Ref. [325] with permission, copyright 2026, American Association for the Advancement of Science

The ocular system is uniquely vulnerable to progressive pathological changes that, if left undetected, can culminate in permanent vision impairment. Ophthalmic diseases, including glaucoma, keratitis, and postoperative myopia, represent significant threats to global vision, often requiring continuous monitoring of intraocular pressure (IOP) and the ocular surface microenvironment [380]. Traditional tonometry and clinical examinations provide only intermittent data, failing to capture the dynamic physiological fluctuations essential for early diagnosis. For example, clinic-based IOP measurements can miss circadian IOP peaks, posture-dependent changes, and transient ocular surface abnormalities that occur outside examination windows. To fill this gap, wearable technology has evolved toward ultra-sensitive contact lens sensors, which may complement Goldmann applanation tonometry, rebound tonometry, slit-lamp examination, optical coherence tomography, and visual-field testing by providing continuous ocular physiological trends. For instance, a Ti_3_C_2_T_x_ MXene-based soft contact lens (MX-CLS) was developed for nondestructive IOP monitoring, achieving a high sensitivity (Fig. 19a, b) [322]. Furthermore, a kirigami-inspired breathable smart contact lens (BSCL) enables wireless monitoring of dissolved oxygen (DO), humidity, and temperature (Fig. 19c, d) [323] Nevertheless, current platforms remain at the preclinical stage, with validation limited to artificial eyeball models or animal eyes and no comparative data against Goldmann applanation tonometry or clinical corneal oximetry, leaving their quantitative accuracy and diagnostic equivalence in human subjects yet to be established.

Advancing beyond passive sensing, ocular electronics integrate sensorimotor loops and multi-parameter correction to ensure diagnostic accuracy. Liu et al. [324] reported a neuroprosthetic contact lens (NCL) system that mimics biological feedback by combining a Ti_3_C_2_T_x_ Wheatstone bridge-structured IOP strain sensor with a temperature sensor. Such closed-loop systems facilitate prompt point-of-care treatment feedback for oculopathies while improving the objectivity of clinical assessments. The current era of “Eyelectronics” leverages high-fidelity hardware and AI to automate the diagnosis of complex conditions such as strabismus and fatigue. A self-powered on-eyelid magnetoelastic sensor was designed to capture eye-blink parameters and decode fatigue levels via a one-dimensional convolutional neural network (1D-CNN) (Fig. 19e) [244]. Electrical signals recorded from the upper and lower eyelids during various movements enable robust detection of blinking strength and directional eye movements, as shown in Fig. 19f. To simplify strabismus diagnosis, Yang et al. [325] introduced a skin-like eye-wearable system utilizing a multidirectional strain-sensing array (Fig. 19g). Eyelid strain patterns measured by digital image correlation (DIC) are compared with biomechanical simulations in Fig. 19h. This AI-integrated strategy achieves 96.6% accuracy in classifying ocular motility.

### Skeletal Muscle System

Ranging from age-related sarcopenia to chronic muscle atrophy and neuromuscular disorders, musculoskeletal diseases broadly impair patients’ mobility and quality of life, underscoring the critical need for continuous and objective physiological monitoring [381, 382]. Traditional diagnostic methods often rely on subjective manual palpation or bulky clinical hardware, which are unsuitable for long-term home-care settings. Although magnetic resonance imaging (MRI), computed tomography (CT), dual-energy X-ray absorptiometry (DXA), ultrasound, dynamometry, and clinical EMG provide valuable structural or functional information, they are typically episodic, equipment-dependent, and poorly suited for capturing day-to-day changes in muscle activation, fatigue, rehabilitation progress, or mobility performance outside the clinic. The technological evolution in this field began with high-sensitivity flexible piezoresistive sensors designed to track surface muscle deformation and function. For instance, a porous carbon nanotube/polydimethylsiloxane (CNT/PDMS) sensor was developed for evaluating muscle function and the relative skeletal muscle mass index [327]. Such soft electronics overcome the limitations of rigid clinical tools, providing a cost-effective strategy for daily muscle mass assessment. By enabling repeated monitoring of muscle deformation, pressure distribution, EMG activity, ultrasound-derived architecture, and biochemical markers during natural movement, wearable systems address the need for objective home-based assessment of sarcopenia, neuromuscular deterioration, and rehabilitation response. This foundation in physical sensing paved the way for advanced modalities capable of probing internal structural changes.

**Fig. 20:**
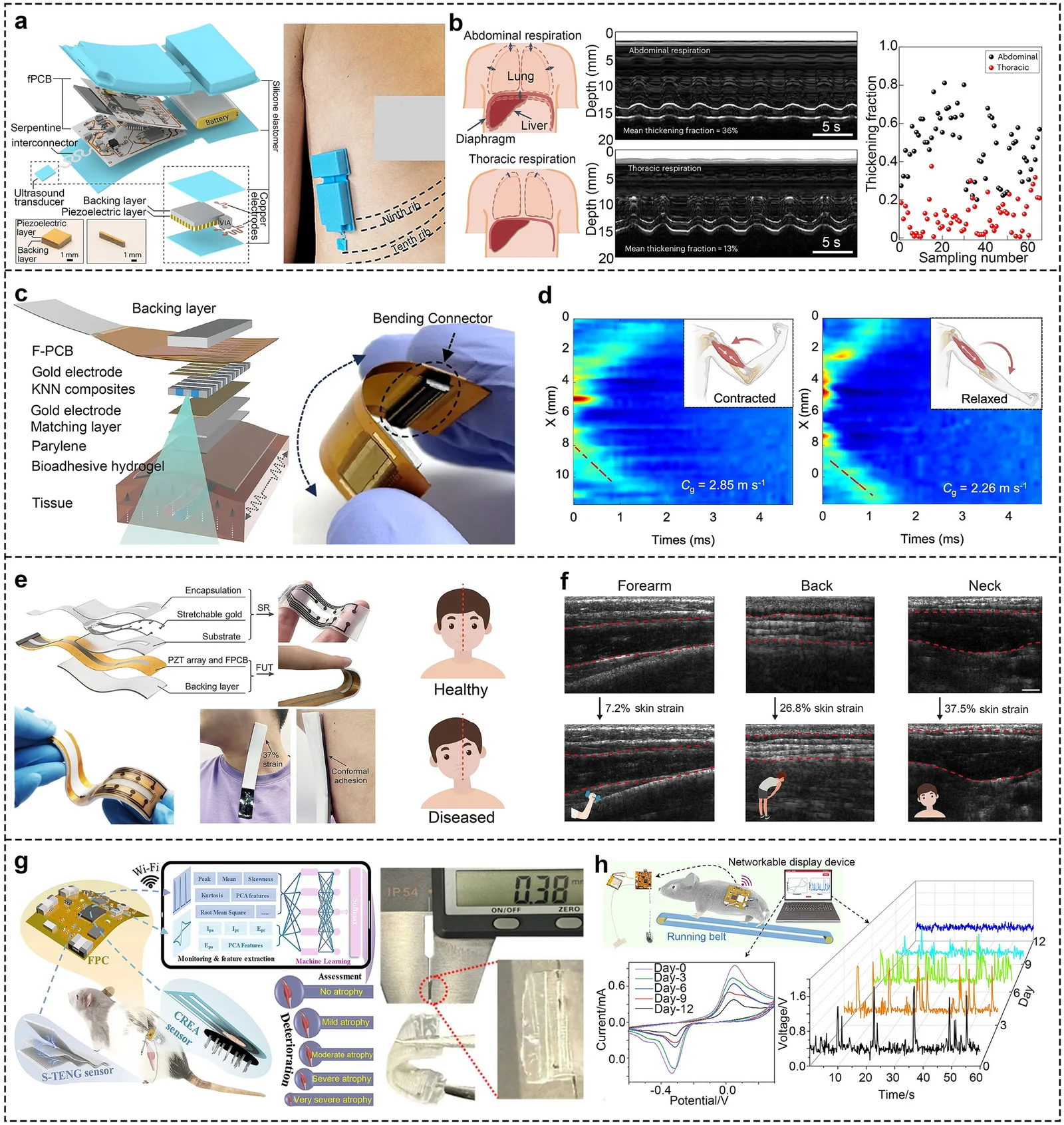
Wearable diagnostic systems for skeletal muscle systems. **a** Single-transducer wearable echomyography system for muscle and respiratory assessment. **b** M-mode ultrasound comparison of abdominal and thoracic breathing. Reproduced from Ref. [329] with permission, copyright 2024, Springer Nature. **c** Lead-free piezoceramics for wearable multimodal ultrasound arrays. **d** Shear wave elastography of the biceps brachii during contraction and relaxation. Reproduced from Ref. [330] with permission, copyright 2025, Springer Nature. **e** Multimodal wearable patch for synchronous structural and functional neuromuscular monitoring. **f** Ultrasound imaging and skin-strain monitoring of dynamic muscle activity. Reproduced from Ref. [331] with permission, copyright 2025, American Association for the Advancement of Science. **g** Wireless multisensor bioelectronic system for muscle atrophy assessment. **h** Rat muscle atrophy evaluation using S-TENG pressure sensing and Cu@SPCE electrochemical signals. Reproduced from Ref. [332] with permission, copyright 2025, American Chemical Society

As clinical needs shifted toward deep-tissue profiling, ultrasonic technologies emerged as a cornerstone for evaluating internal mechanics with high fidelity. A wearable system based on ultrasonic time-of-flight differences was designed to objectively assess soft tissue stiffness by measuring signal shifts under specific pressure gradients [328]. Further optimization led to a single-transducer echomyography (EcMG) system (Fig. 20a), which achieves submillimeter resolution for tracking hand gestures and diaphragm thickness without the need for bulky transducer arrays (Fig. 20b) [329]. To address environmental sustainability and structural flexibility, a 64-channel lead-free ultrasound imaging array (LF-UIA) utilizing potassium sodium niobate-based ceramics was reported (Fig. 20c) [330]. The LF-UIA performs shear wave elastography to quantify the mechanical state of the biceps brachii during dynamic movement (Fig. 20d). For translation, wearable ultrasound systems need validation of imaging depth, spatial resolution, probe-skin coupling stability, and measurement agreement with clinical ultrasound or elastography during both static and dynamic muscle activity.

The current pinnacle of muscle diagnostics involves the fusion of structural, functional, and biochemical data via multimodal systems. A wearable structural–functional sensing patch (WSFP) was developed to synchronously analyze muscle architecture via ultrasound and functional activity through EMG (Fig. 20e) [331]. The WSFP demonstrates the capability to maintain reliable and clear ultrasound imaging of deep muscles even under 37.5% skin surface strain during complex dynamic rotations (Fig. 20f). For comprehensive grading of muscle deterioration, a wireless multisensor wearable system (WMWS) integrates a single-electrode triboelectric nanogenerator (S-TENG) for pressure sensing with an electrochemical creatinine (CREA) sensor (Fig. 20g) [332]. Integrated WMWS monitoring in denervated rats reveals progressively reduced plantar-pressure signals and altered CREA responses as muscular atrophy worsens (Fig. 20h). Although these multimodal platforms provide richer phenotyping of neuromuscular deterioration, their clinical diagnostic value will depend on patient-cohort studies that link wearable readouts to disease severity, rehabilitation outcomes, and treatment-response metrics rather than only animal-model or short-term feasibility data.

### Skin

The skin, as the body’s largest organ and primary interface with the external environment, is susceptible to a broad spectrum of pathological conditions, ranging from chronic inflammatory dermatoses such as atopic dermatitis (AD) and psoriasis to non-healing wounds including diabetic foot ulcers and pressure sores, all of which impose a substantial burden on global healthcare systems [383–386]. Despite their distinct etiologies, these conditions share a critical unmet clinical need: the inability of traditional management strategies, which rely on periodic visual inspections and subjective assessments, to capture the dynamic physiological shifts and subclinical changes that drive disease progression and secondary complications. In routine dermatology and wound care, visual scoring, lesion photography, and bedside inspection are essential but remain observer-dependent, episodic, and often insensitive to early changes in barrier disruption, pressure loading, infection, exudate chemistry, or microenvironmental deterioration. This fundamental limitation has catalyzed a paradigm shift toward continuous, data-driven wearable monitoring platforms that objectively quantify the skin’s physiological and biomechanical properties in real time. By tracking hydration, transepidermal water loss (TEWL), pressure, temperature, pH, impedance, inflammatory mediators, and wound metabolites, wearable skin systems can provide early warning of flare, infection, pressure injury, delayed healing, and support more individualized treatment adjustment.

**Fig. 21:**
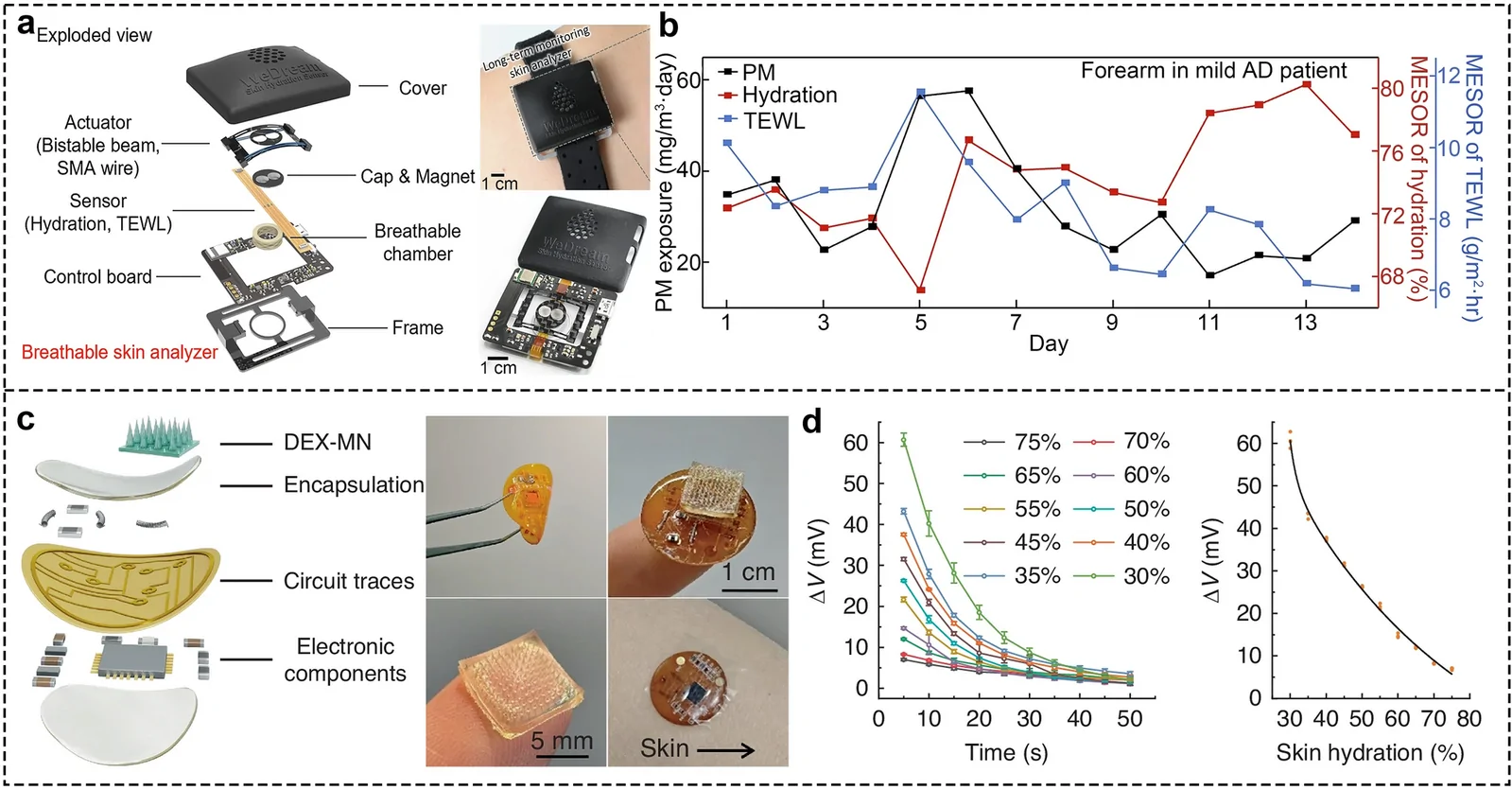
Wearable diagnostic systems for skin diseases. **a** Breathable wearable skin analyzer for long-term barrier-function monitoring. **b** Longitudinal hydration and TEWL assessment in AD patients under PM exposure. Reproduced from Ref. [335] with permission, copyright 2025, Springer Nature. **c** Self-powered skin patch integrating hydration monitoring and on-demand drug delivery for AD. **d** Hydration-sensing performance in porcine skin samples. Reproduced from Ref. [336] with permission, copyright 2025, Springer Nature

Wireless skin-interfaced devices enable noninvasive, conformal coupling with targeted anatomical regions of the body, facilitating the continuous monitoring of skin conditions. For instance, a battery-free, wireless soft sensor system was developed to provide simultaneous, multi-site measurements of pressure and temperature for patients at risk for pressure injuries [334]. Building on the necessity for barrier-function analysis, a breathable skin health analyzer was introduced for the long-term, continuous monitoring of skin hydration and TEWL (Fig. 21a) [335]. The natural circadian fluctuations and the impact of particulate matter (PM) on skin condition are captured using midline estimating statistics of rhythm analysis as shown in Fig. 21b. This dual-parameter approach avoids reliance on single-metric assessments, providing a more accurate diagnosis of barrier disruption in AD patients. To bridge the gap between diagnosis and treatment, a self-powered, closed-loop skin patch for AD was proposed that integrates a piezoelectric generator with a hydration sensing unit (Fig. 21c) [336]. The corresponding fitting curve in Fig. 21d proves that higher hydration facilitates faster thermal recovery, enabling the automatic activation of drug-loaded MNs. These examples indicate that wearable skin platforms can complement clinical inspection by quantifying barrier function, pressure exposure, and treatment response, but they still require validation against standardized skin instruments and longitudinal outcomes in diverse patient populations.

Recent advancements in living electronics seamlessly integrate synthetic materials with biological entities to modulate the immune environment. For instance, an active biointegrated living electronics platform utilizes a Staphylococcus epidermidis–laden hydrogel to provide microbial-driven intervention for psoriasis [337]. This system simultaneously monitors skin impedance, temperature, and humidity, showing a constant decrease in impedance that aligns with the recovery of the psoriasis severity index. Because living or drug-delivering skin electronics introduce additional biosafety and regulatory considerations, preclinical and clinical evaluation must systematically address microbial containment integrity, infection, and sensitization risk.

**Fig. 22:**
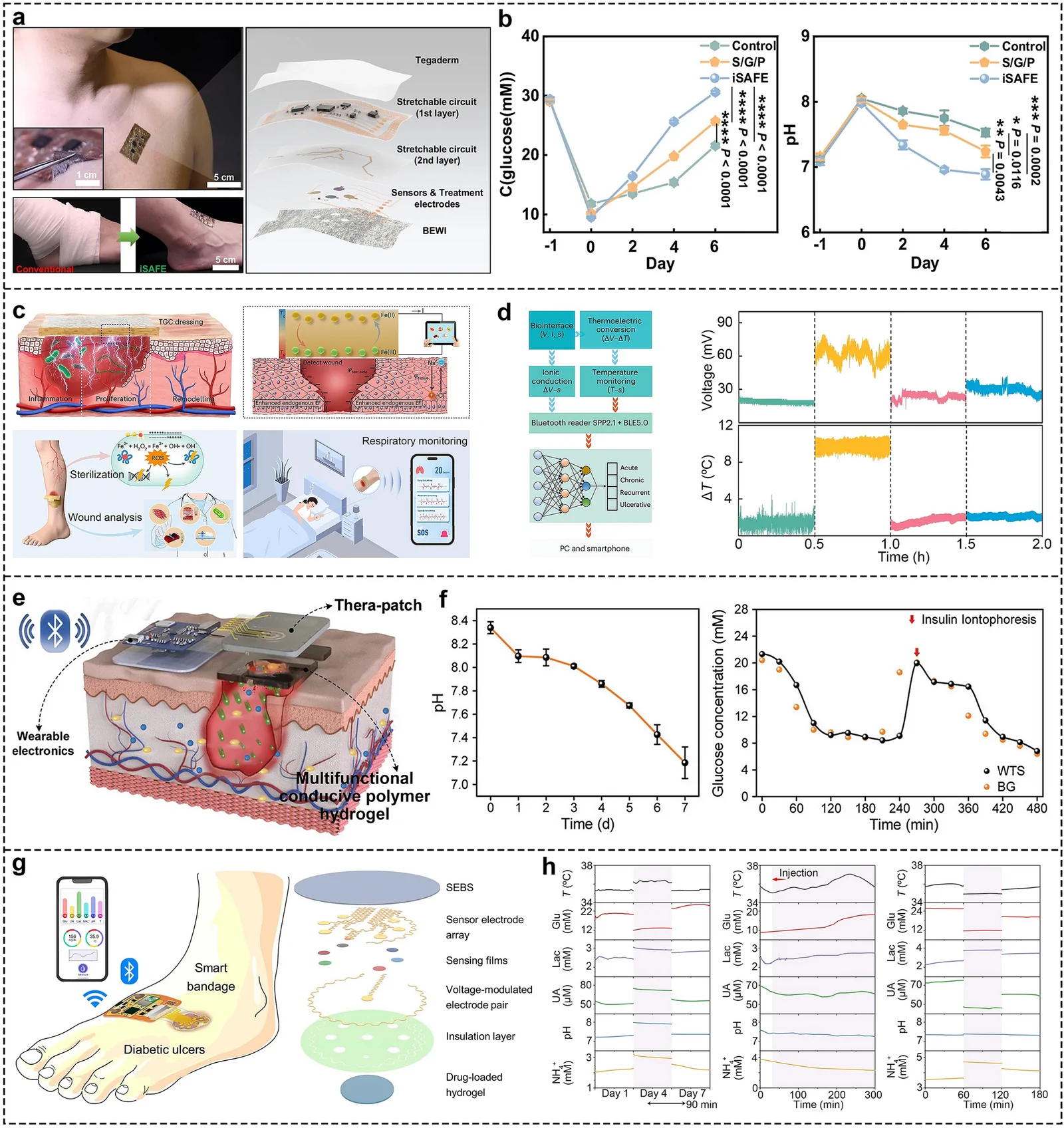
Wearable diagnostic systems for wound monitoring. **a** Skin-interfaced 3D closed-loop electronic bandage for sensing and therapy. **b** In vivo glucose and pH monitoring in infected diabetic wounds using iSAFE. Reproduced from Ref. [232] with permission, copyright 2025, Springer Nature. **c** Smart thermogalvanic cell (TGC) dressing for energy harvesting and wound monitoring. **d** Temperature-gradient, voltage, and current characterization of the TGC dressing in porcine wounds. Reproduced from Ref. [340] with permission, copyright 2026, Springer Nature. **e** Hydrogel-based theranostic platform for chronic diabetic wound management. **f** Glucose tracking and wound-healing assessment with an iontophoresis-enhanced patch. Reproduced from Ref. [113] with permission, copyright 2024, Wiley. **g** Stretchable wireless bioelectronic system for multiplexed wound-fluid analysis and therapy. **h** In vivo monitoring of wound-fluid metabolites in diabetic mice. Reproduced from Ref. [342] with permission, copyright 2023, American Association for the Advancement of Science

Smart bandages provide continuous, real-time insights into the wound microenvironment. This capability addresses a major limitation of conventional wound care, in which dressing changes, visual inspection, swab culture, and wound-size measurement provide delayed and intermittent information and may miss early infection, ischemia, excessive pressure, or biochemical deterioration before visible worsening occurs. Early advancements in this field focused on integrating flexible sensors to track fundamental parameters such as pH, temperature, and moisture. For instance, Huang et al. [232] reported a battery-free, fully permeable, skin-adhesive, stretchable electronic wound bandage (iSAFE) capable of in situ assessment of glucose and pH levels (Fig. 22a). The declining trends of pH and glucose in infected wounds confirmed the system’s ability to track infection clearance and healing progress, as illustrated in Fig. 22b. Similarly, the VeCare platform was developed for the multiplexed immunosensing of inflammatory mediators, providing a holistic profile of the wound’s infection state without bulky laboratory equipment [338]. Clinically, these biochemical and physical readouts should be validated against wound-area reduction, exudate analysis, microbial culture, inflammatory markers, histological assessment when available, and standardized wound scores, so that sensor outputs can be linked to infection status and healing trajectory rather than isolated biomarker fluctuations.

Beyond surface contact monitoring, the detection of gaseous molecular fluxes offers deeper insights into cutaneous homeostasis. A significant breakthrough in this area is a non-contact wearable platform that defines a microclimate adjacent to the skin to monitor epidermal molecular flux [339]. By utilizing a programmable bistable valve and wireless sensors, this device quantitatively measures the inward and outward fluxes of water vapor, CO_2_, and VOCs. This non-contact proximity operation is particularly advantageous for fragile wound tissues, as it avoids mechanical damage while identifying characteristic flux variations linked to pathologies and infections. Complementing these molecular insights, self-powered sensing has emerged to simplify device architectures. Xin et al. [340] designed a thermogalvanic cell (TGC) dressing that converts the temperature gradient between the wound and the environment into an exogenous electric field (Fig. 22c). The output voltage reached 60–80 mV during the peak inflammatory phase, enabling autonomous wound stage analysis without external power (Fig. 22d).

The field has recently progressed toward active intervention through “theranostic” platforms that respond dynamically to the evolving needs of the wound bed. These systems integrate stimulators to deliver programmed electrical cues or on-demand drug release based on real-time sensor feedback. For example, a wireless smart bandage was engineered with hydrogel electrodes to monitor skin impedance while delivering directional electrical stimulation, resulting in a 25% increase in healing speed [59]. Additionally, a battery-free NFC-integrated dressing was developed to monitor UA and pH, simultaneously triggering the release of cefazolin to inhibit bacterial growth [343]. Gong et al. [113] presented a wireless theranostic system that utilizes electro-responsive hydrogels for closed-loop insulin delivery and pH monitoring (Fig. 22e). Figure 22f demonstrates that iontophoresis-driven insulin delivery rapidly stabilizes wound glucose levels within the normal range compared to free diffusion controls. Furthermore, through the integration of AI, the a-Heal platform integrates an “ML Physician” that utilizes image recognition to diagnose wound stages and prescribe ad hoc bioelectronic therapies [341]. To enhance diagnostic depth, Sani et al. [342] introduced a multiplexed patch for the spatial and temporal analysis of ammonium, lactate, and glucose (Fig. 22g). The elevated UA and decreased glucose levels during infection were tracked successfully, demonstrating bacterial elimination following the initiated treatment, as shown in Fig. 22h.

### Oncology

**Fig. 23:**
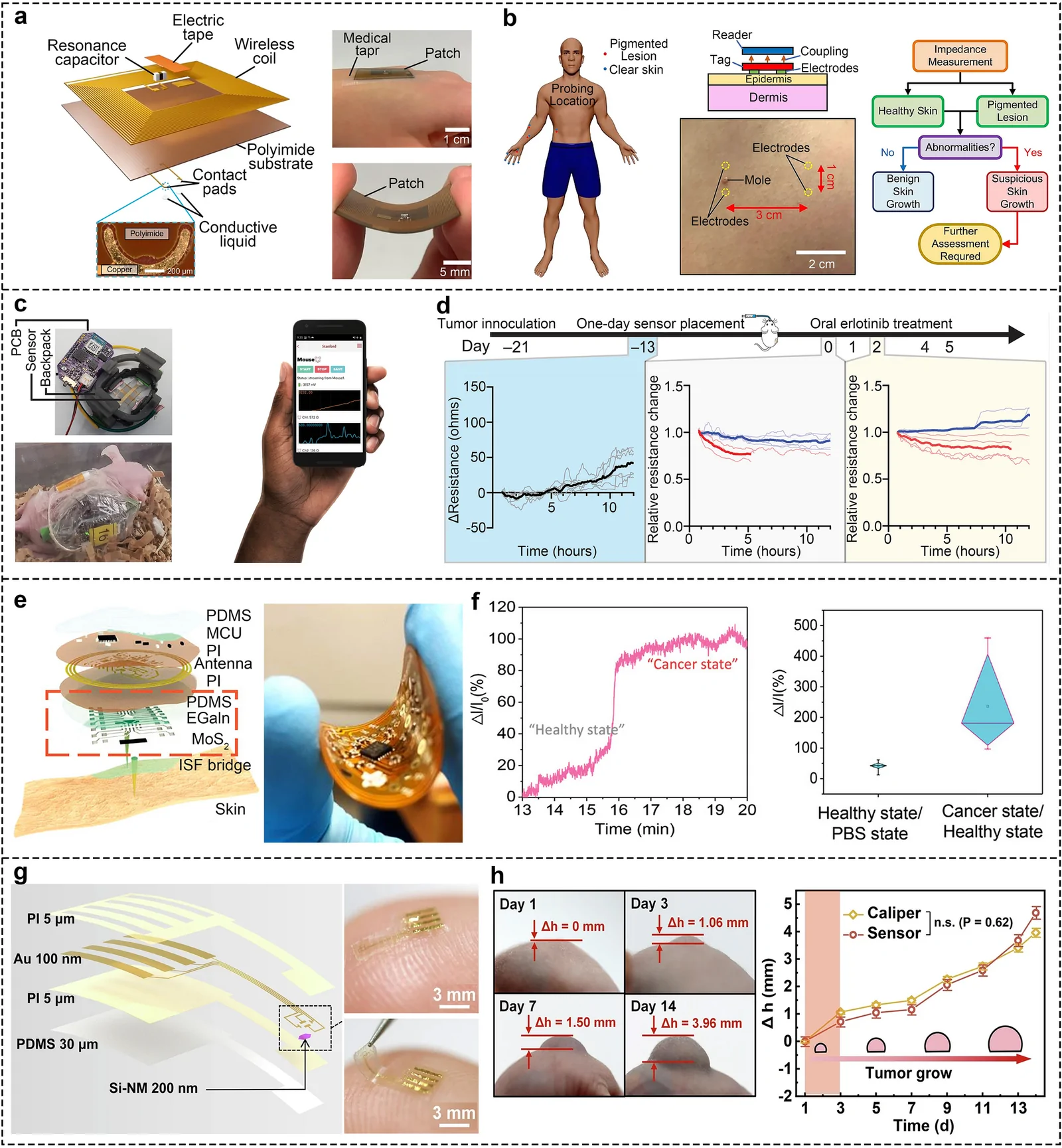
Wearable diagnostic systems for oncology. **a** Battery-free chipless patch for bioimpedance measurement of cutaneous lesions. **b** Human bioimpedance assessment of moles and healthy skin. Reproduced from Ref. [345] with permission, copyright 2025, Springer Nature. **c** Flexible strain sensor for autonomous tumor-regression monitoring. **d** Continuous tumor volume tracking during erlotinib treatment in HCC827 mouse models. Reproduced from Ref. [347] with permission, copyright 2022, American Association for the Advancement of Science. **e** Self-healing electronic platform for methylated ctDNA detection. **f** In vivo ctDNA monitoring using the iMethy device. Reproduced from Ref. [348] with permission, copyright 2023, Wiley. **g** Conformal silicon Hall-sensor arrays with DL for 3D tumor-growth monitoring. **h** Longitudinal tumor-height comparison between flexible Hall sensors and caliper measurements. Reproduced from Ref. [350] with permission, copyright 2025, Springer Nature

Cancer remains a leading cause of global mortality, often diagnosed at advanced stages because early lesions can be clinically silent and because current screening and follow-up still depend largely on scheduled imaging, tissue biopsy, and laboratory molecular tests. Early detection and continuous tracking of tumor dynamics are essential for improving prognosis and assessing therapeutic efficacy. In this context, wearable oncology devices are more realistically positioned as complementary tools for longitudinal surveillance, treatment-response monitoring, and early warning rather than standalone replacements for histopathology, radiological imaging, or clinically validated molecular diagnostics. Tissue biopsy remains the diagnostic gold standard because it provides histopathological and molecular information, but it is invasive, difficult to repeat frequently, subject to spatial sampling bias, and usually represents only a single lesion at a single time point. Imaging methods provide essential anatomical or metabolic assessment but are scheduled, costly, and limited in temporal resolution. Plasma circulating tumor DNA (ctDNA) analysis has expanded noninvasive molecular monitoring, yet it still requires blood collection and laboratory processing, and its sensitivity can be limited in early-stage or low-burden disease. Initial wearable breakthroughs focused on monitoring biophysical changes such as tumor volume and tissue impedance. Imtiaz et al. [345] developed a battery-free, wireless patch to measure the bioimpedance of melanoma lesions, alerting users to abnormal shifts in resonance frequency that signify malignant development (Fig. 23 a, b). To track mechanical regression, a hafnium oxide (HfO_2_) NP-embedded strain sensor was designed to monitor tumor volume changes and initiate targeted sonodynamic therapy [346]. Furthermore, the Flexible Autonomous Sensor measuring Tumor volume regression (FAST) system was introduced to track micrometer-scale changes at 5-min intervals (Fig. 23c) [347]. Figure 23d demonstrates how the FAST sensor identifies tumor regression within 5 h of therapy initiation. The continuous data stream allows for rapid therapeutic screening, improving temporal resolution relative to conventional caliper-based measurements. The value of wearable oncology platforms therefore lies in increasing sampling frequency and reducing monitoring burden, enabling earlier detection of local growth, regression, drug-response trends, or recurrence-related signals between scheduled hospital visits.

The capture of specific circulating biomarkers in biofluids, often termed “wearable liquid biopsy,” provides molecular-level insights into tumor progression. An integrated bioelectronic patch, termed iMethy, was developed for the dynamic surveillance of methylated ctDNA in ISF (Fig. 23e) [348]. This platform utilizes a self-healing FET circuit to achieve a detection limit of 10^–16^ M. In vivo quantification of the source–drain current (*I*_DS_) shows a distinct change in the cancerous state compared to healthy baselines (Fig. 23f). These results highlight the effectiveness of iMethy for noninvasive cancer risk management. Additionally, printable core–shell NPs were synthesized with a molecularly imprinted polymer shell to enable customizable recognition of anticancer drugs [215]. By quantifying redox signals via differential pulse voltammetry, these sensors facilitate real-time therapeutic drug monitoring for drugs like busulfan, ensuring personalized dosage and minimized toxicity. However, wearable liquid-biopsy strategies must establish robust correlations between ISF, sweat, or other accessible biofluids and clinically accepted plasma ctDNA, tissue mutation status, or therapeutic drug monitoring assays.

Noninvasive chemical sensing of VOCs offers a promising avenue for cancer detection, as these molecules serve as metabolic “fingerprints” characteristic of malignant cellular activity. Given that aberrant tumor metabolism generates distinctive gaseous by-products, electronic nose (e-nose) platforms capable of detecting such compounds in exhaled breath or sweat hold considerable potential for ultra-early oncological screening. Chen et al. [349] developed a hydrogel-based SERS sensor to monitor lung cancer treatment effects through sweat carbonyl biomarkers. The final maturity of oncology platforms involves the synergy between high-sensitivity sensors and DL architectures. A conformal Hall-sensor array was integrated with CNNs to achieve precise 3D profiling of early-stage tumors (Fig. 23g) [350]. The Hall-sensor platform tracks submillimeter height variations during the first three days of growth, which are otherwise undetectable by visual inspection (Fig. 23h).

### Diabetes

**Fig. 24:**
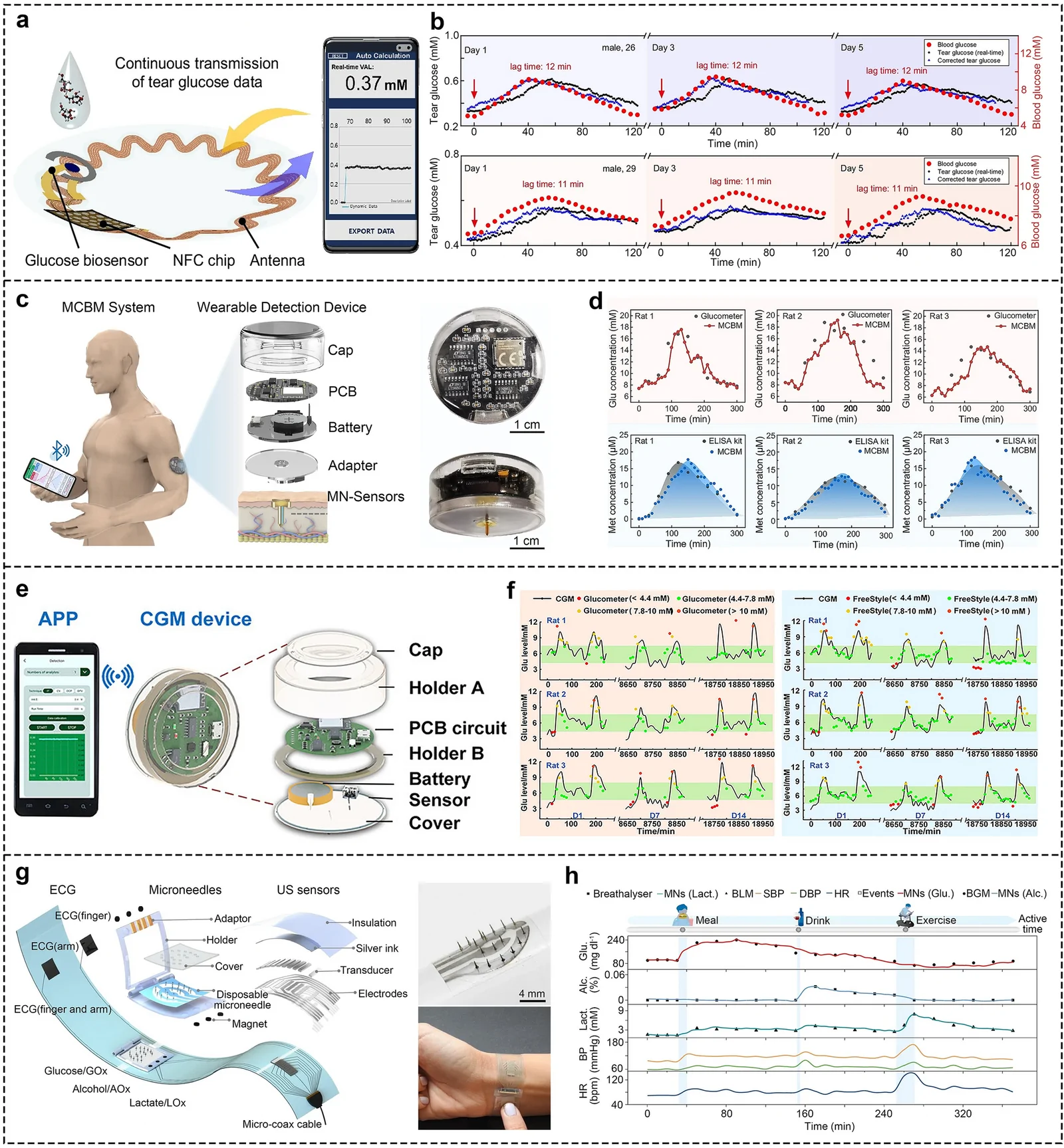
Wearable diagnostic systems for diabetes monitoring. **a** Wireless smart contact lens for tear-glucose and blood glucose correlation analysis. **b** Longitudinal tear and blood glucose monitoring after beverage intake. Reproduced from Ref. [352] with permission, copyright 2024, Springer Nature. **c** MN-based platform for pharmacokinetic/pharmacodynamic evaluation. **d** Glucose and metformin monitoring using the MCBM system with reference validation. Reproduced from Ref. [219] with permission, copyright 2025, Springer Nature. **e** Smartphone-controlled MN CGM system for home-care diabetes management. **f** Fourteen-day ISF glucose monitoring compared with commercial references. Reproduced from Ref. [112] with permission, copyright 2023, American Chemical Society. **g** Integrated wearable platform for simultaneous chemical and cardiovascular monitoring. **h** Multimodal monitoring of ISF glucose, lactate, alcohol, BP, and HR during meals and exercise. Reproduced from Ref. [60] with permission, copyright 2025, Springer Nature

Effective management of diabetes mellitus requires maintaining blood glucose levels within a narrow physiological range to prevent debilitating complications such as neuropathy and retinopathy [387]. For decades, standard glycemic assessment has relied on finger-prick self-monitoring, venous plasma glucose, hemoglobin A1c (HbA1c), and oral glucose tolerance testing, which are invasive, episodic, or unable to provide continuous information. This approach fails to reflect the continuous “peaks and valleys” of glucose fluctuations, often missing postprandial spikes or dangerous nocturnal hypoglycemia. HbA1c further provides only an integrated retrospective estimate of glycemic exposure and cannot reveal glucose variability, time in range, or acute hypoglycemic risk. Wearable continuous glucose monitoring (CGM) systems have revolutionized this paradigm by providing real-time glycemic trends via noninvasive or minimally invasive sensing. By establishing a 24-h glucose profile, these devices can alert users to rapid fluctuations as they happen, allowing for immediate lifestyle or insulin adjustments and improving glycemic control beyond what snapshot testing alone can achieve. The key clinical need addressed by wearable glucose systems is therefore continuous trend awareness, including detection of nocturnal hypoglycemia, postprandial hyperglycemia, exercise-associated fluctuations, and medication-related glucose responses in daily life. Park et al. [352] presented a wireless, soft smart contact lens designed to quantitatively monitor glucose levels in basal tears while excluding reflex tear interference (Fig. 24a). Figure 24b reveals a strong correlation between tear and blood glucose levels following sugar intake, demonstrating the “personalized lag time” concept for accurate glycemic prediction. Additionally, a multifunctional contact lens was developed for simultaneous sensing and electrically controlled drug delivery to treat diabetic retinopathy [353]. Transitioning to other biofluids, Zhang et al. [354] reported an integrated paper-based patch (IPP) that utilizes conductive ink and iontophoresis to enable the highly sensitive, noninvasive monitoring of sweat glucose and other renal biomarkers at rest.

To address the physiological lag associated with noninvasive fluids, the minimally invasive platforms have been developed to access ISF. Cheng et al. [355] developed a touch-actuated biosensor combining solid MNs for painless penetration with reverse iontophoresis, effectively increasing glucose extraction flux. Building on this, Yang et al. introduced a wearable MN-based continuous biomarker/drug monitoring (MCBM) system for the simultaneous in vivo pharmacokinetic (PK) and pharmacodynamic (PD) evaluation of glucose and metformin (Fig. 24c) [219]. Figure 24d demonstrates the high correlation between ISF and blood glucose trends alongside the established PK characteristic curve for metformin distribution and elimination stages in rat models. Furthermore, a wearable CGM system integrated with hollow MNs and a sandwich-type enzyme immobilization strategy was developed for cost-effective home-care management (Fig. 24e) [112]. Fourteen-day dynamic glucose tracking in healthy rats demonstrated excellent agreement with commercial glucometers and the ability to monitor rapid fluctuations after glucose injection, as shown in Fig. 24f. These MN-based strategies offer a robust bridge between blood and wearable sensing by minimizing patient discomfort while maximizing signal reliability, providing an ideal foundation for long-term glycemic surveillance in ambulatory settings. To move toward clinical use, these devices should be compared with commercial CGMs and blood glucose references using metrics such as mean absolute relative difference (MARD), error-grid analysis, hypoglycemia detection sensitivity, calibration stability, and long-term skin tolerability.

Beyond biological enzymes, the field has evolved toward non-enzymatic materials and multimodal systems to enhance sensing stability and diagnostic breadth. A self-powered sensor utilizing Pt single-atom bridged nanozymes was designed to oxidize glucose through a direct electron transfer pathway, ensuring high stability in complex biofluids [356]. Similarly, an enzyme-free sweat patch was engineered with a Tesla valve to manage an in situ alkaline environment for nickel-based organic framework nanoflowers [388]. To provide a holistic health assessment, Chang et al. [60] described a hybrid wristband platform integrating MN sensors with ultrasonic arrays for the simultaneous detection of metabolites and cardiac signals (Fig. 24g). The influence of daily activities on glucose and blood pressure was tracked in high-risk prediabetic participants, revealing complex correlations between chemical biomarkers and physiological parameters (Fig. 24h).

Currently, the integration of AI for predictive analytics and closed-loop therapy has been applied in diabetic management. Sun et al. [357] reported a multiplexed MN patch that utilizes a multi-task DL algorithm to distinguish metabolic disorders from normal conditions with an accuracy of 0.996. To mitigate the inherent safety risks of automated insulin delivery, a dual closed-loop system was developed that synergistically combines CGM-controlled delivery with glucose-responsive insulin release [358]. Glycemic profiling in diabetic rat models revealed that this dual-loop strategy significantly prolongs normoglycemia duration and attenuates glycemic fluctuations relative to conventional single-loop systems. These AI-driven advancements represent a clear trajectory toward fully autonomous “digital pancreases,” effectively bridging the gap between diagnosis and real-time, precision intervention for metabolic healthcare.

### Inflammation and Immune Response

**Fig. 25:**
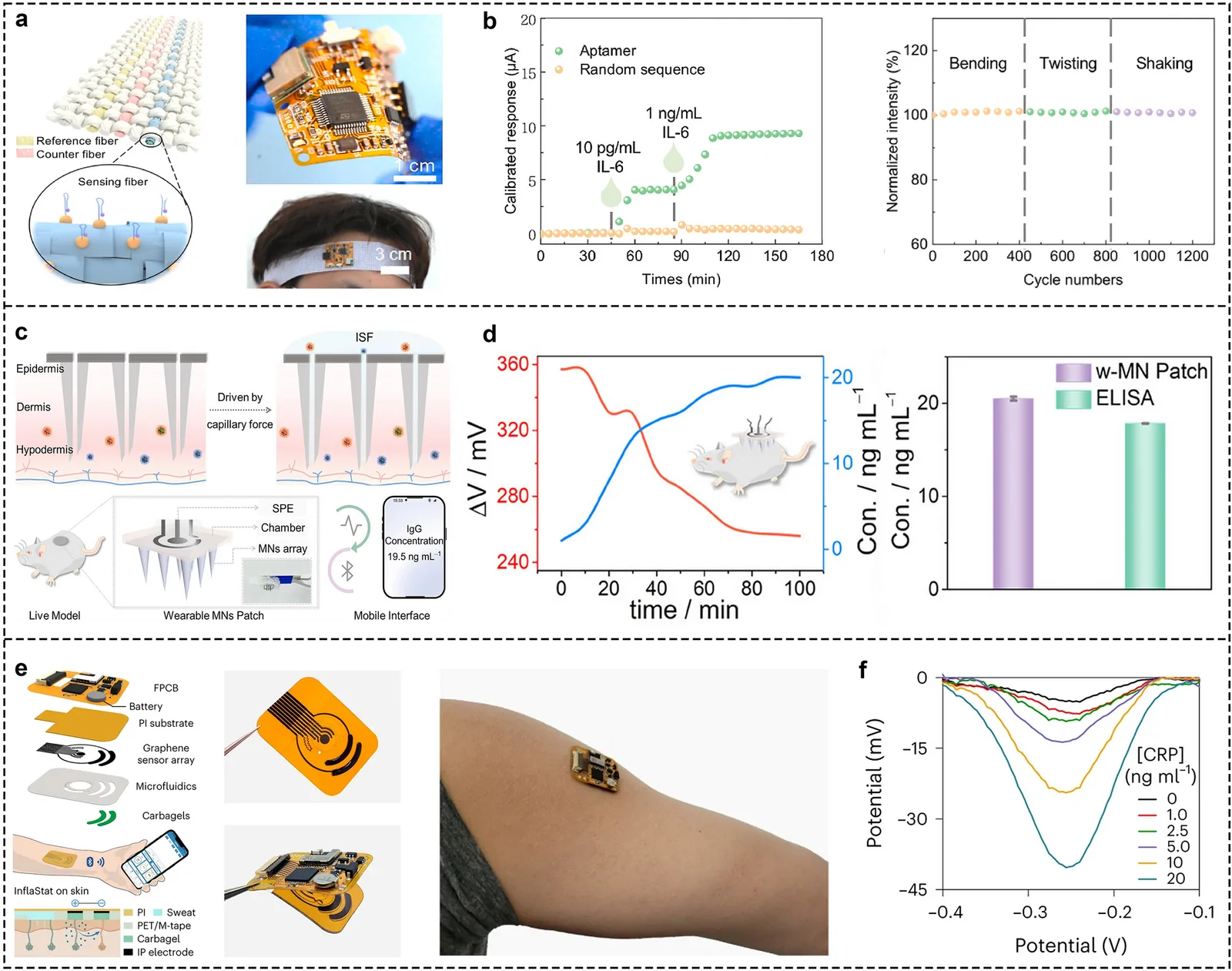
Wearable diagnostic systems for inflammation and immune response diagnosis and monitoring. **a** Electrochemical fabric platform for cytokine monitoring. **b** IL-6 aptamer-fabric response and mechanical stability tests. Reproduced from Ref. [360] with permission, copyright 2023, Elsevier BV. **c** MN patch with metal hydrogel probes for dermal ISF protein monitoring. **d** In vivo IgG sensing in ISF validated against commercial ELISA. Reproduced from Ref. [362] with permission, copyright 2026, Wiley. **e** Wireless bioelectronic patch for sweat CRP monitoring. **f** On-body evaluation of the multiplexed inflammation-monitoring patch. Reproduced from Ref. [359] with permission, copyright 2023, Springer Nature

Inflammation and immune responses are mediated by macromolecular biomarkers such as cytokines (e.g., interleukin-6 (IL-6)) and C-reactive protein (CRP), which are critical for diagnosing sepsis, chronic infections, and autoimmune disorders [389, 390]. Monitoring these molecules is traditionally challenging due to their exceptionally low concentrations in biofluids and large molecular sizes, which hinder rapid diffusion and sensing. Conventional laboratory methods, such as enzyme-linked immunosorbent assay (ELISA), chemiluminescent immunoassays, and blood-based clinical CRP or cytokine panels, are episodic and labor-intensive [359], highlighting the urgent need for real-time, wearable diagnostics to capture dynamic inflammatory fluctuations. These laboratory tests provide reliable quantitative results but are poorly suited for tracking rapid cytokine changes, flare onset, treatment response, or infection progression between hospital visits, especially in chronic inflammatory diseases or decentralized care settings. Initial advancements in this field focused on flexible substrates and highly stable recognition elements. For instance, Chu et al. [360] developed an electrochemical fabric utilizing aptamer-functionalized carbon nanotube/graphene fibers for in situ IL-6 monitoring in sweat (Fig. 25a). The fabric exhibited an obvious response to target cytokines and maintained stable signal retention under repeated mechanical deformations, as shown in Fig. 25b. Such textile-based platforms effectively transform common garments into diagnostic tools, enabling continuous health tracking without compromising user comfort or breathability.

To access biomarkers with higher clinical correlation than sweat, technology evolved toward minimally invasive MN arrays for ISF and dermal analysis. A functional carbon nanotube-based MN patch was engineered to capture cytokines in vivo, providing an early warning system for life-threatening “cytokine storms” within hours [361]. Similarly, Chen et al. reported a fully integrated wearable MN patch incorporating a metal hydrogel-based electrochemical probe for monitoring immunoglobulin G (IgG) (Fig. 25c, d) [362]. The output signals correlate well with commercial ELISA kits, confirming the device’s high reliability for assessing immune disorders. Beyond mechanical extraction, a wearable photonic device was developed to induce blood biomarker extravasation via light-absorbing hemoglobin [363]. This optical prototype enables the detection of multiple biomarkers like CRP and IL-6 without traditional blood draws, bypassing epidermal variations and significantly enhancing sensitivity for low-abundance proteins.

The integration of advanced microfluidics and AI represents the current frontier of inflammatory surveillance and personalized healthcare. For instance, a wearable sweat sensing patch (WSSP) was developed to monitor CRP through lateral flow biosensing, utilizing ML algorithms for colorimetric signal interpretation [364]. This intelligent platform achieves 100% classification accuracy, bridging the gap between simple on-body tests and precise clinical diagnostics. Furthermore, the development of "InflaStat" by Tu et al. [359] enabled the real-time detection of CRP in patients with COPD and COVID-19, facilitating decentralized disease management with high sensitivity and effectiveness (Fig. 25e, f). Ultimately, the future of this field lies in leveraging big data and AI-driven predictive modeling to establish personalized immune “digital twins.” By integrating multiplexed sensing of diverse inflammatory cascades, wearable electronics will move toward proactive decision-making and the precise management of complex systemic immune disorders.

### Other Biomedical Applications

**Fig. 26:**
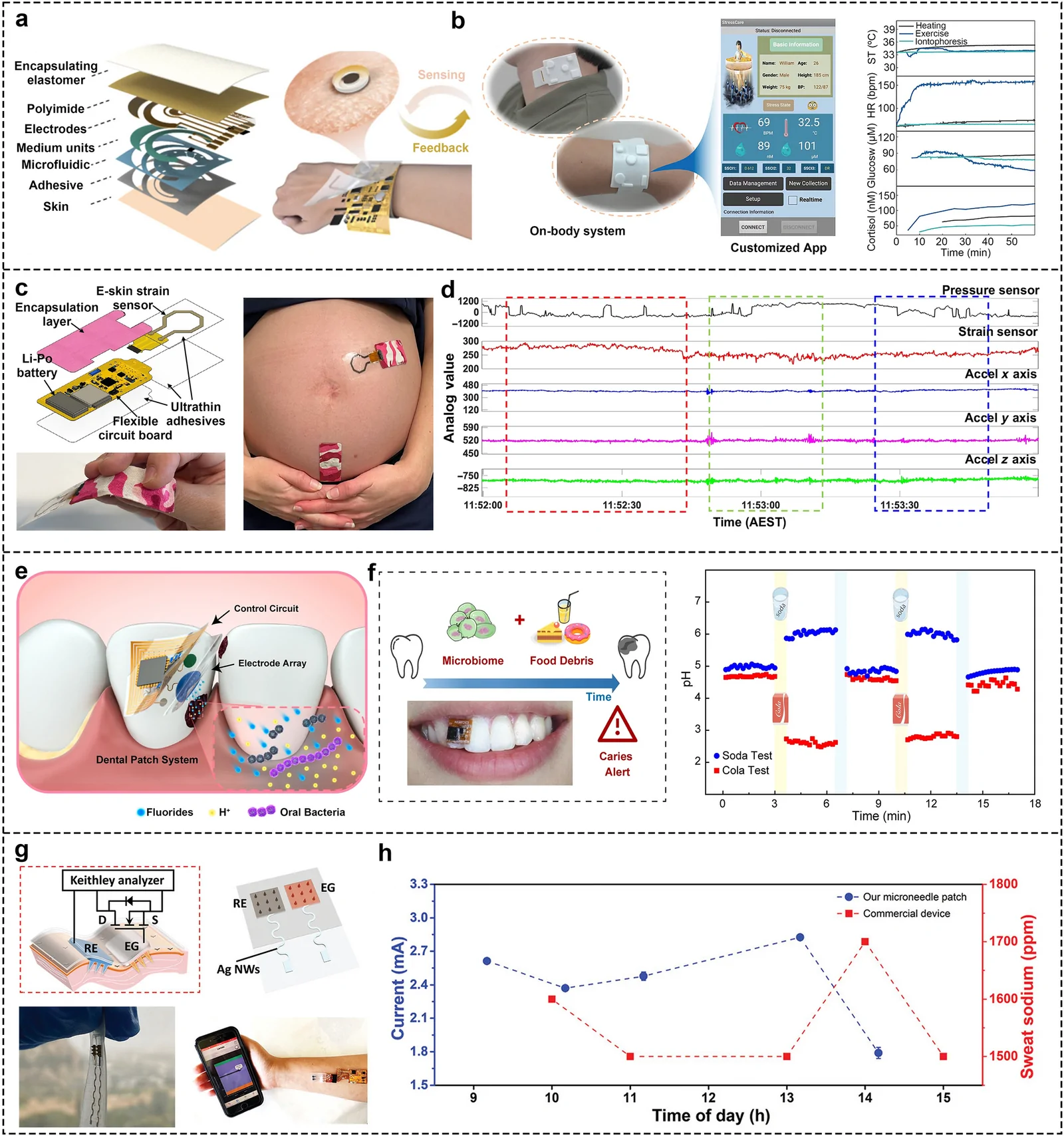
Wearable diagnostic systems for other clinical applications. **a** FIRES platform for acute and chronic stress assessment. **b** Wearable FIRES device and real-time biomarker monitoring during thermal, exercise, and iontophoresis stimuli. Reproduced from Ref. [365] with permission, copyright 2025, American Chemical Society. **c** Compact pressure-strain sensor system for fetal-movement monitoring. **d** Multimodal pressure, strain, and acceleration signals during fetal-movement detection. Reproduced from Ref. [366] with permission, copyright 2025, American Association for the Advancement of Science. **e** Battery-free theranostic dental patch for intraoral pH sensing and on-demand fluoride delivery. **f** Real-time intraoral pH monitoring after beverage intake. Reproduced from Ref. [367] with permission, copyright 2022, Springer Nature. **g** MN-EGFET patch for ISF Na^+^ detection. **h** On-body ISF Na^+^ monitoring compared with sweat-based devices. Reproduced from Ref. [368] with permission, copyright 2022, Wiley

Mental health conditions, particularly stress, are paramount concerns impacting global well-being, yet clinical evaluation remains reliant on subjective psychometric tools. Although questionnaires and clinician interviews are indispensable, they are episodic, recall-dependent, and limited in their ability to capture context-dependent stress responses during work, sleep, exercise, or social interaction. To address this, smart quantitative and comprehensive stress assessor and sub-classifier (SQC-SAS) systems have been developed to simultaneously measure molecular stress biomarkers and physiological stress indicators [391]. This nanomaterial-based wearable enables data-driven sub-classification of stress states through integrated ML models. Similarly, a fully integrated reusable electronic system (FIRES) was introduced by Chu to build a monitoring panel for cortisol, glucose, skin temperature, and heart rate (Fig. 26a) [365]. A BLE-connected mobile application enables long-term health management, while the wearable device maintains stable epidermal conformability during daily activities, supporting objective and scalable mental healthcare assessment (Fig. 26b). The value of such platforms lies in providing longitudinal physiological context for stress-related states rather than replacing psychiatric diagnosis. For future development, stress-related wearable outputs should be compared with validated psychometric scales, clinician-administered assessments, salivary or serum cortisol assays, sleep/activity records, and longitudinal symptom changes, because physiological arousal alone is not specific to psychiatric stress.

Personalized reproductive monitoring and fetal well-being are critical emerging fields where noninvasive wearables provide alternatives to invasive blood draws or intermittent ultrasound. Current hormone testing and obstetric assessment are usually clinic- or laboratory-dependent and provide limited temporal resolution, making it difficult to follow cycle dynamics, ovulation-related changes, or fetal-movement patterns continuously at home. An aptamer-based nanobiosensor was reported for the autonomous, real-time monitoring of estradiol in sweat with sub-picomolar sensitivity [76]. This integrated system employs target-induced strand displacement and deoxyribonucleic acid (DNA) aptamers to enable reagentless hormone tracking during menstrual cycles. In parallel, fetus health can be tracked using a compact pressure-strain combo sensor system (Fig. 26c) [366]. Synchronized time-series signals from pressure and strain sensors track abdominal motions (Fig. 26d). The pressure patch effectively differentiates between localized fetal twitching and nonlocalized kicking events. Supported by an AI-powered framework, this Band-Aid-like device achieves over 90% accuracy in discriminating fetal movements from maternal activities. These technologies empower women with convenient, out-of-hospital reproductive health management.

Beyond systemic health, localized oral environments and specific prognostic biomarkers like Na^+^ are gaining attention in flexible bioelectronics. In oral health and electrolyte management, conventional dental examinations, salivary tests, and blood electrolyte measurements are intermittent and often miss rapid changes caused by diet, microbial metabolism, hydration, exercise, or renal dysfunction. A battery-free dental patch was developed to monitor topical acidic fluctuations caused by microbial metabolism and deliver fluoride on-demand via NFC (Fig. 26e) [367]. Intraoral pH fluctuations recorded during beverage consumption demonstrate the system’s accuracy. Topical pH values decrease below 3.0 after cola intake and recover following water rinsing (Fig. 26f). Furthermore, Zheng et al. [368] developed a stretchable MN-based extended-gate field-effect transistor (MN-EGFET) for the continuous detection of Na^+^ in ISF (Fig. 26g). Sodium concentrations in the ISF are tracked over several hours (Fig. 26h). These fluctuations exhibit a similar tendency to sweat levels but occur with a noticeable time lag. This platform integrates with IoT clouds platforms, providing new opportunities for efficient medical care and accurate clinical decision-making. These innovations illustrate the potential of wearables to cover diverse pathological parameters. The emergence of POCT and flexible bioelectronics marks a shift toward decentralized, continuous healthcare. Integrating multimodal sensors with AI may broaden the clinical scope of wearable diagnostics, provided that disease-specific validation, safety assessment, and regulatory requirements are met.

## Conclusion and Perspectives

The rapid maturation of advanced manufacturing and sensing technologies has catapulted wearable electronics into a new era of clinical diagnostics. In this review, we have highlighted how the diversification of transduction modalities, coupled with innovative fabrication and heterogeneous integration strategies, has redefined wearable devices from simple biomarker-recording tools into comprehensive clinical tools. These platforms have accelerated multifaceted disease monitoring, enabling early warning, disease progression tracking, and treatment evaluation across metabolic, cardiovascular, and oncological disorders. However, the transition from laboratory prototypes to mainstream clinical deployment is not merely a matter of accumulating more sensors. To achieve the overarching goal of personalized medicine, future developments must overcome specific hurdles to satisfy four distinct criteria of precision diagnosis: analytical reliability, longitudinal durability, multimodal interpretability, and clinically validated personalization.

With respect to analytical sensitivity and selectivity, the best current wearable platforms already demonstrate that advanced manufacturing and integration strategies can support clinically relevant detection of low-abundance targets across diverse classes of biomarkers, including electrophysiological signals, VOCs, macromolecules, and microorganisms. These achievements establish the feasibility of precision biochemical and physiological monitoring in body-worn formats. However, a substantial gap remains between analytical performance achieved under controlled laboratory conditions and reliable operation in complex, variable biological matrices, where fouling, signal drift, and interference still compromise measurement fidelity. Bridging this gap will require not only further refinement of sensing interfaces, but also the development of more stable transduction mechanisms, more robust anti-interference and biointerface engineering, and wearable omics-oriented strategies that extract clinically meaningful information from interconnected molecular signatures rather than relying on isolated biomarkers alone.

In terms of longitudinal continuity, the strongest examples reviewed here show that wearable systems can now move beyond static or episodic assessment toward repeated or continuous monitoring over days to weeks, thereby enabling detection of deviations from an individual’s physiological baseline, early warning of disease evolution, recognition of acute events, and evaluation of treatment response. This shift is especially important because it connects manufacturing innovation directly to clinical value: Soft mechanics, conformal architectures, and improved packaging are what allow sensors to remain functional and tolerable on the body over extended periods. Yet true longitudinal precision is still constrained by limited operational stability, incomplete biocompatibility, power supply dependence, and the energy burden associated with continuous sensing, computation, and wireless transmission. During prolonged use, proteins, salts, lipids, cells, and bacteria can accumulate at the sensing interface, forming diffusion barriers or nonspecific adsorption layers that reduce sensitivity, slow response kinetics, and increase false-positive or false-negative signals. At the system level, continuous sensing, wireless data transmission, and real-time computation can rapidly deplete limited onboard power, increase latency, and interrupt stable data acquisition, particularly when devices are expected to operate autonomously over days to weeks. Progress toward longitudinal precision will therefore require antifouling and anti-inflammatory interface engineering, hybrid energy harvesting and wireless charging strategies, and near-sensor or in-sensor computing architectures that reduce latency and energy consumption while preserving data quality during prolonged operation. The value of wearable diagnostics becomes even greater when considered through the lens of multi-parameter fusion. The most promising systems reviewed here no longer rely on single-analyte thresholds, but instead combine molecular, electrophysiological, and other complementary signals to improve differential diagnosis and capture more complete representations of pathophysiology. This capacity marks a major step toward precision diagnosis, because clinically actionable interpretation often emerges from the relationships among signals rather than from any single marker alone. Nevertheless, the resulting data streams are large, heterogeneous, and dynamic, and they frequently exceed the interpretive capacity of conventional ML pipelines while also imposing heavy demands on wearable hardware. Future progress will therefore depend on the joint advancement of manufacturing and algorithms: heterogeneous integration that enables synchronized multimodal acquisition, sensor–processor co-design that supports edge intelligence, and more sophisticated ML/DL models capable of robust multimodal feature fusion in real-world environments.

Equally important is the establishment of personalized reference frameworks. The most compelling clinical demonstrations summarized in this review indicate that wearable systems can begin to construct subject-specific baselines and support individualized tracking in ambulatory and home settings, making it possible to distinguish true intraindividual pathological shifts from normal interindividual physiological variation. This capability is central to precision diagnosis, yet it remains fragile in the absence of secure data infrastructures, interoperable standards, and large-scale clinical validation. Data privacy and cybersecurity risks during wireless transmission, fragmented regulatory and industrial standards, the persistent discrepancy between laboratory performance and real-world outcomes, and the high cost and limited lifetime of commercial devices all continue to hinder translation. Accordingly, the path forward must include privacy-preserving data architectures, standardized evaluation frameworks, and scalable manufacturing routes that retain analytical rigor while meeting the practical demands of clinical deployment and commercialization.

Equally critical, yet still insufficiently addressed, is the rigorous alignment of wearable readouts with established clinical reference methods and formal regulatory validation pathways. Wearable outputs should be systematically benchmarked against disease-appropriate diagnostic standards, including 12-lead ECG, Holter monitoring, spirometry, RT-qPCR, ELISA, imaging, histopathology, and commercial CGMs. In practice, however, such benchmarking remains uncommon, and reported performance metrics are often limited to sensitivity and detection limits under controlled laboratory conditions, which are necessary but insufficient to establish clinical equivalence. Lag time, dilution effects, sampling variability, and interindividual differences must be carefully defined before wearable molecular readouts can be interpreted as actionable diagnostic indicators rather than exploratory physiological observations. More broadly, under the EU MDR framework and associated harmonized standards, analytical performance represents only the starting point for clinical translation. Clinical translation requires a staged validation architecture encompassing biocompatibility assessment (ISO 10993), electrical safety (IEC 60601), software lifecycle management (IEC 62304), risk management (ISO 14971), and quality system compliance (ISO 13485), while regulatory classification—ranging from Class IIa to Class III under MDR—is determined by intended clinical function rather than underlying technology, a distinction that most academic prototypes fail to address explicitly [392].

In addition, the emergence and convergence of new monitoring paradigms are further extending the diagnostic range from the body surface to deep tissues. Current wearable platforms already demonstrate that semi-implantable devices, which anchor minimally invasive sensing probes at the tissue–skin interface, can overcome key limitations of surface-based monitoring, including unstable skin–electrode contact, susceptibility to motion artifacts, and limited access to clinically informative biomarkers in ISF. Moving further inward, fully implantable and bioresorbable bioelectronics represent the next frontier. Compared with epidermal and semi-implantable platforms, fully implantable systems position sensing elements directly within target tissues or organs, where they can be fully encapsulated and stably integrated at defined anatomical sites. This configuration enables higher-fidelity local physiological recording while reducing motion artifacts, skin-interfaced instability, and the risk of transcutaneous infection, making it better suited for long-term, continuous, and low-burden in vivo health monitoring and closed-loop intervention [393]. Bioresorbable platforms may further enable wireless, battery-free, and retrieval-free physiological surveillance across diverse organ systems. In parallel, ingestible electronics are emerging as a complementary paradigm for gastrointestinal and systemic monitoring, providing access to luminal biochemical environments that remain inaccessible to external sensors. For practical translation, however, implantable systems must further improve the long-term mechanical integrity of soft implants under cyclic physiological loading, develop energy-harvesting strategies capable of sustainably powering complex in vivo sensing operations, and enhance the reliability and capacity of data acquisition to support AI-driven diagnostic models [394, 395].

Taken together, these observations show that the central question is no longer whether wearable electronics can detect clinically relevant signals, but whether they can do so with sufficient precision to support actionable diagnosis across time, modalities, and individuals. In this sense, the choice of manufacturing strategy is not merely a technical consideration; it is a decisive determinant of whether a wearable platform can achieve clinical-grade precision diagnosis. Future progress will therefore depend on aligning materials engineering, device fabrication, heterogeneous integration, energy management, intelligent analytics, data security, long-term reliability engineering, and clinical validation within a single translational framework. Only through such convergence can wearable diagnostic systems evolve from high-performing laboratory prototypes into robust and trusted platforms for precision and personalized medicine.
